# The effect of GLP-1R agonists on the medical triad of obesity, diabetes, and cancer

**DOI:** 10.1007/s10555-024-10192-9

**Published:** 2024-05-27

**Authors:** Shahad Sabaawi Ibrahim, Raghad Sabaawi Ibrahim, Batoul Arabi, Aranka Brockmueller, Mehdi Shakibaei, Dietrich Büsselberg

**Affiliations:** 1 Weill Cornell Medicine-Qatar, Qatar Foundation, Education City, Doha, 24144 Qatar; 2 Vegetative Anatomy, Institute of Anatomy, Faculty of Medicine, LMU Munich, Pettenkoferstr. 11, D-80336 Munich, Germany

**Keywords:** Ozempic, Semaglutide, Cancer, Diabetes, Obesity, GLP1RA

## Abstract

Glucagon-like peptide-1 receptor (GLP-1R) agonists have garnered significant attention for their therapeutic potential in addressing the interconnected health challenges of diabetes, obesity, and cancer. The role of GLP-1R in type 2 diabetes mellitus (T2DM) is highlighted, emphasizing its pivotal contribution to glucose homeostasis, promoting β-cell proliferation, and facilitating insulin release. GLP-1R agonists have effectively managed obesity by reducing hunger, moderating food intake, and regulating body weight. Beyond diabetes and obesity, GLP-1R agonists exhibit a multifaceted impact on cancer progression across various malignancies. The mechanisms underlying these effects involve the modulation of signaling pathways associated with cell growth, survival, and metabolism. However, the current literature reveals a lack of *in vivo* studies on specific GLP-1R agonists such as semaglutide, necessitating further research to elucidate its precise mechanisms and effects, particularly in cancer. While other GLP-1R agonists have shown promising outcomes in mitigating cancer progression, the association between some GLP-1R agonists and an increased risk of cancer remains a topic requiring more profound investigation. This calls for more extensive research to unravel the intricate relationships between the GLP-1R agonist and different cancers, providing valuable insights for clinicians and researchers alike.

**Supplementary Information:**

The online version contains supplementary material available at 10.1007/s10555-024-10192-9.

## Introduction

 Semaglutide (US brand name Ozempic or Wegovy), a Glucagon-like peptide-1 receptor (GLP-1R) agonist containing the active ingredient semaglutide, is approved by the Food and Drug Administration for its potential therapeutic role in obesity and diabetes mellitus (DM) similar to other agonists within the GLP-1R family such as liraglutide. It not only successfully regulates blood sugar levels [1] but also reduces the appetite and, thereby, the weight of patients [2], especially in individuals with obesity and type 2 diabetes mellitus (T2DM). But why is there even a need for such a medication? And are potential long-term consequences, such as promoting the development or spread of cancer, receiving sufficient attention?

The number of overweight people has been increasing rapidly in recent years due to genetic, socio-economic, lifestyle, and cultural influences. Compared to the past, this not only affects adults but also numerous children and young people. Especially in American and European regions, at least 50% of residents now weigh more than the international standardized body mass index (BMI) recommends [3]. In this relation, a BMI of 30 kg/m^2^ or higher is classified as obese [4], whose causes lie in excessive calorie intake or reduced energy expenditure, and modern lifestyles promote both a lack of exercise and an unhealthy diet. Therefore, both these lifestyle factors and obesity itself represent a significant risk factor for metabolic disease T2DM [3, 5].

DM is one of the most common metabolic diseases worldwide, with around 530 million people currently affected and an estimated 1.3 billion diabetes patients in 2050. The most common disease forms are the well-known types 1 and 2, severe diseases with chronic hyperglycemia [6]. While type 1 often manifests as an autoimmune insulin deficiency during childhood, T2DM predominantly develops throughout life. Epigenetic modifications trigger a genetic predisposition, which leads to insulin resistance [7]. Concretely, tobacco smoking, alcohol consumption, unbalanced diet, low fitness, increased BMI as well as unhealthy environmental influences are the key risk factors for the development of T2DM, and this form accounts for 96% of all DM cases [6]. Due to the disrupted metabolic processes, numerous signaling pathways become dysregulated, resulting in epigenetically induced inflammation. This triggers the development of malignant tumors in different tissues, for example, the colorectal, liver, or breast cancer [8–10], creating a medical triad consisting of overweight, DM, and oncogenesis. Due to the close connection between this pathogenesis, it must be considered that drug manipulation of one of the diseases can also affect the other components of this interaction.

Therefore, this review presents the triangle relationship of obesity, DM as well as cancer development and elucidates the treatment of overweight-associated T2DM with semaglutide, focusing on the question of whether the development of these conditions can be combated or if any severe side effects should be considered of this therapy.

## The medical triad

Obesity, as well as DM, are conditions associated with a heightened risk of various cancers, including pancreatic, colorectal, breast, or liver cancer, and an increased mortality risk also accompanies them [8]. The link between these health issues and cancer risk is attributed to imbalances in the interaction of complex metabolic processes.

Obesity can be prevented by regulating body weight, dieting, and exercising [9] and although obesity is preventable, the increase in body fat allows the progression of metabolic diseases. Especially interesting, these metabolic diseases are associated with approximately 20% of cancer cases [10, 11]. Obesity induces metabolic disturbances in adipose tissue, influencing the release of hormones, adipokines, inflammatory cytokines, growth factors, enzymes, and free fatty acids [12]. Notably, each 5% increase in BMI is estimated to correlate with a 10% rise in cancer-related deaths [13]. The altered physiology of adipose tissue in obesity releases metabolic substrates contributing to tumor cells’ proliferation, invasion, and metastasis. Two critical factors in this association are pro-inflammatory cytokines and adipokines. Pro-inflammatory cytokines produced by adipose tissue support tumor-promoting intercellular crosstalk in the tumor microenvironment [14], thus enhancing tumor cell progression, angiogenesis, and invasion as essential requirements for metastasis [15]. For example, breast and colorectal cancer progression was attributed to obesity [16], and in liver and gallbladder cancers, 51% of the cases are caused by this overweight disease [16]. However, calorie deficit, active lifestyle, behavior therapy, and drug therapy reduce inflammatory markers and regulate insulin levels commonly associated with cancer progression [17, 18].

Adipokines such as adiponectin and leptin, derived from adipose tissue, play pivotal roles. The excessive expansion of adipose tissue in obesity disrupts adipokine secretion, fostering chronic low-grade inflammation and thereby contributing to the onset of metabolic disorders like obesity and T2DM. Adiponectin, inversely correlated with BMI, exhibits protective effects against carcinogenesis based on *in vitro* models. Leptin, implicated in inflammatory, mitogenic, and pro-angiogenic pathways, has been associated with breast cancer development, with studies indicating that inhibiting leptin signaling reduces the growth of breast cancer induced by carcinogens [12].

Moreover, high adiposity contributes to elevated serum estrogen levels, which, in excess, can promote tumor development by causing DNA damage, stimulating angiogenesis, and fostering cellular proliferation [19].

The presence of hyperglycemia and hyperinsulinemia in T2DM [20], leading to metabolic dysfunction, can also contribute to the proliferation and migration of cancer cells [21]. Cancer progression due to hyperglycemia was reported in multiple cancers, including breast, colorectal, brain, and pancreatic cancer [22–25]. Insulin is responsible for activating insulin receptors and insulin growth-like receptors. Moreover, elevated insulin levels resulting from hyperinsulinemia trigger insulin-like growth factor (IGF) signaling, activating key pathways such as phosphoinositide 3-kinase (PI3K)/protein kinase B (Akt)/mammalian target of rapamycin (mTOR) and mitogen-activated protein kinase (MAPK) [26]. These pathways, in turn, facilitate cancer cell growth, survival, motility, and resistance to drugs. Furthermore, it has long been theorized that cancer cells exhibit heightened glucose uptake and rely on glucose as a primary fuel for proliferation because it is a substrate cancer cells use as an energy source in aerobic glycolysis, resulting in tumor progression [27]. This phenomenon, known as the Warburg effect [28, 29], is attributed to damaged mitochondria in cancer cells. Hence, anticancer therapy may include antidiabetic drugs targeting glucose metabolism and metabolic pathways that decrease the glucose uptake in cancer cells [30].

In summary, the intricate interplay of metabolic abnormalities, inflammatory responses, and hormonal influences in obesity and diabetes underscores their significant impact on cancer risk and mortality.

## The treatment complexities

**Fig. 1:**
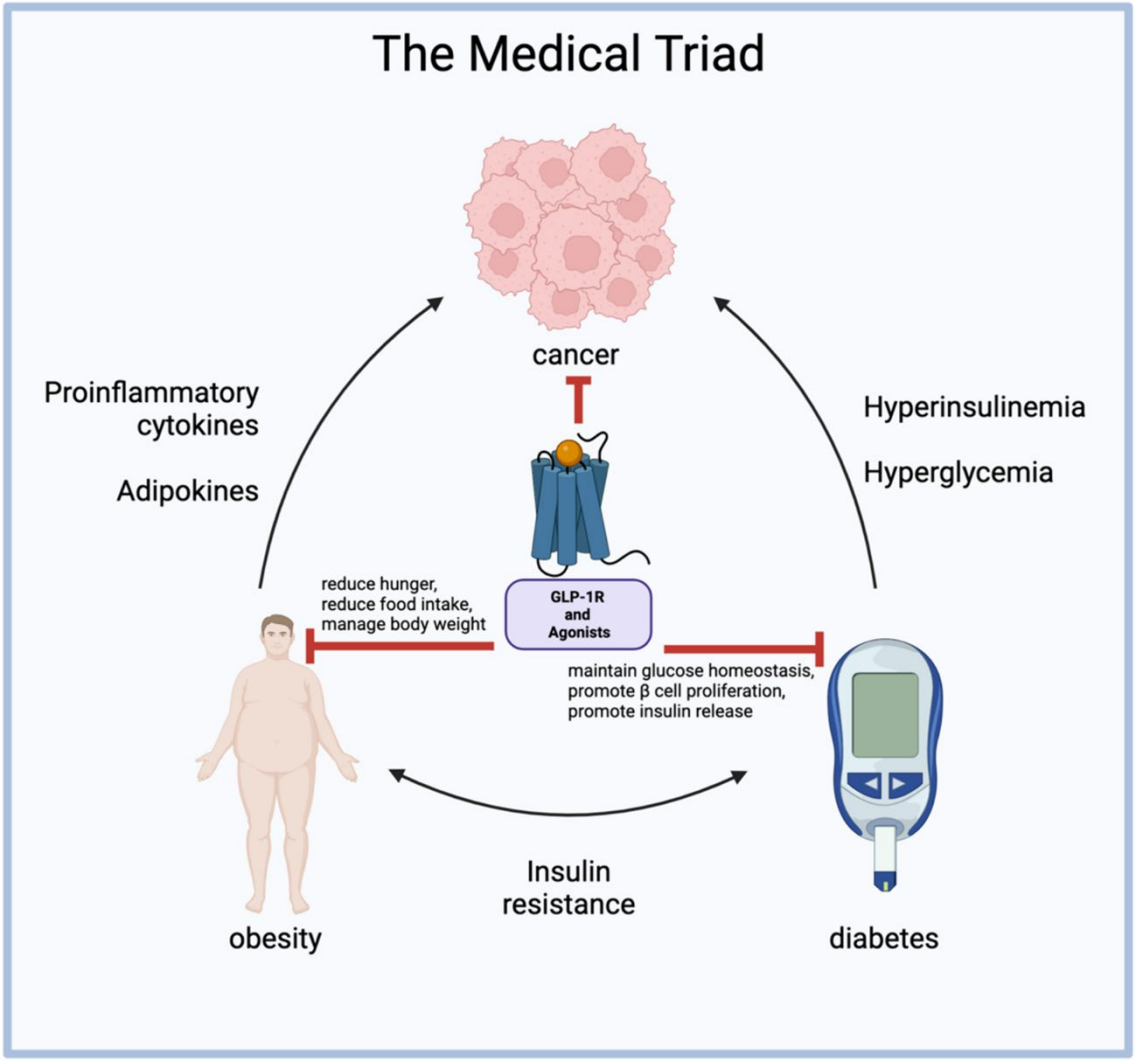
The link between cancer, obesity, diabetes, and GLP-1R’s effect on them. Generated using BioRender

The treatment of cancer in individuals who are both obese as well as diabetic poses significant challenges due to the intricate interplay between these conditions, and addressing these challenges involves navigating a complex landscape (Fig. 1). One noteworthy obstacle is the potential need for higher chemotherapy doses in obese patients based on their body weight. However, this approach carries the inherent risk of heightened side effects and drug toxicity. In the case of obese individuals, an elevated BMI has been linked to increased interactional displacement, primarily stemming from the continuous movement of the skin and subcutaneous adiposity [31]. This displacement shift raises concerns about a potential reduction in the radiation dose reaching the target cells, leading to apprehensions about inadvertently overdosing patients with radiation and chemotherapy [31, 32].

Moreover, managing diabetes during cancer treatment is a crucial aspect often overshadowed by the primary focus on cancer therapies. Chemotherapy, in particular, can influence blood sugar levels, causing fluctuations that need careful consideration. Additionally, the use of corticosteroids alongside chemotherapy to mitigate severe nausea and vomiting introduces another layer of complexity [33]. For diabetic patients, this poses a substantial threat, as corticosteroids are known to induce hyperglycemia [34]. The combination of decreased glucose uptake by the muscle and decreased glycogenesis further contributes to hyperglycemic conditions and complicates the already challenging task of treating cancer in individuals managing diabetes.

GLP-1R plays a significant role in the triad, making it an appealing target for treatment (Fig. 1). Specifically, GLP-1R emerged as an important pharmacological target for addressing T2DM, as it actively contributes to maintaining glucose homeostasis while promoting both β cell proliferation and insulin release [35]. The impact of GLP-1R agonists such as semaglutide extends beyond diabetes control: they play a multifaceted role in regulating blood glucose levels by reducing hunger, moderating food intake, and managing body weight [36]. Notably, GLP-1R agonists inhibit cancer progression in some malignant tumors [37–40].

## Glucagon-like peptide-1 receptor

**Fig. 2:**
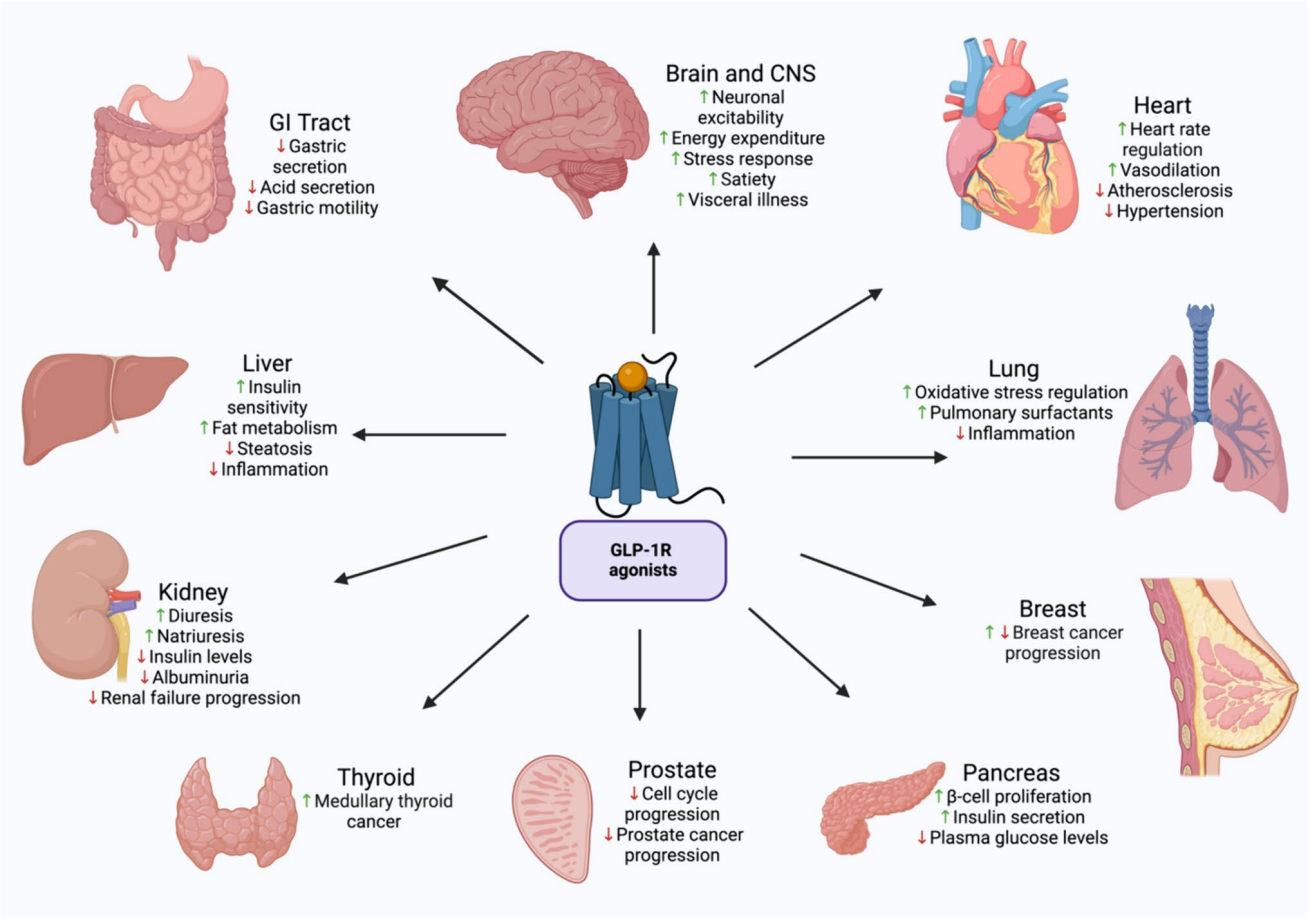
Effects of GLP-1RAs on organs. GLP-1R and agonists decrease hypertension, atherosclerosis, inflammation, plasma glucose levels, prostate cancer progression, insulin levels, albuminuria, renal failure progression, steatosis, gastric and acid secretion, and gastric motility levels amongst various organs in the body. GLP-1R and agonists increase heart rate regulation, vasodilation, oxidative stress regulation, pulmonary surfactants, β cell proliferation, insulin secretion and sensitivity, medullary thyroid cancer, diuresis, natriuresis, fat metabolism, neuronal excitability, energy expenditure, stress response, satiety, and visceral illness. For breasts, GLP-1R and agonists lead to an increase or decrease in breast cancer progression. Generated using BioRender

The GLP-1R comprises seven hydrophobic transmembrane domains and a hydrophilic extracellular domain [41]. These receptors are expressed in multiple tissues, including the lung, stomach, intestine, liver, kidney, heart, pancreas, and regions in the central nervous system. Hence, it is a significant target of small-molecule drugs for signaling modulation (Fig. 2) and treating various diseases [42]. The activation of GLP-1R is associated with glucose-induced insulin secretion and inhibition of α-cell glucagon release [43]. In addition, the activation of GLP-1R results in a cascade that activates adenyl cyclase via Gα_s_, resulting in an increased secretion of cyclic adenosine monophosphate (cAMP) secretion and activating cAMP-dependent protein kinase (PKA). GLP-1R can also couple with adenyl cyclase using other Gα_s_ subtypes such as Gα_i_ and Gα_q_ [44, 45]. An influx in calcium is reported upon activation of GLP-1R, and in combination with the activation of PKA, this results in insulin secretion [46].

GLP-1R reduces the inflammatory-induced response in the lungs, regulates oxidative stress and pulmonary function, and decreases excessive mucus production [47, 48]. Furthermore, GLP-1R affects the mucosal membrane in the gastric tract by decreasing gastric secretions, which restrict gastric acid secretions and motility [49–51]. GLP-1R causes a reduction in hepatic steatosis and inflammation and increases fat metabolism mediated by increasing hepatic insulin sensitivity [52–54]. The GLP-1R directly influences renal functions by enhancing diuresis and natriuresis [55]. In diabetic kidney disease patients, a reduction in insulin levels, albuminuria, and the progression of renal failure were observed as a GLP-1R effect [56, 57]. Moreover, GLP-1R is found in the heart and blood vessels and is beneficial in heart rate, vascular endothelium, atherosclerosis, and hypertension [58, 59]. Since GLP-1R is a target in diabetes mellitus, it enhances the proliferation of β-cells and insulin secretion in the pancreas and reduces plasma glucose levels [60, 61]. The significant role of GLP-1R in the brain is to regulate metabolic processes, energy expenditure, and neuronal excitability [62, 63]. It also causes stress responses, satiety, and vi0sceral illness’ in the central nervous system [63]. As an alarming finding, the activation of GLP-1R has been associated with developing thyroid cancer [64, 65].

On the other hand, the activation of GLP-1R in the prostate attenuates cell proliferation and the progression of prostate cancer [66]. Although GLP-1R’s role is not fully understood or investigated in breast tissue when activated by different agonists, it may increase or decrease the progression of breast cancer [67, 68]. The extensive role of GLP-1Rs and their agonists in other organs and tissues are shown in the overview in Fig. 2.

## Glucagon-like peptide-1 receptor agonists

GLP-1R is targeted by GLP-1R agonists, which regulate diabetes and obesity. Although diabetic patients commonly use metformin, GLP-1R agonists serve as a beneficial therapeutic option for diabetic patients with metformin intolerance. GLP-1R agonists mimic hormones that activate biological responses in GLP-1R. GLP-1R agonists fall under two groups: human GLP-1 backbone agents and exendin-4 backbone agents. Dulaglutide, albiglutide, liraglutide, and semaglutide are human GLP-1 backbone agents. Exenatide and lixisenatide are classified as Exendin-4 backbone agents. In addition, semaglutide, liraglutide, and tirzepatide are GLP-1R agonists that are FDA-approved [69]. Albiglutide was discontinued for low prescription rates rather than safety concerns [70, 71]. All GLP-1R agonists have the same mode of function but differ in half-life duration. Exenatide has a half-life of 3.3–4 h, and one dose is seen to be insufficient, so it is taken twice daily. Lixisenatide and liraglutide have half-lives of 2.6 h and 12.6–14.3 h, respectively and are taken once daily. Dulaglutide, albiglutide, and semaglutide have half-lives that range from 4.7 to 5.5, 5.7–6.8, and 5.7–6.7 days, respectively [72].

GLP-1R agonists exhibit protective and regulatory effects on blood glucose levels but have been linked to the inhibition of tumor cell proliferation in most cancer cases, as seen *in vitro* and summarized in Table 1 [51, 73]. The fact that GLP-1R agonists are sometimes introduced if the patient is intolerant to metformin or metformin is contraindicated, or when patients on metformin are not achieving their HbA1c goals [71] represents essential background information here.

**Table 1:** GLP-1R agonist’s effect on cancer in *in vitro* studies

Cancer type	Cell line	GLP-1R agonist	Concentration and duration	Serum concentration of drug	Results	References
Colorectal cancer	LOVO	Liraglutide	10^−5^ mol/L, 10^−8^ mol/L, 10^−11^ mol/L for 24, 48, or 72 h	Ν/Α	↓ PI3K ↓ Akt ↓ mTOR ↓ Proliferation ↓ Migration ↓ Invasion ↑ Apoptosis	[74]
Thyroid cancer	CA-77	GLP-1 (7–37) agonists	10^−8^ M from 3–48 h	Ν/Α	↑ cAMP ↑ CGRP ↑ CT	[75]
Breast cancer	MCF-7, MDA-MB-231, KPL-1	Exendin-4	0.1–10 nM for 0–3 days	0.44 ± 0.07 ng/mL	↓ Proliferation ↓ NF-κB activation ↑ GLP-1R activation	[76]
Breast cancer	MCF-7, MDA-MB-231, KPL-1	Exendin-4 combined with metformin	10 nM for 0–3 days	Ν/Α	↓ Proliferation ↑ GLP-1R activation	[77]
Breast cancer	MCF-7	Exendin-4	0.25, 0.5, 1, 1.5, 2, 3, 5, 7.5, or 10 µM for 72 h	5 µM	↓ Migration ↓ Invasion ↓ Colony formation ↓ Proliferation ↑ Apoptosis	[78]
Breast cancer	MCF-7, MDA-MB-231, MDA-MB-468	Exendin-4	1, 10, or 50 nM for 14 days	Ν/Α	↓ Colony formation ↓ Cyclin D1 ↓ p-Akt ↑ p53 ↑ p21 ↑ p38 ↑ cAMP	[79]
Breast cancer	4T1, MCF-7, MDA-MB-231, MDA-MB-468, BT483, ZR751	Liraglutide	0, 10, 100, or 1000 nM for 24, 48, or 72 h	Ν/Α	↑ GLP-1R expression ↑ Proliferation ↑ Migration ↑ ROS generation ↑ NOX4 expression ↑ VEGF	[67]
Prostate cancer	LNCap, PC3, ALVA-41, DU145	Exendin-4	0.1–10 nM for 0, 24, 48, 72, or 96 h	N/A	↓ ERK-MAPK pathway ↓ Proliferation ↑ cAMP	[80]
Prostate cancer	LNCap	Liraglutide combined with Docetaxel	10, 20, 40, or 80 µM for 48 h	Ν/Α	↑ G2/M phase arrest ↑ Apoptosis ↓ p-ERK1/2 ↓ p-Akt	[81]
Prostate cancer	LNCap	Exenatide or liraglutide	0, 1, 10, or 100 nM for 24 h	Ν/Α	↓ Proliferation ↓ Cell viability ↑ Apoptosis ↑ Bax/Bcl-2 ratio ↑ p38 MAPK activation	[82]
Pancreatic cancer	PANC-1, MiaPaCa-2, PANC	Liraglutide	0, 10, 100, or 1000 nM for 48 h	Ν/Α	↑ GLP-1R expression ↑ PKA expression ↑ cAMP ↑ Apoptosis ↑ Bax ↑ Caspase-3 ↓ Growth ↓ Colony formation ↓ NF-κB expression	[68]
Pancreatic cancer	MIA PaCa-2, PANC-1	Liraglutide	0, 10, 50, 100, 500, or 1000 nM for 72 h	Ν/Α	↓ Colony formation ↓ Viability ↓ Migration ↓ Invasion ↓ Proliferation ↓ p-Akt	[83]
Colon cancer	CT26	Exendin-4	5 or 50 nM	Ν/Α	↓ p-ERK1/2 ↓ GSK3 ↑ cAMP ↓ Proliferation ↑ Apoptosis ↓ Colony formation ↓ Viability	[84]
Ovarian cancer	SKOV3, OVCAR3, OVCAR4, A2780, ES-2	Exendin-4	0, 1, 10, or 100 nM for 96 h	Ν/Α	↓ Proliferation ↓ Colony formation ↓ Migration ↓ Invasion ↓ p-Akt ↑ Apoptosis	[85]

The effect of GLP-1R agonists liraglutide and exendin-4 was examined *in vitro* on LOVO, CA-77, MCF-7, MDA-MB-231, KPL-1, MB-468, 4TI, BT483, ZR751, LNCap, PC3, ALVA-41, DU145, LNCap, PANC-1, MiaPaCa-2, PANC, CT26, SKOV3, OVCAR3, OVCAR4, A2780, and ES-2 cell lines that are expressed in several cancers including colorectal, pancreatic, thyroid, breast, prostate, ovarian, and colon (Table 1).

Liraglutide concentrations of 10–1000 nM implemented for 24–72 h showed an increase in apoptosis, G2/M phase arrest, Bax/Bcl-2 ratio, p38 MAPK activation, PKA expression, cAMP, caspase-3, GLP-1R expression, migration, ROS generation, NOX4 expression, VEGF, and proliferation of breast cancer in only one study. Nonetheless, a decrease in PI3K, Akt, mTOR, proliferation, migration, invasion, p-ERK1/2, growth, colony formation, and inflammation represented by nuclear factor κB (NF-κB) expression, cell viability, and an overall decrease in proliferation was noted with applying liraglutide (Table 1).

**Fig. 3:**
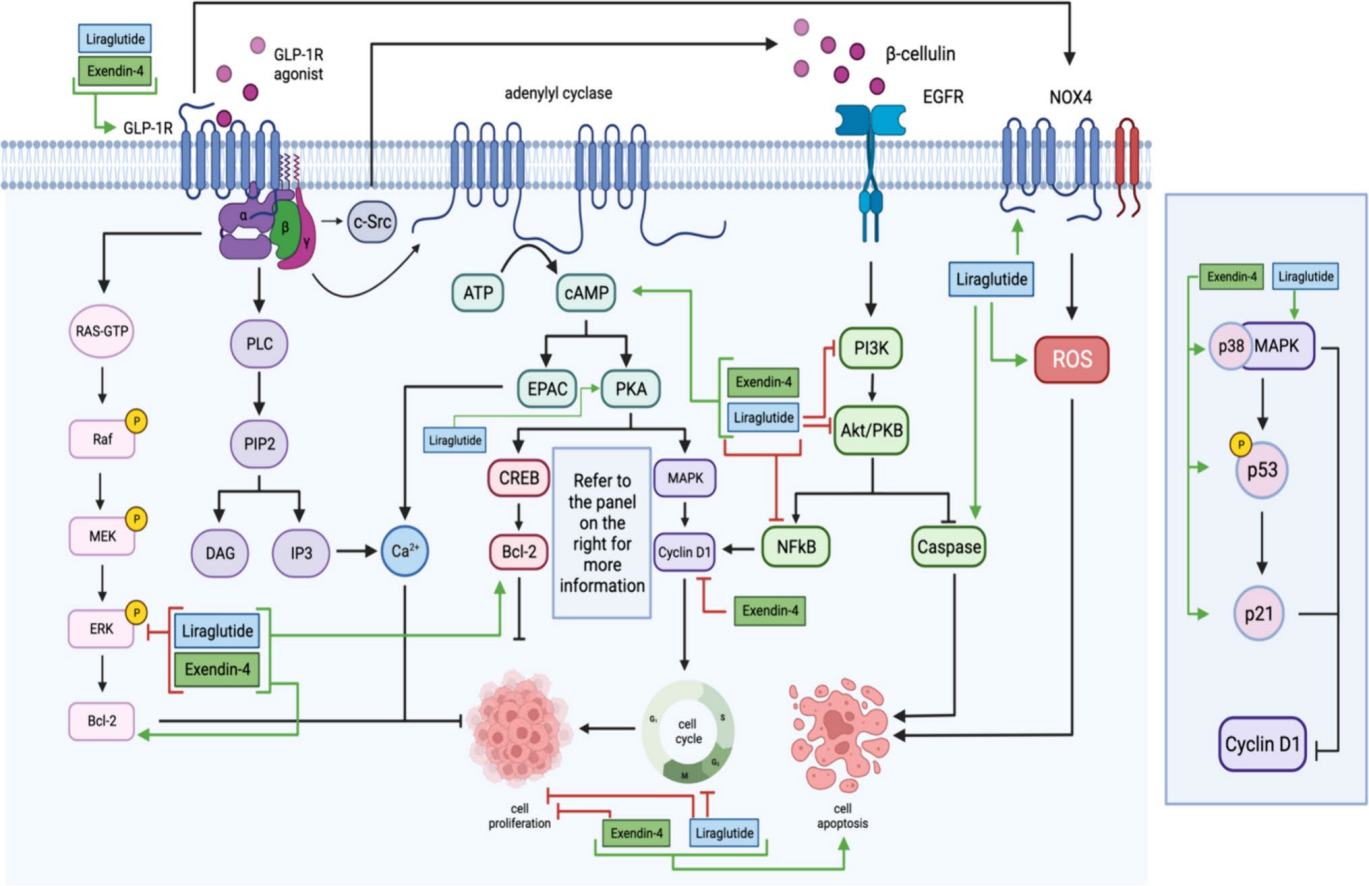
Liraglutide and Exendin-4 decrease NF-κB, cell proliferation, and phosphorylated ERK in cancer. Liraglutide decreases Pl3K, Akt/PKB, and cell division while increasing GLP-1Rs, NOX4, ROS, caspase, MAPK, cAMP, Bcl-2, PKA, and cell apoptosis levels. Exendin-4 decreases Cylin D1 while increasing GLP-1Rs, MAPK, p21, p53, cAMP, Bcl-2, and cell apoptosis. Generated using BioRender

In addition, exendin-4 at 0.1–100 nM concentrations for 24–96 h increased GLP-1R activation, p53, p21, p38, cAMP, Bax/Bcl-2 ratio, and p38/MAPK activation. A reduction in proliferation, NF-κB activation, migration, invasion, migration, colony formation, Cyclin D1, p-Akt, ERK-MAPK pathway, cAMP, and GSK3 accompanied this increase (Table 1). Figure 3 shows an overview of the mentioned effects of the two GLP-1R agonists and their influence on numerous signaling pathways.

**Fig. 4:**
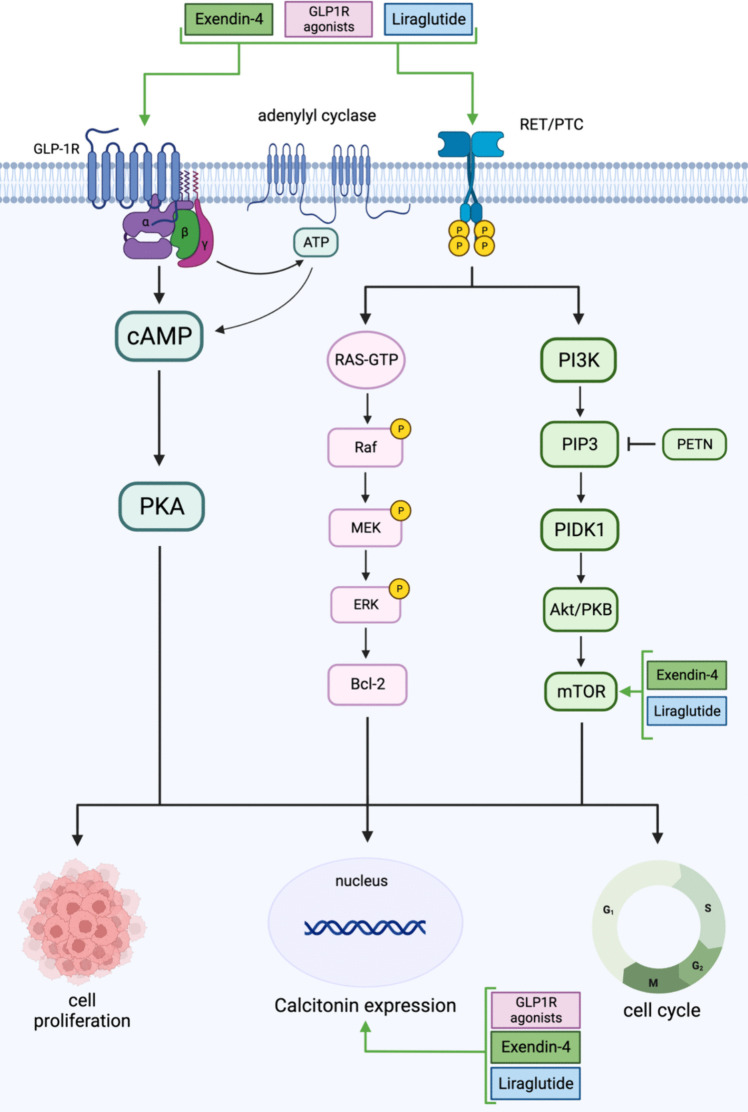
GLP-1R agonists were studied in thyroid cancers. Both Liraglutide and Exendin-4 increase the levels of GLP-1R, RET/PTC, mTOR, and calcitonin expression amongst patients with thyroid cancer. Generated using BioRender

Some of the significant GLP-1R agonists studied other than semaglutide are liraglutide and exendin-4. Both agonists affect cancer *in vitro* by decreasing the proliferation and metabolic pathways at varying concentrations. Liraglutide and exendin-4 are initially antidiabetic drugs but can affect tumorigenesis, suggesting GLP-1R agonists as a potential treatment for different cancers as found *in vivo* (Table 2). Notably, an increase in calcitonin (Fig. 4), particularly observed in thyroid cancer, indicates cancer development [75], given its role as a tumor marker in medullary thyroid neoplasia [86]. More research is needed to understand the effect of other agonists, as cancer research is lacking. GLP-1R agonists are typically combined with metformin or other antidiabetic drugs. The combination of GLP-1R agonists with each other has not been studied previously, suggesting that due to their similar mode of action, there would not be an enhancement in therapy from this combination.

**Table 2:** GLP-1R agonist’s effect on cancer in* in vivo* studies

Cancer type	Cell line	GLP-1R agonist	Concentration and duration	Results	References
Thyroid cancer	CD-1 wild-type mice	Liraglutide and exenatide	0.03, 0.3, or 3.0 mg/kg for 13 weeks	↑ Calcitonin ↑ C-cell hyperplasia ↑ GLP-1R ↑ mTOR activation ↑ pS6	[87]
Breast cancer	MCF-7 cells into athymic nude mice	Exendin-4 and exandin	300 pmol/kg body weight/day Ex-4 or 3 nmol/kg body weight/day exendin for 6–9 weeks	↓ Tumor size ↓ Proliferation ↓ Ki67 ↓ NF-κB activation ↑ Serum insulin	[76]
Breast cancer	MCF-7 cells into athymic mice	Exendin-4 combined with metformin	300 pmol/kg body weight/day for 8 weeks	↓ Tumor volume ↓ Tumor weight ↓ Ki67 ↑ GLP-1R	[77]
Breast cancer	MDA-MB-468 and MDA-MB-231 cells into athymic nude mice	Exendin-4	500 ng or 2 µg per day for 6 weeks	↓ Tumor weight ↓ Tumor size	[79]
Breast cancer	4T1 cells in BALB/cfC3H mice	Liraglutide	400 µg/kg for 2 weeks	↑ Tumor volume ↑ Metastasis	[67]
Prostate cancer	LNCap cells into athymic mice	Exendin-4	24 nmol/kg body weight/day or 300 pmol/kg body weight/day	↓ Tumor size ↓ P504S, prostate cancer marker ↓ Ki67 ↓ Proliferation ↓ p-ERK-MAPK	[80]
Prostate cancer	LNCap cells into athymic mice	Exendin-4 combined with metformin	300 pmol/kg body weight/day for 6 weeks	↓ Tumor volume ↓ Tumor weight ↓ Ki67 ↓ P504S ↓ Proliferation	[88]
Pancreatic cancer	PANC-1 cells into nude mice	Liraglutide	0.2 mg/kg twice daily for 4 weeks	↑ Chemosensitivity ↑ Bax ↓ Tumor volume ↓ Tumor weight ↓ Ki67	[68]
Pancreatic cancer	MIA PaCa-2 in male athymic nude mice	Liraglutide	0.2 mg/kg for 4 weeks	↓ Tumor growth	[83]
Colon cancer	CT26 cells into BALB/c mice	Exendin-4	10 nmol/kg for 2 weeks	↑ Apoptosis ↓ Proliferation	[84]
Ovarian cancer	SKOV-3 cells into female nude mice	Exendin-4	500 ng/day or 2 µg/day for 28 days	↓ Tumor size ↓ Tumor weight ↓ Tumor volume	[85, 89]
Intestinal cancer	Apc(Min/+) mice	Exendin-4	For 1 month	↑ Small bowel weight ↑ Small bowel length ↑ Large bowel weight	[89]
Liver cancer	N/A	Liraglutide	N/A	↓ Body weight ↓ Fasting blood glucose ↓ Tumor lesions ↓ Fat deposition ↑ Insulin-positive β-cells	[90]

The effect of GLP-1R agonists liraglutide and exendin-4 was examined *in vivo* on CD-1, MCF-1, MDA-MB-468, MDA-MB-231, 4T1, LNCap, PANC-1, MIA PaCa-2, CT26, SKOV-3, and Apc(Min/+) cell lines expressed in cancers such as thyroid, breast, prostate, pancreatic, colon, ovarian, intestinal, and liver (Table 2).

Both liraglutide and exendin-4 decrease the size, weight, and proliferation of pancreatic, breast, prostate, ovarian, intestinal, liver, and colon cancer. Liraglutide slows down and sometimes even inhibits tumor growth of cancers in mice in *in vivo *studies. Moreover, it downregulates the protein levels of cell proliferation marker PCNA, decreases cell viability and number, and upregulates the protein levels of pro-apoptotic markers [91]. In addition, this GLP-1R agonist activates AMPK, inhibiting the proliferation of various cancerous cells, making it a promising cancer treatment [92].

Exendin-4 significantly inhibits different cell lines and induces apoptosis through the mechanism modification of apoptosis-related genes, which plays a role in extrinsic pathways and cell survival genes [78]. Specifically in colon cancer and prostate cancer, exendin-4 increased intracellular cAMP levels while inhibiting glycogen synthase kinase 3 and ERK-MAPK activation, leading to an increase in apoptosis [93]. Moreover, exendin-4 inhibits migration, cell invasion, and colony formation of many cancers, making it a possible treatment for malignant cells, specifically in prostate cancer. Exendin-4 suppresses cell proliferation through the inhibition of ERK-MAPK [80]. Lastly, a combination of exendin-4 and metformin has been shown to attenuate different forms of cancer at a more noticeable rate [94, 95].

## The popular GLP-1R agonist semaglutide

Semaglutide is a long-acting GLP-1R agonist structurally similar to GLP-1 but resistant to proteolytic cleavage [96] and is given as a subcutaneous injection to patients with T2D. This is because, in comparison to GLP-1, semaglutide has two amino acid substitutions, which makes it less vulnerable to degradation by the proteolytic enzyme dipeptidyl peptidase-4 (DPP-4) [97] and gives it the distinct advantage of increased albumin affinity [96]. Moreover, due to these substitutions, semaglutide has a prolonged half-life of approximately 168 h, which is impressive given the knowledge that native GLP-1 has a half-life of 2 min, in comparison [98, 99]. This progress in scientific development enables a 1-week administration of the drug, making them an efficient alternative to other antidiabetic drugs, such as metformin, which requires two daily doses, and the updated metformin, which requires daily doses [100, 101]. Furthermore, semaglutide is superior to other GLP-1R agonists, such as liraglutide, which uses similar pharmacological mechanisms but only has a half-life of 13 h and therefore has to be injected subcutaneously daily. This is underscored by the results of a clinical trial comparing the development of obese adults who received either semaglutide or liraglutide in addition to nutritional advice and physical activity, and significantly greater weight loss was achieved in the semaglutide group [102].

Semaglutide, as a GLP-1R agonist, works very similarly to GLP-1 by potentiating glucose-stimulated insulin secretion from the pancreatic β-cells while suppressing glucagon secretion by pancreatic α-cells [103]. Therefore, antidiabetic medication decreases blood sugar levels, reduces body weight through a reduction in appetite [94], and lowers glycated hemoglobin (HbA1c), all while having a low risk of causing hypoglycemia [104]. Although GLP-1R agonists exhibit protective and regulatory effects on blood glucose levels, they have been positively correlated with tumor progression in patients with diabetes [51, 73].

## Semaglutide’s role in the medical triad of diabetes, obesity, and cancer

The prevalence of T2DM has increased significantly in the past decades. It is likely to be the fifth most common cause of death, following an 8% attribution to the mortality rate in the USA, Canada, and the Middle East [105]. Patients with T2DM tend to secrete less insulin following a glucose-heavy meal possibly due to decreased levels of glucagon-like peptide-1 (GLP-1) [96]. Characterized by insulin resistance, gradual progressive loss of insulin secretion by β-cells, and being heavily driven by being overweight or obese [98], T2DM leads to hyperglycemia, excessive urine production, increased risk of cardiovascular disease, and changes in energy metabolism [106]. To lessen the effects of these symptoms, patients with T2DM are encouraged to improve their eating lifestyles and increase their physical activity [107].

Nevertheless, despite the positive outcomes of exercise and diet, recommended glycemic levels (e.g., HbA1c < 7.0%, 53.0 mmol/mol for nonpregnant adults) [108] may sometimes be challenging to achieve. Therefore, the addition of glucose-lowering agents is recommended by the American and European Diabetes Associations to control and/or minimize the risk of cardiovascular disease and microvascular complications [103]. In this regard, semaglutide is becoming increasingly important, as demonstrated in Table 3, which summarizes the previous results of phase 3 clinical trials.

**Table 3:** Semaglutide’s effect on type 2 diabetes and obesity in phase 3 trials

Sustain number	Number of participants	Concentration and treatment duration	Monotherapy vs. combined therapy	Results	Reference
1	388	0.5, or 1.0 mg for 30 weeks	Monotherapy	↓ HbA1c ↓ Body weight ↑ Nausea ↑ Diarrhea	[109]
2	1231	0.5, or 1.0 mg for 56 weeks	Both, combined with 100 mg sitagliptin	Monotherapy: ↓ HbA1c ↓ Body weight Both: ↑ Hypoglycemia ↑ Nausea ↑ Diarrhea	[110]
3	813	1.0 mg for 56 weeks	Monotherapy	↓ HbA1c ↓Fasting insulin ↓Insulin resistance ↓Plasma glucagon ↓ Body weight ↑ Gastrointestinal adverse events ↑ Neoplasms	[111]
4	1089	0.5, or 1.0 mg for 30 weeks	Combined with metformin alone or with sulfonylurea	↓ HbA1c ↓ Body weight	[112]
5*	397	0.5, 1.0 mg for 30 weeks	Combined with basal insulin	↓ HbA1c ↓ Body weight ↑ Neoplasms ↑ Pancreatic cancer	[97]
6	3297	0.5, 1.0 mg for 104 weeks	Monotherapy	↓ HbA1c ↓ Body weight ↓Cardiovascular death ↓ Nonfatal stroke and myocardial infarction ↓ Mean systolic blood pressure ↑ Diabetic retinopathy complications	[113]
7	1201	0.5, 1.0 mg for 24 weeks	Monotherapy	↓ HbA1c ↓ Body weight	[114]
8	788	1.0 mg for 52 weeks	Combined with metformin	↓ HbA1c ↓ Body weight ↑ Gastrointestinal adverse events	[115]
9	302	1.0 mg for 37 weeks	Combined with sodium-glucose cotransporter-2 (SGLT-2) inhibitors and metformin or sulfonylurea	↓ HbA1c ↓ Body weight ↑ Gastrointestinal adverse events	[116]
10	577	1.0 mg for 30 weeks	Combined with oral antidiabetic drugs	↓ HbA1c ↓ Body weight ↓ FPG ↓ SMBG ↑ Gastrointestinal adverse events ↑ Improvement in total cholesterol and triglycerides	[117]
11	1748	1.0 mg for 52 weeks	Combined with metformin and insulin glargine	↓ HbA1c ↓ Body weight ↓ Systolic blood pressure ↑ Gastrointestinal adverse events	[118]
FORTE	961	1.0 or 2.0 for 40 weeks	Combined with metformin and with or without sulfonylurea	↓ HbA1c ↓ Body weight ↓ Blood pressure ↑ Gastrointestinal adverse events	[119]
Japan	308	0.5 mg, or 1.0 mg for 30 weeks	Monotherapy	↓ HbA1c ↓ Body weight ↓ Blood pressure ↓ VLDL cholesterol and triglycerides ↑ Treatment of emergent adverse events	[120]
Japan	601	0.5, or 1.0 mg for 63 weeks	Both monotherapy and combined with an oral antidiabetic drug	↓ HbA1c ↓ Body weight ↓ Insulin ratios ↓ Blood pressure ↓ All lipids (except free fatty acids and HDL cholesterol) ↑ Treatment of emergent adverse events ↑ Pancreatic enzymes	[121]
China	868	0.5, or 1.0 mg for 30 weeks	Combined with metformin	↓ HbA1c ↓ Body weight ↓ Systolic blood pressure ↓ Total cholesterol ↑ Gastrointestinal adverse events ↑ Amylase and lipase	[122]

*Represents a single case

Semaglutide was tested in clinical trials on individuals with obesity and diabetes. Phase three of the clinical trials was conducted using semaglutide concentrations ranging from 0.5 to 2.0 mg for 30–104 weeks. A decrease in body weight, blood pressure, HbA1c, fasting insulin, insulin resistance, plasma glucagon, total cholesterol, lipids, FPG, SMBG, cardiovascular death, nonfatal stroke, and myocardial infarction are observed with the use of semaglutide in monotherapy or in combination with other antidiabetic drugs. (Table 3). However, an increase in gastrointestinal adverse events, diarrhea, hypoglycemia, nausea, neoplasm, treatment-emergent adverse events, pancreatic enzymes, diabetic retinopathy complications, and pancreatic cancer was observed to accompany the treatment using semaglutide. Several clinical trials implemented a combination of semaglutide treatments with sulfonylurea, sitagliptin, basal insulin, metformin, or other antidiabetic drugs (Table 3).

Altogether, clinical trials in phase 3 that applied semaglutide in monotherapy and combined with other antidiabetic drugs yielded similar results. However, suppose semaglutide is reduced to $1711.03 per year. In that case, it will be considered cost-effective and preferable treatment compared to other GLP-1R agonists and antidiabetic drugs [123]. The decrease in HbA1c caused by semaglutide is accompanied by a reduction in body weight, which is unique to semaglutide as an antidiabetic medication. Therefore, injections of semaglutide can assist individuals in maintaining a healthier lifestyle with a decrease in the rate of potential cardiovascular diseases [124]. The effect of semaglutide alone is sufficient to the result in positive outcomes. Still, these positive results can be enhanced when in combination with other drugs, such as metformin, which allows for an effective treatment plan with no increase in adverse symptoms usually seen with the treatment of semaglutide alone [121, 122, 125, 126]. Furthermore, the possible promotion of cancer cell growth is increasingly being discussed with contradictory results (Table 4) that require clarification.

**Table 4:** Semaglutide’s involvement with cancer as an adverse effect

Cancer type	Concentration and duration	Number of participants	Number of events in placebo	Number of events caused by semaglutide	Reference
Neoplasms	0.5 mg or 1.0 mg for 56 weeks	*n* = 818	*n* = 0	*n* = 14	[110]
EAC-confirmed neoplasms	1.0 mg for 56 weeks	*n* = 813	N/A	*n* = 15	[111]
EAC-confirmed neoplasms	0.5 or 1.0 mg for 30 weeks	*n* = 397	*n* = 1	*n* = 5	[97]
Neoplasms	0.5 or 1.0 mg for 104 weeks	*n* = 136	*n* = 70	*n* = 66	[113]
Neoplasms	0.5 or 1.0 mg for 24 weeks	*n* = 601	N/A	*n* = 6	[114]
Neoplasms	1.0 mg for 52 weeks	*n* = 367	N/A	*n* = 3	[115]
Neoplasms	1.0 mg for 37 weeks	*n* = 302	*n* = 5	*n* = 4	[116]
Neoplasms	1.0 mg for 30 weeks	*n* = 577	N/A	*n* = 9	[117]
Neoplasms	1.0 mg for 52 weeks	*n* = 874	N/A	*n* = 11	[118]
Neoplasms	0.5 or 1.0 mg for 63 weeks	*n* = 595	N/A	*n* = 44	[121]
Neoplasms	0.5 or 1.0 mg for 30 weeks	*n* = 578	N/A	*n* = 23	[122]
Thyroid	1.0 mg for 104 weeks	*n* = 1648	*n* = 4	*n* = 1	[127]
Thyroid	1.0 mg for 56 weeks	*n* = 818	*n* = 0	*n* = 1	[110]
Bladder	1.0 mg for 30 weeks	*n* = 205	N/A	*n* = 1	[120]
Bladder	1.0 mg for 56 weeks	*n* = 818	*n* = 0	*n* = 1	[110]
Pancreatic	0.5 mg for 30 weeks	*n* = 722	N/A	*n* = 1	[112]
Pancreatic	1.0 mg for 30 weeks	*n* = 397	N/A	*n* = 1	[97]
Colorectal	0.5 mg for 30 weeks	*n* = 397	*n* = 1	*n* = 1	[97]
Colorectal	0.5 or 1.0 mg for 63 weeks	*n* = 595	N/A	*n* = 18	[121]

Semaglutide is associated with increased neoplasm and tumorigenesis, specifically in the thyroid, bladder, colorectal, and pancreas. Doses of 0.5–1.0 mg yielded 1–155 cases of cancer development in the treatment period of 30–104 weeks. Lower cases were reported in the thyroid, bladder, colorectal, and pancreas compared to a treatment period of 104 weeks with 0.5 mg of semaglutide, which yielded 155 cases of neoplasm. On the contrary, for the same treatment period but at a drug concentration of 1.0 mg, there was only 1 case of thyroid cancer (Table 4).

Semaglutide, among other antidiabetic drugs, has shown an association with cancer as it alters the rates of tumorigenesis and proliferation. This GLP-R1 agonist seems to increase oncogenesis in multiple tissues, including the thyroid, bladder, pancreatic, and colorectal [97, 110, 121].

Pharmaceutical companies have issued a warning about the use of formulations of semaglutide with those who have thyroid cancer or are at risk of developing it [128]. Despite some studies showing cases of cancer development, the numbers reported are as minimal as one case. There is no conclusive evidence that semaglutide induced cancer development in tissue, which may imply that the development of cancer may pertain to other causes rather than semaglutide application [129]. On the contrary, several authors report mitigation of cancer proliferation using the same dose of 0.5–1.0 mg semaglutide ingested by diabetic patients and for similar periods. The increase in semaglutide is gradual and can be altered to scale up every 30 days when ingested orally. This accumulation may aid semaglutide’s action against cancer cells [130]. There is a pool of research on the effect of liraglutide and exendin on cancer. However, there is a lack of research data on the impact of semaglutide *in vitro* and *in vivo*, which limits its efficiency in tumor therapy. Further research must be conducted to understand the effects of semaglutide on cancer as it belongs to the GLP-1R family along with liraglutide and exendin and may provide similar results.

## Conclusions

Semaglutide, like many other GLP-1R agonists, is used in diabetes and obesity to decrease glucose levels and manage body weight, which plays a role in tumorigenesis. The insufficient *in vivo* studies on semaglutide and limited *in vitro* research raise concerns about the imperative for more comprehensive investigations into its effects. Specifically, there is a need to elucidate a descriptive mechanism through which semaglutide reduces diabetes and obesity and potentially influences cancer. Although there is a lack of direct studies on semaglutide, the observed actions align with those of other GLP-1R agonists, indicating a potential impact on cancer. There is a notable increase in thyroid cancer with the use of GLP-1R agonists, including semaglutide. The use of GLP-1R agonists increases calcitonin gene-related peptide (CGRP) in thyroid cancer. Overexpression of pS6, mTOR activation, calcitonin, and C-cell hyperplasia reported in *in vivo* suggest the increased proliferation and tumorigenesis [75, 87]. Despite the cancer cases recorded with the use of semaglutide, other factors may have contributed to cancer development with no association with semaglutide [129]. However, whether Semaglutide’s effect is mitigating or exacerbating remains unclear, emphasizing the necessity for further research on the outcome of GLP-1R agonists on cancer, specifically thyroid cancer.

## Electronic supplementary material

Below is the link to the electronic supplementary material.

Supplementary Material 1

## Data Availability

No datasets were generated or analyzed during the current study.

## Abbreviations

Akt: Protein kinase B
BMI: Body mass index
cAMP: Cyclic adenosine monophosphate
CGRP: Calcitonin gene-related peptide
DM: Diabetes mellitus
DPP: 4-dipeptidyl peptidase-4
GLP: 1-glucagon-like peptide-1
GLP: 1R-glucagon-like peptide-1 receptor
HbA1c: Glycated hemoglobin
IGF: Insulin-like growth factor
MAPK: Mitogen-activated protein kinase
mTOR: Mammalian target of rapamycin
NF: κB-nuclear factor κB
PI3K: Phosphoinositide 3-kinase
PKA: cAMP-dependent protein kinase
SGLT: 2-sodium-glucosecotransporter-2
T2DM: Type 2 diabetes mellitus

## References

[1] Iijima, T., Shibuya, M., Ito, Y., & Terauchi, Y. (2023). Effects of switching from liraglutide to semaglutide or dulaglutide in patients with type 2 diabetes: A randomized controlled trial. Nature Reviews Disease Primers , *14*. 10.1111/jdi.14000. 10.1111/jdi.14000 PMC10204181 36871272

[2] Kadowaki, T., Isendahl, J., Khalid, U., Lee, S. Y., Nishida, T., Ogawa, W., Tobe, K., Yamauchi, T., & Lim, S. (2022). Semaglutide once a week in adults with overweight or obesity, with or without type 2 diabetes in an east Asian population (step 6): A randomised, double-blind, double-dummy, placebo-controlled, phase 3a trial. *Lancet Diabetes Endocrinol,**10*, 193–206. 10.1016/S2213-8587(22)00008-0 35131037 10.1016/S2213-8587(22)00008-0

[3] Pérez Rodrigo, C. (2013). Current mapping of obesity. *Nutricion Hospitalaria*, *28*(Suppl 5), 21–31. 10.3305/NH.2013.28.SUP5.6915. 24010741 10.3305/nh.2013.28.sup5.6915

[4] Chooi, Y. C., Ding, C., & Magkos, F. (2019). The epidemiology of obesity. *Metabolism*, *92*. 10.1016/j.metabol.2018.09.005. 10.1016/j.metabol.2018.09.005 30253139

[5] Männistö, S., Kontto, J., Kataja-Tuomola, M., Albanes, D., & Virtamo, J. (2010). High processed meat consumption is a risk factor of type 2 diabetes in the alpha-tocopherol, beta-carotene cancer prevention study. *British Journal of Nutrition*, *103*. 10.1017/S0007114510000073. 10.1017/S0007114510000073 PMC3496924 20187985

[6] Ong, K. L., Stafford, L. K., McLaughlin, S. A., Boyko, E. J., Vollset, S. E., Smith, A. E., Dalton, B. E., Duprey, J., Cruz, J. A., Hagins, H., et al. (2023). Global, Regional, and National Burden of Diabetes from 1990 to 2021, with projections of prevalence to 2050: A systematic analysis for the global burden of Disease Study 2021. *The Lancet*, *402*. 10.1016/S0140-6736(23)01301-6. 10.1016/S0140-6736(23)01301-6 PMC10364581 37356446

[7] Buzzetti, R., Maddaloni, E., Gaglia, J., Leslie, R. D., Wong, F. S., & Boehm, B. O. (2022). Adult-onset autoimmune diabetes. Nature Reviews Clinical Oncology, *8*. 10.1038/s41572-022-00390-6. 10.1038/s41572-022-00390-6 36138034

[8] Scully, T., Ettela, A., LeRoith, D., Gallagher, E. J., & Obesity (2021). Type 2 diabetes, and cancer risk. *Frontiers in Oncology* 10. 10.3389/fonc.2020.615375 PMC7884814 33604295

[9] Wirth, A., Wabitsch, M., & Hauner, H. (2014). The prevention and treatment of obesity. *Dtsch Arztebl Int*, *111*. 10.3238/arztebl.2014.0705. 10.3238/arztebl.2014.0705 PMC4233761 25385482

[10] Avgerinos, K. I., Spyrou, N., Mantzoros, C. S., & Dalamaga, M. (2019). Obesity and cancer risk: Emerging biological mechanisms and perspectives. *Metabolism,**92*, 121–135. 10.1016/J.METABOL.2018.11.001 30445141 10.1016/j.metabol.2018.11.001

[11] Preuss, H. G., Bagchi, M., Bagchi, D., & Kaats, G. R. (2010). Obesity and cancer. *The Oncologist,**15*, 197–204. 10.1634/THEONCOLOGIST.2009-0285

[12] Kim, D. S., Scherer, P. E., & Obesity. (2021). Diabetes, and increased cancer progression. *Diabetes Metab J,* 45. 10.4093/dmj.2021.0077 PMC8640143 34847640

[13] López-Suárez, A. (2019). Burden of cancer attributable to obesity, type 2 diabetes and associated risk factors. Metabolism 92. 10.1016/j.metabol.2018.10.013 30412695

[14] Buhrmann, C., Shayan, P., Brockmueller, A., & Shakibaei, M. (2020). Resveratrol suppresses cross-talk between colorectal cancer cells and stromal cells in multicellular tumor microenvironment: A bridge between in vitro and in vivo tumor microenvironment study. *Molecules*, *25*. 10.3390/molecules25184292. 10.3390/molecules25184292 PMC7570736 32962102

[15] Coussens, L. M., & Werb, Z. (2002). Inflammation and cancer. *Nature*, *420*, 860–867. 10.1038/NATURE01322. 10.1038/nature01322 PMC2803035 12490959

[16] Islami, F., Goding Sauer, A., Gapstur, S. M., & Jemal, A. (2019). Proportion of cancer cases attributable to excess body weight by US state, 2011–2015. *JAMA Oncology*, *5*. 10.1001/jamaoncol.2018.5639. 10.1001/jamaoncol.2018.5639 PMC6521676 30589925

[17] Pati, S., Irfan, W., Jameel, A., Ahmed, S., & Shahid, R. K. (2023). Obesity and cancer: A current overview of epidemiology, pathogenesis, outcomes, and management. *Cancers (Basel)* 15. 10.3390/cancers15020485 PMC9857053 36672434

[18] De Pergola, G., & Silvestris, F. (2013). Obesity as a major risk factor for cancer. J Obes 2013. 10.1155/2013/291546 PMC3773450 24073332

[19] Bhardwaj, P., Iyengar, N. M., Zahid, H., Carter, K. M., Byun, D. J., Choi, M. H., Sun, Q., Savenkov, O., Louka, C., Liu, C., et al. (2023). Obesity promotes breast epithelium DNA damage in women carrying a germline mutation in BRCA1 or BRCA2. *Science Translational Medicine*, *15*. 10.1126/scitranslmed.ade1857. 10.1126/scitranslmed.ade1857 PMC10557057 36812344

[20] Harreiter, J., Roden, M., Diabetes, & Mellitus—Definition (2019). Classification, diagnosis, screening and prevention (Update 2019). *Wien Klin Wochenschr*, *131*. 10.1007/s00508-019-1450-4. 10.1007/s00508-019-1450-4 30980151

[21] Nolen, L. (2022). The effect of glucose on rapid cancer cell proliferation & waste. *Oncology Times*, *44*. 10.1097/01.cot.0000892616.48647.47.

[22] Khajah, M. A., Khushaish, S., & Luqmani, Y. A. (2022). Glucose deprivation reduces proliferation and motility, and enhances the anti-proliferative effects of paclitaxel and doxorubicin in breast cell lines in Vitro. *PLoS One*, *17*. 10.1371/journal.pone.0272449. 10.1371/journal.pone.0272449 PMC9345370 35917304

[23] Lin, C. Y., Lee, C. H., Huang, C. C., Lee, S. T., Guo, H. R., & Su, S. (2015). Bin impact of high glucose on metastasis of colon cancer cells. *World Journal of Gastroenterology*, *21*. 10.3748/wjg.v21.i7.2047. 10.3748/wjg.v21.i7.2047 PMC4326139 25717237

[24] Derr, R. L., Ye, X., Islas, M. U., Desideri, S., Saudek, C. D., & Grossman, S. A. (2009). Association between hyperglycemia and survival in patients with newly diagnosed glioblastoma. *Journal of Clinical Oncology*, *27*. 10.1200/JCO.2008.19.1098. 10.1200/JCO.2008.19.1098 PMC2667812 19139429

[25] Rahn, S., Zimmermann, V., Viol, F., Knaack, H., Stemmer, K., Peters, L., Lenk, L., Ungefroren, H., Saur, D., Schäfer, H., et al. (2018). Diabetes as risk factor for pancreatic cancer: Hyperglycemia promotes epithelial-mesenchymal-transition and stem cell properties in pancreatic ductal epithelial cells. *Cancer Letters*, *415*. 10.1016/j.canlet.2017.12.004. 10.1016/j.canlet.2017.12.004 29222037

[26] Qiang, J. K., Lipscombe, L. L., & Lega, I. C. (2020). Association between diabetes, obesity, aging, and cancer: Review of recent literature. *Translation Cancer Research* 9. 10.21037/tcr.2020.03.14 PMC8797908 35117936

[27] Granja, S., Pinheiro, C., Reis, R., Martinho, O., & Baltazar, F. (2015). Glucose addiction in cancer therapy: Advances and drawbacks. *Current Drug Metabolism*, *16*. 10.2174/1389200216666150602145145. 10.2174/1389200216666150602145145 26504932

[28] Brockmueller, A., Sameri, S., Liskova, A., Zhai, K., Varghese, E., Samuel, S. M., Büsselberg, D., Kubatka, P., & Shakibaei, M. (2021). Resveratrol’s anti-cancer effects through the modulation of tumor glucose metabolism. *Cancers (Basel),**13*, 1–35. 10.3390/CANCERS13020188 10.3390/cancers13020188 PMC7825813 33430318

[29] Samec, M., Liskova, A., Koklesova, L., Samuel, S. M., Zhai, K., Buhrmann, C., Varghese, E., Abotaleb, M., Qaradakhi, T., Zulli, A. (2020). Flavonoids against the Warburg phenotype—concepts of predictive, preventive and personalised medicine to cut the Gordian knot of cancer cell metabolism. *EPMA Journal* 11. 10.1007/s13167-020-00217-y PMC7429635 32843908

[30] Klil-Drori, A. J., Azoulay, L., Pollak, M. N., & Cancer. (2017). Obesity, diabetes, and antidiabetic drugs: Is the fog clearing? *Nature Reviews Clinical Oncology,**14*, 85–99. 10.1038/NRCLINONC.2016.120 27502359 10.1038/nrclinonc.2016.120

[31] Slawinski, C. G. V., Barriuso, J., Guo, H., & Renehan, A. G. (2020). Obesity and cancer treatment outcomes: Interpreting the complex evidence. *Clinical Oncology (Royal College of Radiologists),**32*, 591–608. 10.1016/J.CLON.2020.05.004 10.1016/j.clon.2020.05.004 32595101

[32] Vucenik, I., Stains, J. P., Obesity, & Risk, C. (2012). Evidence, mechanisms, and recommendations. *Annals of the New York Academy of Sciences*, *1271*, 37–43. 10.1111/J.1749-6632.2012.06750.X. 23050962 10.1111/j.1749-6632.2012.06750.x PMC3476838

[33] Rowbottom, L., Stinson, J., McDonald, R., Emmenegger, U., Cheng, S., Lowe, J., Giotis, A., Cheon, P., Chow, R., Pasetka, M., et al. (2015). Retrospective review of the incidence of monitoring blood glucose levels in patients receiving corticosteroids with systemic anticancer therapy. *Ann Palliat Med*, *4*, 70–77. 10.3978/J.ISSN.2224-5820.2015.04.07. 25971294 10.3978/j.issn.2224-5820.2015.04.07

[34] Shahid, R. K., Ahmed, S., Le, D., & Yadav, S. (2021). Diabetes and cancer: Risk, challenges, management and outcomes. *Cancers (Basel)* 13. 10.3390/cancers13225735 PMC8616213 34830886

[35] Zhu, L., Zhou, J., Pan, Y., Lv, J., Liu, Y., Yu, S., & Zhang, Y. (2019). Glucagon-like peptide-1 receptor expression and its functions are regulated by androgen. *Biomedicine and Pharmacotherapy*, *120*. 10.1016/j.biopha.2019.109555. 10.1016/j.biopha.2019.109555 31669915

[36] Drucker, D. J. (2022). GLP-1 physiology informs the pharmacotherapy of obesity. *Molecular Metabolism* 57. 10.1016/j.molmet.2021.101351 PMC8859548 34626851

[37] Nomiyama, T., & Yanase, T. (2016). GLP-1 receptor agonist as treatment for cancer as well as diabetes: Beyond blood glucose control. *Expert Review Endocrinology Metabolism*, 1–8. 10.1080/17446651.2016.1191349. 10.1080/17446651.2016.1191349 30058925

[38] Koehler, J. A., Kain, T., Drucker, D. J., & Glucagon-Like. (2011). Peptide-1 receptor activation inhibits growth and augments apoptosis in murine CT26 colon cancer cells. *Endocrinology,**152*, 3362–3372. 10.1210/en.2011-1201 21771884 10.1210/en.2011-1201

[39] Wang, L., Wang, W., Kaelber, D. C., Xu, R., & Berger, N. A. (2024). GLP-1 receptor agonists and colorectal cancer risk in drug-naive patients with type 2 diabetes, with and without overweight/obesity. *JAMA Oncology,**10*, 256. 10.1001/jamaoncol.2023.5573 38060218 10.1001/jamaoncol.2023.5573 PMC10704339

[40] Skriver, C., Friis, S., Knudsen, L. B., Catarig, A. M., Clark, A. J., Dehlendorff, C., & Mørch, L. S. (2023). Potential preventive properties of GLP-1 receptor agonists against prostate cancer: A nationwide cohort study. *Diabetologia,**66*, 2007–2016. 10.1007/s00125-023-05972-x 37532786 10.1007/s00125-023-05972-x

[41] Handbook, H. (2016).

[42] Johnson, L. R., Barret, K. E., Gishan, F. K., Merchant, J. L., Said, H. M., & Wood, J. D. (2006). *Physiology of the gastrointestinal tract*; ; Vol. 1–2.

[43] Rowlands, J., Heng, J., Newsholme, P., & Carlessi, R. (2018). Pleiotropic effects of GLP-1 and analogs on cell signaling, metabolism, and function. *Front Endocrinol (Lausanne)* 9. 10.3389/fendo.2018.00672 PMC6266510 30532733

[44] Carlessi, R., Chen, Y., Rowlands, J., Cruzat, V. F., Keane, K. N., Egan, L., Mamotte, C., Stokes, R., Gunton, J. E., Bittencourt, P. I. H., De, et al. (2017). GLP-1 receptor signalling promotes β-cell glucose metabolism via MTOR-dependent HIF-1α activation. *Scientific Reports*, *7*. 10.1038/S41598-017-02838-2. 10.1038/s41598-017-02838-2 PMC5454020 28572610

[45] Detka, J., & Głombik, K. (2021). Insights into a possible role of glucagon-like Peptide-1 receptor agonists in the treatment of depression. *Pharmacological Reports,**73*, 1020. 10.1007/S43440-021-00274-8 34003475 10.1007/s43440-021-00274-8 PMC8413152

[46] Koole, C., Savage, E. E., Christopoulos, A., Miller, L. J., Sexton, P. M., Wootten, D., & Minireview. (2013). Signal bias, allosterism, and polymorphic variation at the GLP-1R: Implications for drug discovery. *Molecular Endocrinology,**27*, 1234–1244. 10.1210/ME.2013-1116 23864649 10.1210/me.2013-1116 PMC3725346

[47] Sato, T., Shimizu, T., Fujita, H., Imai, Y., Drucker, D. J., Seino, Y., & Yamada, Y. (2020). GLP-1 receptor signaling differentially modifies the outcomes of sterile vs viral pulmonary inflammation in male mice. *Endocrinology*, *161*. 10.1210/ENDOCR/BQAA201. 10.1210/endocr/bqaa201 PMC7678414 33125041

[48] Wang, W., Mei, A., Qian, H., Li, D., Xu, H., Chen, J., Yang, H., Min, X., Li, C., Cheng, L., et al. (2023). The role of glucagon-like peptide-1 receptor agonists in chronic obstructive pulmonary disease. *Int J Chron Obstruct Pulmon Dis,**18*, 129. 10.2147/COPD.S393323 36815056 10.2147/COPD.S393323 PMC9939668

[49] Broide, E., Bloch, O., Ben-Yehudah, G., Cantrell, D., Shirin, H., & Rapoport, M. J. (2013). GLP-1 receptor is expressed in human stomach mucosa: Analysis of its cellular association and distribution within gastric glands. *Journal of Histochemistry and Cytochemistry,**61*, 649–658. 10.1369/0022155413497586 23803499 10.1369/0022155413497586 PMC3753891

[50] Holst, J. J., Andersen, D. B., & Grunddal, K. V. (2022). Actions of glucagon-like peptide-1 receptor ligands in the gut. *British Journal of Pharmacology,**179*, 727–742. 10.1111/BPH.15611 34235727 10.1111/bph.15611 PMC8820219

[51] Zhao, X., Wang, M., Wen, Z., Lu, Z., Cui, L., Fu, C., Xue, H., Liu, Y., & Zhang, Y. (2021). GLP-1 receptor agonists: Beyond their pancreatic effects. *Front Endocrinol (Lausanne)* 12. 10.3389/fendo.2021.721135 PMC8419463 34497589

[52] Wang, X. C., Gusdon, A. M., Liu, H., & Qu, S. (2014). Effects of glucagon-like peptide-1 receptor agonists on non-alcoholic fatty liver disease and inflammation. *World Journal of Gastroenterology* 20. 10.3748/wjg.v20.i40.14821 PMC4209545 25356042

[53] Yabut, J. M., & Drucker, D. J. (2023). Glucagon-like peptide-1 receptor-based therapeutics for metabolic liver disease. *Endocrine Reviews,**44*, 14–32. 10.1210/ENDREV/BNAC018 35907261 10.1210/endrev/bnac018

[54] Wong, C., Lee, M. H., Yaow, C. Y. L., Chin, Y. H., Goh, X. L., Ng, C. H., Lim, A. Y. L., Muthiah, M. D., & Khoo, C. M. (2021). Glucagon-like peptide-1 receptor agonists for non-alcoholic fatty liver disease in type 2 diabetes: A meta-analysis. *Front Endocrinol (Lausanne) 12*, 10.3389/FENDO.2021.609110. 10.3389/fendo.2021.609110 PMC8063104 33897616

[55] Liu, X., Patel, K. P., & Zheng, H. (2021). Role of renal sympathetic nerves in GLP-1 (glucagon-like peptide-1) receptor agonist exendin-4-mediated diuresis and natriuresis in diet-induced obese rats. *Journal of American Heart Association*, *10*. 10.1161/JAHA.121.022542. 10.1161/JAHA.121.022542 PMC8751817 34713714

[56] Górriz, J. L., Soler, M. J., Navarro-González, J. F., García-Carro, C., Puchades, M. J., D’marco, L., Castelao, A. M., Fernández-Fernández, B., Ortiz, A., & Górriz-Zambrano, C. (2020). GLP-1 receptor agonists and diabetic kidney disease: A call of attention to nephrologists. *Journal Clininical Medicine,* 9. 10.3390/jcm9040947 PMC7231090 32235471

[57] Granata, A., Maccarrone, R., Anzaldi, M., Leonardi, G., Pesce, F., Amico, F., Gesualdo, L., & Corrao, S. (2022). GLP-1 receptor agonists and renal outcomes in patients with diabetes mellitus type 2 and diabetic kidney disease: State of the art. *Clinical Kidney Journal,* 15. 10.1093/ckj/sfac069 PMC9394722 36003669

[58] Baggio, L. L., Yusta, B., Mulvihill, E. E., Cao, X., Streutker, C. J., Butany, J., Cappola, T. P., Margulies, K. B., & Drucker, D. J. (2018). GLP-1 receptor expression within the human heart. *Endocrinology,**159*,. 10.1210/en.2018-00004 10.1210/en.2018-00004 PMC5939638 29444223

[59] Del Olmo-Garcia, M. I., & Merino-Torres, J. F. (2018). GLP-1 receptor agonists and cardiovascular disease in patients with type 2 diabetes. *Journal Diabetes Research 2018*, 10.1155/2018/4020492. 10.1155/2018/4020492 PMC5902002 29805980

[60] Hou, Y., Ernst, S. A., Heidenreich, K., & Williams, J. A. (2016). Glucagon-like peptide-1 receptor is present in pancreatic acinar cells and regulates amylase secretion through CAMP. *American Journal Of Physiology. Gastrointestinal And Liver Physiology,**310*,. 10.1152/ajpgi.00293.2015 10.1152/ajpgi.00293.2015 PMC4698438 26542397

[61] Doyle, M. E., & Egan, J. M. (2007). Mechanisms of action of glucagon-like peptide 1 in the pancreas. *Pharmacology & Therapeutics,* 113. 10.1016/j.pharmthera.2006.11.007 PMC1934514 17306374

[62] Liu, J., & Pang, Z. P. (2016). Glucagon-like peptide-1 drives energy metabolism on the synaptic highway. *The Febs Journal,**283*, 4413–4423. 10.1111/FEBS.13785 27315220 10.1111/febs.13785

[63] Jessen, L., Smith, E. P., Ulrich-Lai, Y., Herman, J. P., Seeley, R. J., Sandoval, D., & D’Alessio, D. (2017). Central nervous system GLP-1 receptors regulate islet hormone secretion and glucose homeostasis in male rats. *Endocrinology,**158*,. 10.1210/en.2016-1826 10.1210/en.2016-1826 PMC5505222 28430981

[64] Gier, B., Butler, P. C., Lai, C. K., Kirakossian, D., DeNicola, M. M., & Yeh, M. W. (2012). Glucagon like peptide-1 receptor expression in the human thyroid gland. *Journal of Clinical Endocrinology and Metabolism,**97*,. 10.1210/jc.2011-2407 10.1210/jc.2011-2407 PMC3412261 22031513

[65] Bezin, J., Gouverneur, A., Penichon, M., Mathieu, C., Garrel, R., Hillaire-Buys, D., Pariente, A., & Faillie, J. L. (2023). GLP-1 receptor agonists and the risk of thyroid cancer. *Diabetes Care,**46*, 384–390. 10.2337/DC22-1148 36356111 10.2337/dc22-1148

[66] Shigeoka, T., Nomiyama, T., Kawanami, T., Hamaguchi, Y., Horikawa, T., Tanaka, T., Irie, S., Motonaga, R., Hamanoue, N., Tanabe, M., et al. (2020). Activation of overexpressed glucagon-like peptide-1 receptor attenuates prostate cancer growth by inhibiting cell cycle progression. *Journal Diabetes Investigation,**11*,. 10.1111/jdi.13247 10.1111/jdi.13247 PMC7477521 32146725

[67] Liu, Z., Duan, X., & Yuan, M. (2022). Yu, J.; Hu, X.; Han, X.; Lan, L.; Liu, B. wei; Wang, Y.; Qin, J. fang glucagon-like peptide-1 receptor activation by liraglutide promotes breast cancer through NOX4/ROS/VEGF pathway. *Life Science 294*, 10.1016/j.lfs.2022.120370. 10.1016/j.lfs.2022.120370 35124000

[68] Zhao, H. J., Jiang, X., Hu, L. J., Yang, L., Deng, L. D., Wang, Y. P., & Ren, Z. P. (2020). Activation of GLP-1 receptor enhances the chemosensitivity of pancreatic cancer cells. *Journal of Molecular Endocrinology*, *64*. 10.1530/JME-19-0186. 10.1530/JME-19-0186 31855560

[69] Collins, L., & Costello, R. A. (2023). Glucagon-like peptide-1 receptor agonists. *StatPearls*. 31855395

[70] Rosenstock, J., Nino, A., Soffer, J., Erskine, L., Acusta, A., Dole, J., Carr, M. C., Mallory, J., & Home, P. (2020). Impact of a weekly glucagon-like peptide 1 receptor agonist, albiglutide, on glycemic control and on reducing prandial insulin use in type 2 diabetes inadequately controlled on multiple insulin therapy: A randomized trial. *Diabetes Care,**43*,. 10.2337/dc19-2316 10.2337/dc19-2316 PMC7510023 32694215

[71] Latif, W., Lambrinos, K. J., & Rodriguez, R. (2023). Compare and contrast the glucagon-like peptide-1 receptor agonists (GLP1RAs). *StatPearls*. 34283517

[72] Nauck, M. A., Quast, D. R., Wefers, J., & Meier, J. J. (2021). GLP-1 receptor agonists in the treatment of type 2 diabetes – state-of-the-art. *Mol Metab*, *46*, 101102. 10.1016/j.molmet.2020.101102. 33068776 10.1016/j.molmet.2020.101102 PMC8085572

[73] Meier, J. J. (2012). GLP-1 receptor agonists for individualized treatment of type 2 diabetes mellitus. *Nature Reviews. Endocrinology,* 8. 10.1038/nrendo.2012.140 22945360

[74] Tong, G., Peng, T., Chen, Y., Sha, L., Dai, H., Xiang, Y., Zou, Z., He, H., & Wang, S. (2022). Effects of GLP-1 receptor agonists on biological behavior of colorectal cancer cells by regulating PI3K/AKT/MTOR signaling pathway. *Frontiers In Pharmacology,**13*,. 10.3389/fphar.2022.901559 10.3389/fphar.2022.901559 PMC9399678 36034798

[75] Lamari, Y., Boissard, C., Moukhtar, M. S., Jullienne, A., Rosselin, G., & Garel, J. M. (1996). Expression of glucagon-like peptide 1 receptor in a murine C cell line: Regulation of calcitonin gene by glucagon-like peptide 1. *Febs Letters,**393*,. 10.1016/0014-5793(96)00895-2 10.1016/0014-5793(96)00895-2 8814299

[76] Iwaya, C., Nomiyama, T., Komatsu, S., Kawanami, T., Tsutsumi, Y., Hamaguchi, Y., Horikawa, T., Yoshinaga, Y., Yamashita, S., Tanaka, T., et al. (2017). Exendin-4, a glucagonlike peptide-1 receptor agonist, attenuates breast cancer growth by inhibiting NF-KB activation. *Endocrinology,**158*,. 10.1210/en.2017-00461 10.1210/en.2017-00461 29045658

[77] Tanaka, Y., Iwaya, C., Kawanami, T., Hamaguchi, Y., Horikawa, T., Shigeoka, T., Yanase, T., Kawanami, D., & Nomiyama, T. (2022). Combined treatment with glucagon-like peptide-1 receptor agonist Exendin-4 and metformin attenuates breast cancer growth. *Diabetology International,**13*,. 10.1007/s13340-021-00560-z 10.1007/s13340-021-00560-z PMC9174406 35693999

[78] Fidan-Yaylalı, G., Dodurga, Y., Seçme, M., & Elmas, L. (2016). Antidiabetic exendin-4 activates apoptotic pathway and inhibits growth of breast cancer cells. *Tumor Biology,**37*,. 10.1007/s13277-015-4104-9 10.1007/s13277-015-4104-9 26399993

[79] Ligumsky, H., Wolf, I., Israeli, S., Haimsohn, M., Ferber, S., Karasik, A., Kaufman, B., & Rubinek, T. (2012). The peptide-hormone glucagon-like peptide-1 activates CAMP and inhibits growth of breast cancer cells. *Breast Cancer Research And Treatment,**132*,. 10.1007/s10549-011-1585-0 10.1007/s10549-011-1585-0 21638053

[80] Nomiyama, T., Kawanami, T., Irie, S., Hamaguchi, Y., Terawaki, Y., Murase, K., Tsutsumi, Y., Nagaishi, R., Tanabe, M., Morinaga, H., et al. (2014). Exendin-4, a GLP-1 receptor agonist, attenuates prostate cancer growth. *Diabetes,**63*,. 10.2337/db13-1169 10.2337/db13-1169 24879833

[81] Eftekhari, S., Montazeri, H., & Tarighi, P. (2020). Synergistic anti-tumor effects of liraglutide, a glucagon-like peptide-1 receptor agonist, along with docetaxel on LNCaP prostate cancer cell line. *European Journal of Pharmacology,**878*,. 10.1016/j.ejphar.2020.173102 10.1016/j.ejphar.2020.173102 32283060

[82] Li, X. N., Bu, H. M., Ma, X. H., Lu, S., Zhao, S., Cui, Y. L., & Sun, J. (2017). Glucagon-like peptide-1 analogues inhibit proliferation and increase apoptosis of human prostate cancer cells in vitro. *Experimental and Clinical Endocrinology and Diabetes,**125*,. 10.1055/s-0042-112368 10.1055/s-0042-112368 28008585

[83] Zhao, H., Wang, L., Wei, R., Xiu, D., Tao, M., Ke, J., Liu, Y., Yang, J., & Hong, T. (2014). Activation of glucagon-like peptide-1 receptor inhibits tumourigenicity and metastasis of human pancreatic cancer cells via PI3K/Akt pathway. *Diabetes, Obesity & Metabolism,**16*,. 10.1111/dom.12291 10.1111/dom.12291 24641303

[84] Koehler, J. A., Kain, T., & Drucker, D. J. (2011). Glucagon-like peptide-1 receptor activation inhibits growth and augments apoptosis in murine CT26 colon cancer cells. *Endocrinology,**152*,. 10.1210/en.2011-1201 10.1210/en.2011-1201 21771884

[85] He, W., Yu, S., Wang, L., He, M., Cao, X., Li, Y., & Xiao, H. (2016). Exendin-4 inhibits growth and augments apoptosis of ovarian cancer cells. *Molecular And Cellular Endocrinology,**436*,. 10.1016/j.mce.2016.07.032 10.1016/j.mce.2016.07.032 27496641

[86] Verbeek, H. H. G., de Groot, J. W. B., Sluiter, W. J., Muller Kobold, A. C., van den Heuvel, E. R., Plukker, J. T. M., & Links, T. P. (2020). Calcitonin testing for detection of medullary thyroid cancer in people with thyroid nodules. Cochrane Database of Systematic Reviews 2020. 10.1002/14651858.CD010159.pub2 PMC7075519 32176812

[87] Madsen, L. W., Knauf, J. A., Gotfredsen, C., Pilling, A., Sjögren, I., Andersen, S., Andersen, L., De Boer, A. S., Manova, K., Barlas, A., et al. (2012). GLP-1 receptor agonists and the thyroid: C-cell effects in mice are mediated via the GLP-1 receptor and not associated with RET activation. *Endocrinology,**153*,. 10.1210/en.2011-1864 10.1210/en.2011-1864 PMC3281535 22234463

[88] Tsutsumi, Y., Nomiyama, T., Kawanami, T., Hamaguchi, Y., Terawaki, Y., Tanaka, T., Murase, K., Motonaga, R., Tanabe, M., Yanase, T., et al. (2015). Combined treatment with exendin-4 and metformin attenuates prostate cancer growth. *PLoS One,**10*,. 10.1371/journal.pone.0139709 10.1371/journal.pone.0139709 PMC4595004 26439622

[89] Koehler, J. A., Baggio, L. L., Yusta, B., Longuet, C., Rowland, K. J., Cao, X., Holland, D., Brubaker, P. L., & Drucker, D. J. (2015). GLP-1R agonists promote normal and neoplastic intestinal growth through mechanisms requiring Fgf7. *Cell Metab,**21*,. 10.1016/j.cmet.2015.02.005 10.1016/j.cmet.2015.02.005 25738454

[90] Kojima, M., Takahashi, H., Kuwashiro, T., Tanaka, K., Mori, H., Ozaki, I., Kitajima, Y., Matsuda, Y., Ashida, K., Eguchi, Y., et al. (2020). Glucagon-like peptide-1 receptor agonist prevented the progression of hepatocellular carcinoma in a mouse model of nonalcoholic steatohepatitis. *International Journal Of Molecular Sciences,**21*,. 10.3390/ijms21165722 10.3390/ijms21165722 PMC7460814 32785012

[91] Lu, R., Yang, J., Wei, R., Ke, J., Tian, Q., Yu, F., Liu, J., Zhang, J., & Hong, T. (2018). Synergistic anti-tumor effects of liraglutide with metformin on pancreatic cancer cells. *PLoS One,**13*,. 10.1371/journal.pone.0198938 10.1371/journal.pone.0198938 PMC5999272 29897998

[92] Zhao, W., Zhang, X., Zhou, Z., Sun, B., Gu, W., Liu, J., & Zhang, H. (2018). Liraglutide inhibits the proliferation and promotes the apoptosis of MCF-7 human breast cancer cells through downregulation of microRNA-27a expression. *Molecular Medicine Reports,**17*,. 10.3892/mmr.2018.8475 10.3892/mmr.2018.8475 PMC5865986 29393459

[93] Iwaya, C., Nomiyama, T., Komatsu, S., Kawanami, T., Tsutsumi, Y., Hamaguchi, Y., Horikawa, T., Yoshinaga, Y., Yamashita, S., Tanaka, T., et al. (2017). Exendin-4, a glucagonlike peptide-1 receptor agonist, attenuates breast cancer growth by inhibiting NF-ΚB activation. *Endocrinology,**158*, 4218–4232. 10.1210/EN.2017-00461 29045658 10.1210/en.2017-00461

[94] Tanaka, Y., Iwaya, C., Kawanami, T., Hamaguchi, Y., Horikawa, T., Shigeoka, T., Yanase, T., Kawanami, D., & Nomiyama, T. (2022). Combined treatment with glucagon-like peptide-1 receptor agonist exendin-4 and metformin attenuates breast cancer growth. *Diabetology International,**13*, 480–492. 10.1007/s13340-021-00560-z 35693999 10.1007/s13340-021-00560-z PMC9174406

[95] Tsutsumi, Y., Nomiyama, T., Kawanami, T., Hamaguchi, Y., Terawaki, Y., Tanaka, T., Murase, K., Motonaga, R., Tanabe, M., & Yanase, T. (2015). Combined treatment with exendin-4 and metformin attenuates prostate cancer growth. *PLoS One,**10*, e0139709. 10.1371/journal.pone.0139709 26439622 10.1371/journal.pone.0139709 PMC4595004

[96] Dhillon, S., & Semaglutide (2018). First global approval. *Drugs 78*, 10.1007/s40265-018-0871-0. 10.1007/s40265-018-0871-0 29363040

[97] Rodbard, H. W., Lingvay, I., Reed, J., De La Rosa, R., Rose, L., Sugimoto, D., Araki, E., Chu, P. L., Wijayasinghe, N., & Norwood, P. (2018). Semaglutide added to basal insulin in type 2 diabetes (SUSTAIN 5): A randomized, controlled trial. *Journal of Clinical Endocrinology and Metabolism,**103*,. 10.1210/jc.2018-00070 10.1210/jc.2018-00070 PMC5991220 29688502

[98] Chudleigh, R. A., & Bain, S. C. (2020). Semaglutide injection for the treatment of adults with type 2 diabetes. *Expert Rev Clin Pharmacol*. 10.1080/17512433.2020.1776108 10.1080/17512433.2020.1776108 32476529

[99] Lee, S., & Lee, D. Y. (2017). Glucagon-like peptide-1 and glucagon-like peptide-1 receptor agonists in the treatment of type 2 diabetes. *Ann Pediatr Endocrinol Metab,**22*,. 10.6065/apem.2017.22.1.15 10.6065/apem.2017.22.1.15 PMC5401818 28443255

[100] Mahabaleshwarkar, R., & DeSantis, A. (2021). Metformin dosage patterns in type 2 diabetes patients in a real-world setting in the United States. *Diabetes Research And Clinical Practice,**172*,. 10.1016/j.diabres.2020.108531 10.1016/j.diabres.2020.108531 33157115

[101] Wilding, J. P. H., Batterham, R. L., Calanna, S., Davies, M., Van Gaal, L. F., Lingvay, I., McGowan, B. M., Rosenstock, J., Tran, M. T. D., Wadden, T. A., et al. (2021). Once-weekly semaglutide in adults with overweight or obesity. *New England Journal of Medicine,**384*,. 10.1056/nejmoa2032183 10.1056/NEJMoa2032183 33567185

[102] Rubino, D. M., Greenway, F. L., Khalid, U., O’Neil, P. M., Rosenstock, J., Sørrig, R., Wadden, T. A., Wizert, A., Garvey, W. T., Arauz-Pacheco, C., et al. (2022). Effect of weekly subcutaneous semaglutide vs daily liraglutide on body weight in adults with overweight or obesity without diabetes. *Journal of the American Medical Association,**327*, 138. 10.1001/jama.2021.23619 35015037 10.1001/jama.2021.23619 PMC8753508

[103] Røder, M. E. (2019). Clinical potential of treatment with semaglutide in type 2 diabetes patients. *Drugs Context,* 8. 10.7573/dic.212585 PMC6905643 31844422

[104] Davies, M., Færch, L., Jeppesen, O. K., Pakseresht, A., Pedersen, S. D., Perreault, L., Rosenstock, J., Shimomura, I., Viljoen, A., Wadden, T. A., et al. (2021). Semaglutide 2·4 Mg once a week in adults with overweight or obesity, and type 2 diabetes (STEP 2): A randomised, double-blind, double-dummy, placebo-controlled, phase 3 trial. *The Lancet,**397*,. 10.1016/S0140-6736(21)00213-0 10.1016/S0140-6736(21)00213-0 33667417

[105] Roglic, G., Unwin, N., Bennett, P. H., Mathers, C., Tuomilehto, J., Nag, S., Connolly, V., & King, H. (2005). The burden of mortality attributable to diabetes: Realistic estimates for the year 2000. *Diabetes Care,**28*, 2130–2135. 10.2337/DIACARE.28.9.2130 16123478 10.2337/diacare.28.9.2130

[106] Lin, Y., & Sun, Z. (2010). Current views on type 2 diabetes. *Journal of Endocrinology,* 204. 10.1677/JOE-09-0260 PMC2814170 19770178

[107] Colberg, S. R., Sigal, R. J., Fernhall, B., Regensteiner, J. G., Blissmer, B. J., Rubin, R. R., Chasan-Taber, L., Albright, A. L., & Braun, B. (2010). Exercise and type 2 diabetes: The American College of Sports Medicine and the American Diabetes Association: Joint position statement. Diabetes Care 33. 10.2337/dc10-9990 PMC2992225 21115758

[108] 5, & Targets, G. (2016). *Diabetes care,**39*,. 10.2337/dc16-S008

[109] Sorli, C., Harashima, S., Tsoukas, G. M., Unger, J., Karsbøl, J. D., Hansen, T., & Bain, S. C. (2017). Efficacy and safety of once-weekly semaglutide monotherapy versus placebo in patients with type 2 diabetes (SUSTAIN 1): A double-blind, randomised, placebo-controlled, parallel-group, multinational, multicentre phase 3a trial. *Lancet Diabetes Endocrinol,**5*,. 10.1016/S2213-8587(17)30013-X 10.1016/S2213-8587(17)30013-X 28110911

[110] Ahrén, B., Masmiquel, L., Kumar, H., Sargin, M., Karsbøl, J. D., Jacobsen, S. H., & Chow, F. (2017). Efficacy and safety of once-weekly semaglutide versus once-daily sitagliptin as an add-on to metformin, thiazolidinediones, or both, in patients with type 2 diabetes (SUSTAIN 2): A 56-week, double-blind, phase 3a, randomised trial. *Lancet Diabetes Endocrinol,**5*,. 10.1016/S2213-8587(17)30092-X 10.1016/S2213-8587(17)30092-X 28385659

[111] Ahmann, A. J., Capehorn, M., Charpentier, G., Dotta, F., Henkel, E., Lingvay, I., Holst, A. G., Annett, M. P., & Aroda, V. R. (2018). Efficacy and safety of once-weekly semaglutide versus exenatide ER in subjects with type 2 diabetes (SUSTAIN 3): A 56-week, open-label, randomized clinical trial. In Proceedings of the Diabetes Care; Vol. 41. 10.2337/dc17-0417 29246950

[112] Aroda, V. R., Bain, S. C., Cariou, B., Piletič, M., Rose, L., Axelsen, M., Rowe, E., & DeVries, J. H. (2017). Efficacy and safety of once-weekly semaglutide versus once-daily insulin glargine as add-on to metformin (with or without sulfonylureas) in insulin-naive patients with type 2 diabetes (SUSTAIN 4): A randomised, open-label, parallel-group, multicentre, multinational, phase 3a trial. *Lancet Diabetes Endocrinol,**5*,. 10.1016/S2213-8587(17)30085-2 10.1016/S2213-8587(17)30085-2 28344112

[113] Marso, S. P., Bain, S. C., Consoli, A., Eliaschewitz, F. G., Jódar, E., Leiter, L. A., Lingvay, I., Rosenstock, J., Seufert, J., Warren, M. L., et al. (2016). Semaglutide and cardiovascular outcomes in patients with type 2 diabetes. *New England Journal of Medicine,**375*,. 10.1056/nejmoa1607141 10.1056/NEJMoa1607141 27633186

[114] Pratley, R. E., Aroda, V. R., Lingvay, I., Lüdemann, J., Andreassen, C., Navarria, A., & Viljoen, A. (2018). Semaglutide versus dulaglutide once weekly in patients with type 2 diabetes (SUSTAIN 7): A randomised, open-label, phase 3b trial. *Lancet Diabetes Endocrinol,**6*,. 10.1016/S2213-8587(18)30024-X 10.1016/S2213-8587(18)30024-X 29397376

[115] Lingvay, I., Catarig, A. M., Frias, J. P., Kumar, H., Lausvig, N. L., le Roux, C. W., Thielke, D., Viljoen, A., & McCrimmon, R. J. (2019). Efficacy and safety of once-weekly semaglutide versus daily canagliflozin as add-on to metformin in patients with type 2 diabetes (SUSTAIN 8): A double-blind, phase 3b, randomised controlled trial. *Lancet Diabetes Endocrinol,**7*,. 10.1016/S2213-8587(19)30311-0 10.1016/S2213-8587(19)30311-0 31540867

[116] Zinman, B., Bhosekar, V., Busch, R., Holst, I., Ludvik, B., Thielke, D., Thrasher, J., Woo, V., & Philis-Tsimikas, A. (2019). Semaglutide once weekly as add-on to SGLT-2 inhibitor therapy in type 2 diabetes (SUSTAIN 9): A randomised, placebo-controlled trial. *Lancet Diabetes Endocrinol,**7*, 356–367. 10.1016/S2213-8587(19)30066-X 30833170 10.1016/S2213-8587(19)30066-X

[117] Capehorn, M. S., Catarig, A. M., Furberg, J. K., Janez, A., Price, H. C., Tadayon, S., Vergès, B., & Marre, M. (2020). Efficacy and safety of once-weekly semaglutide 1.0 Mg vs once-daily liraglutide 1.2 Mg as add-on to 1–3 oral antidiabetic drugs in subjects with type 2 diabetes (SUSTAIN 10). *Diabetes & Metabolism,**46*,. 10.1016/j.diabet.2019.101117 10.1016/j.diabet.2019.101117 31539622

[118] Kellerer, M., Kaltoft, M. S., Lawson, J., Nielsen, L. L., Strojek, K., Tabak, Ö., & Jacob, S. (2022). Effect of once-weekly semaglutide versus thrice-daily insulin aspart, both as add-on to metformin and optimized insulin glargine treatment in participants with type 2 diabetes (SUSTAIN 11): A randomized, open-label, multinational, phase 3b trial. *Diabetes, Obesity & Metabolism,**24*,. 10.1111/dom.14765 10.1111/dom.14765 PMC9545869 35546450

[119] Frías, J. P., Auerbach, P., Bajaj, H. S., Fukushima, Y., Lingvay, I., Macura, S., Søndergaard, A. L., Tankova, T. I., Tentolouris, N., & Buse, J. B. (2021). Efficacy and safety of once-weekly semaglutide 2·0 Mg versus 1·0 Mg in patients with type 2 diabetes (sustain forte): A double-blind, randomised, phase 3B trial. *Lancet Diabetes Endocrinol,**9*,. 10.1016/S2213-8587(21)00174-1 10.1016/S2213-8587(21)00174-1 34293304

[120] Seino, Y., Terauchi, Y., Osonoi, T., Yabe, D., Abe, N., Nishida, T., Zacho, J., & Kaneko, S. (2018). Safety and efficacy of semaglutide once weekly vs sitagliptin once daily, both as monotherapy in Japanese people with type 2 diabetes. *Diabetes, Obesity & Metabolism,**20*,. 10.1111/dom.13082 10.1111/dom.13082 PMC5813234 28786547

[121] Kaku, K., Yamada, Y., Watada, H., Abiko, A., Nishida, T., Zacho, J., & Kiyosue, A. (2018). Safety and efficacy of once-weekly semaglutide vs additional oral antidiabetic drugs in Japanese people with inadequately controlled type 2 diabetes: A randomized trial. *Diabetes, Obesity & Metabolism,**20*,. 10.1111/dom.13218 10.1111/dom.13218 PMC5969242 29322610

[122] Ji, L., Dong, X., Li, Y., Li, Y., Lim, S., Liu, M., Ning, Z., Rasmussen, S., Skjøth, T. V., Yuan, G., et al. (2021). Efficacy and safety of once-weekly semaglutide versus once-daily sitagliptin as add-on to metformin in patients with type 2 diabetes in sustain China: A 30-week, double-blind, phase 3a, randomized trial. *Diabetes, Obesity & Metabolism,**23*,. 10.1111/dom.14232 10.1111/dom.14232 PMC7839591 33074557

[123] Feng, Z., Tong, W. K., Zhang, X., & Tang, Z. (2023). Cost-effectiveness analysis of once-daily oral semaglutide versus placebo and subcutaneous glucagon-like peptide-1 receptor agonists added to insulin in patients with type 2 diabetes in China. *Frontiers In Pharmacology,**14*,. 10.3389/fphar.2023.1226778 10.3389/fphar.2023.1226778 PMC10445164 37621313

[124] Chen, J., Yin, D., & Dou, K. (2023). Intensified glycemic control by HbA1c for patients with coronary heart disease and type 2 diabetes: A review of findings and conclusions. *Cardiovasc Diabetol,* 22. 10.1186/s12933-023-01875-8 PMC10288803 37349787

[125] Hausner, H., Derving Karsbøl, J., Holst, A. G., Jacobsen, J. B., Wagner, F. D., Golor, G., & Anderson, T. W. (2017). Effect of semaglutide on the pharmacokinetics of metformin, warfarin, atorvastatin and digoxin in healthy subjects. *Clinical Pharmacokinetics,**56*,. 10.1007/s40262-017-0532-6 10.1007/s40262-017-0532-6 PMC5648738 28349387

[126] Bennett, W. L., Maruthur, N. M., Singh, S., Segal, J. B., Wilson, L. M., Chatterjee, R., Marinopoulos, S. S., Puhan, M. A., Ranasinghe, P., & Block, L. (2011). Comparative effectiveness and safety of medications for type 2 diabetes: An update including new drugs and 2-drug combinations. *Annals Of Internal Medicine,* 154. 10.7326/0003-4819-154-9-201105030-00336 PMC3733115 21403054

[127] Bethel, M. A., Patel, R. A., Merrill, P., Lokhnygina, Y., Buse, J. B., Mentz, R. J., Pagidipati, N. J., Chan, J. C., Gustavson, S. M., Iqbal, N., et al. (2018). Cardiovascular outcomes with glucagon-like peptide-1 receptor agonists in patients with type 2 diabetes: A meta-analysis. *Lancet Diabetes Endocrinol,**6*,. 10.1016/S2213-8587(17)30412-6 10.1016/S2213-8587(17)30412-6 29221659

[128] Smits, M. M., Van Raalte, D. H., & Corrigendum. (2021). Safety of semaglutide. *Front Endocrinol (Lausanne),**12*,. 10.3389/fendo.2021.786732 10.3389/fendo.2021.786732 PMC8631750 34858353

[129] Nagendra, L., BG, H., Sharma, M., & Dutta, D. (2023). Semaglutide and cancer: A systematic review and meta-analysis: Semaglutide and cancer. *Diabetes and Metabolic Syndrome: Clinical Research and Reviews,**17*,. 10.1016/j.dsx.2023.102834 10.1016/j.dsx.2023.102834 37531876

[130] Hansen, B. B., Nuhoho, S., Ali, S. N., Dang-Tan, T., Valentine, W. J., Malkin, S. J. P., & Hunt, B. (2020). Oral semaglutide versus injectable glucagon-like peptide-1 receptor agonists: A cost of control analysis. *Journal Of Medical Economics,**23*,. 10.1080/13696998.2020.1722678 10.1080/13696998.2020.1722678 31990244

