# Supplementary material for: The effect of GLP-1R agonists on the medical triad of obesity, diabetes, and cancer
Source: Cancer Metastasis Rev. 2024 May 27;43(4):1297–314. doi: 10.1007/s10555-024-10192-9 (PMC11554930; doi:10.1007/s10555-024-10192-9)
Supplement: Supplementary file 1 — Supplementary Material 1 [file 10555_2024_10192_MOESM1_ESM.docx]

**The Effect of GLP-1R agonists on The Medical Triad of Obesity, Diabetes, and Cancer**

**Shahad Sabaawi Ibrahim ^1,†^· Raghad Sabaawi Ibrahim ^1,†^· Batoul Arabi ^1^· Aranka Brockmueller ^2^· Mehdi Shakibaei ^2^· Dietrich Busselberg ^1*^**

^1^ Weill Cornell Medicine-Qatar, Education City, Qatar Foundation, Doha 24144, Qatar

^2^ Chair of Vegetative Anatomy, Institute of Anatomy, Faculty of Medicine, LMU Munich, Pettenkoferstr. 11, D-80336, Munich, Germany

^*^ Correspondence: dib2015@qatar-med.cornell.edu

^†^ These authors contributed equally to this work.

# Abstract

Glucagon-like peptide-1 receptor (GLP-1R) agonists have garnered significant attention for their therapeutic potential in addressing the interconnected health challenges of diabetes, obesity, and cancer. The role of GLP-1R in type 2 diabetes mellitus (T2DM) is highlighted, emphasizing its pivotal contribution to glucose homeostasis, promoting β-cell proliferation, and facilitating insulin release. GLP-1R agonists have effectively managed obesity by reducing hunger, moderating food intake, and regulating body weight. Beyond diabetes and obesity, GLP-1R agonists exhibit a multifaceted impact on cancer progression across various malignancies. The mechanisms underlying these effects involve the modulation of signaling pathways associated with cell growth, survival, and metabolism. However, the current literature reveals a lack of *in vivo* studies on specific GLP-1R agonists such as semaglutide, necessitating further research to elucidate its precise mechanisms and effects, particularly in cancer. While other GLP-1R agonists have shown promising outcomes in mitigating cancer progression, the association between some GLP-1R agonists and an increased risk of cancer remains a topic requiring more profound investigation. This calls for more extensive research to unravel the intricate relationships between the GLP-1R agonist and different cancers, providing valuable insights for clinicians and researchers alike.

**Keywords** Ozempic · Semaglutide · Cancer · Diabetes · Obesity · GLP1RA

# Introduction

Ozempic, a Glucagon-like peptide-1 receptor (GLP-1R) agonist containing the active ingredient semaglutide, is approved by the Food and Drug Administration for its potential therapeutic role in obesity and diabetes mellitus (DM) similar to other agonists within the GLP-1R family such as liraglutide . It not only successfully regulates blood sugar levels [1] but also reduces the appetite and, thereby, the weight of patients [2], especially in individuals with obesity and type 2 diabetes mellitus (T2DM). But why is there even a need for such a medication? And are potential long-term consequences, such as promoting the development or spread of cancer, receiving sufficient attention?

The number of overweight people has been increasing rapidly in recent years due to genetic, socio-economic, lifestyle, and cultural influences. Compared to the past, this not only affects adults but also numerous children and young people. Especially in American and European regions, at least 50% of residents now weigh more than the international standardized body mass index (BMI) recommends [3]. In this relation, a BMI of 30kg/m^2^ or higher is classified as obesity [4], whose causes lie in excessive calorie intake or reduced energy expenditure, and modern lifestyles promote both a lack of exercise and an unhealthy diet. Therefore, both these lifestyle factors and obesity itself represent a significant risk factor for metabolic disease T2DM [3,5].

DM is one of the most common metabolic diseases worldwide, with around 530 million people currently affected and an estimated 1.3 billion diabetes patients in 2050. The most common disease forms are the well-known types 1 and 2, severe diseases with chronic hyperglycemia [6]. While type 1 often manifests as an autoimmune insulin deficiency during childhood, T2DM predominantly develops throughout life. Epigenetic modifications trigger a genetic predisposition, which leads to insulin resistance [7]. Concretely, tobacco smoking, alcohol consumption, unbalanced diet, low fitness, increased BMI as well as unhealthy environmental influences are the key risk factors for the development of T2DM, and this form accounts for 96% of all DM cases [6]. Due to the disrupted metabolic processes, numerous signaling pathways become dysregulated, resulting in epigenetically induced inflammation. This triggers the development of malignant tumors in different tissues, for example, colorectal, liver, or breast cancer [8–10], creating a medical triad consisting of overweight, DM, and oncogenesis. Due to the close connection between this pathogenesis, it must be considered that drug manipulation of one of the diseases can also affect the other components of this interaction.

Therefore, this review presents the triangle relationship of obesity, DM as well as cancer development and elucidates the treatment of overweight-associated T2DM with semaglutide, focusing on the question of whether the development of these conditions can be combated or if any severe side effects should be considered of this therapy.

# The Medical Triad

Obesity, as well as DM, are conditions associated with a heightened risk of various cancers, including pancreatic, colorectal, breast, or liver cancer, and an increased mortality risk also accompanies them [8]. The link between these health issues and cancer risk is attributed to imbalances in the interaction of complex metabolic processes.

Obesity can be prevented by regulating body weight, dieting, and exercising [9] and although obesity is preventable, the increase in body fat allows the progression of metabolic diseases. Especially interesting, these metabolic diseases are associated with approximately 20% of cancer cases [10,11]. Obesity induces metabolic disturbances in adipose tissue, influencing the release of hormones, adipokines, inflammatory cytokines, growth factors, enzymes, and free fatty acids [12]. Notably, each 5% increase in BMI is estimated to correlate with a 10% rise in cancer-related deaths [13]. The altered physiology of adipose tissue in obesity releases metabolic substrates contributing to tumor cells’ proliferation, invasion, and metastasis. Two critical factors in this association are pro-inflammatory cytokines and adipokines. Pro-inflammatory cytokines produced by adipose tissue support tumor-promoting intercellular crosstalk in the tumor microenvironment [14], thus enhancing tumor cell progression, angiogenesis, and invasion as essential requirements for metastasis [15]. For example, breast and colorectal cancer progression was attributed to obesity [16], and in liver and gallbladder cancers, 51% of the cases are caused by this overweight disease[16]. However, calorie deficit, active lifestyle, behavior therapy, and drug therapy reduce inflammatory markers and regulate insulin levels commonly associated with cancer progression [17,18].

Adipokines such as adiponectin and leptin, derived from adipose tissue, play pivotal roles. The excessive expansion of adipose tissue in obesity disrupts adipokine secretion, fostering chronic low-grade inflammation and thereby contributing to the onset of metabolic disorders like obesity and T2DM. Adiponectin, inversely correlated with BMI, exhibits protective effects against carcinogenesis based on *in vitro* models. Leptin, implicated in inflammatory, mitogenic, and pro-angiogenic pathways, has been associated with breast cancer development, with studies indicating that inhibiting leptin signaling reduces the growth of breast cancer induced by carcinogens [12].

Moreover, high adiposity contributes to elevated serum estrogen levels, which, in excess, can promote tumor development by causing DNA damage, stimulating angiogenesis, and fostering cellular proliferation [19].

The presence of hyperglycemia and hyperinsulinemia in T2DM [20], leading to metabolic dysfunction, can also contribute to the proliferation and migration of cancer cells [21]. Cancer progression due to hyperglycemia was reported in multiple cancers, including breast, colorectal, brain, and pancreatic cancer [22–25]. Insulin is responsible for activating insulin receptors and insulin growth-like receptors. Moreover, elevated insulin levels resulting from hyperinsulinemia trigger insulin-like growth factor (IGF) signaling, activating key pathways such as phosphoinositide 3-kinase (PI3K)/protein kinase B (Akt)/mammalian target of rapamycin (mTOR) and mitogen-activated protein kinase (MAPK) [26]. These pathways, in turn, facilitate cancer cell growth, survival, motility, and resistance to drugs. Furthermore, it has long been theorized that cancer cells exhibit heightened glucose uptake and rely on glucose as a primary fuel for proliferation because it is a substrate cancer cells use as an energy source in aerobic glycolysis, resulting in tumor progression [27]. This phenomenon, known as the Warburg effect [28,29], is attributed to damaged mitochondria in cancer cells. Hence, anticancer therapy may include antidiabetic drugs targeting glucose metabolism and metabolic pathways that decrease the glucose uptake in cancer cells [30].

In summary, the intricate interplay of metabolic abnormalities, inflammatory responses, and hormonal influences in obesity and diabetes underscores their significant impact on cancer risk and mortality.

# The Treatment Complexities

The treatment of cancer in individuals who are both obese as well as diabetic poses significant challenges due to the intricate interplay between these conditions, and addressing these challenges involves navigating a complex landscape (Figure 1). One noteworthy obstacle is the potential need for higher chemotherapy doses in obese patients based on their body weight. However, this approach carries the inherent risk of heightened side effects and drug toxicity. In the case of obese individuals, an elevated BMI has been linked to increased interactional displacement, primarily stemming from the continuous movement of the skin and subcutaneous adiposity [31]. This displacement shift raises concerns about a potential reduction in the radiation dose reaching the target cells, leading to apprehensions about inadvertently overdosing patients with radiation and chemotherapy [31,32].

Moreover, managing diabetes during cancer treatment is a crucial aspect often overshadowed by the primary focus on cancer therapies. Chemotherapy, in particular, can influence blood sugar levels, causing fluctuations that need careful consideration. Additionally, the use of corticosteroids alongside chemotherapy to mitigate severe nausea and vomiting introduces another layer of complexity [33]. For diabetic patients, this poses a substantial threat, as corticosteroids are known to induce hyperglycemia [34]. The combination of decreased glucose uptake by the muscle and decreased glycogenesis further contributes to hyperglycemic conditions and complicates the already challenging task of treating cancer in individuals managing diabetes.

GLP-1R plays a significant role in the triad, making it an appealing target for treatment (Figure 1). Specifically, GLP-1R emerged as an important pharmacological target for addressing T2DM, as it actively contributes to maintaining glucose homeostasis while promoting both β cell proliferation and insulin release [35]. The impact of GLP-1R agonists such as semaglutide extends beyond diabetes control: they play a multifaceted role in regulating blood glucose levels by reducing hunger, moderating food intake, and managing body weight [36]. Notably, GLP-1R agonists inhibit cancer progression in some malignant tumors [37–40].


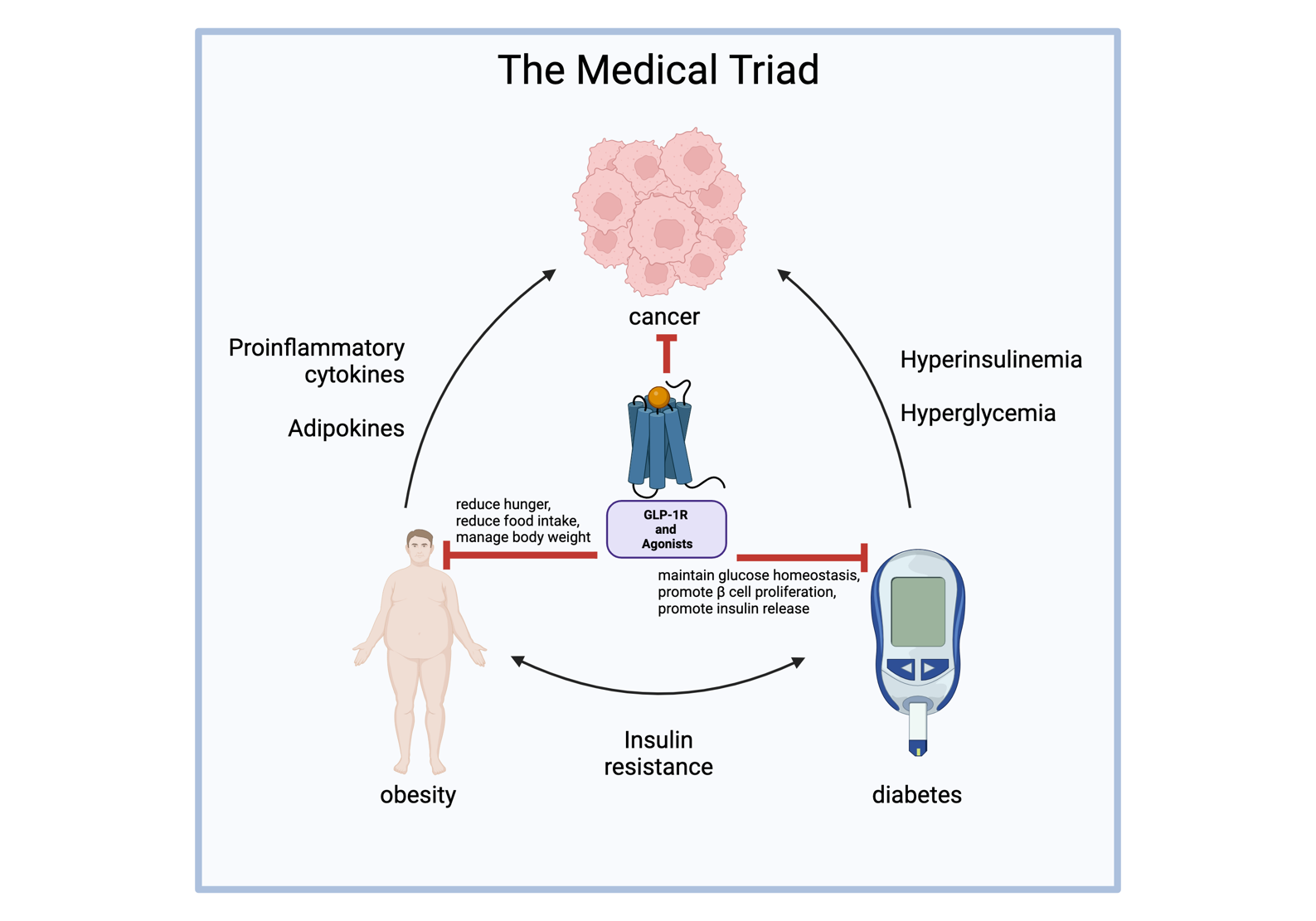


**Fig. 1:**The link between cancer, obesity, diabetes, and GLP-1R’s effect on them. Generated using BioRender.

# Glucagon-like peptide-1 receptor

The GLP-1R comprises seven hydrophobic transmembrane domains and a hydrophilic extracellular domain [41]. These receptors are expressed in multiple tissues, including the lung, stomach, intestine, liver, kidney, heart, pancreas, and regions in the central nervous system. Hence, it is a significant target of small-molecule drugs for signaling modulation (Figure 2) and treating various diseases [42]. The activation of GLP-1R is associated with glucose-induced insulin secretion and inhibition of α-cell glucagon release [43]. In addition, activation of GLP-1R results in a cascade that activates adenyl cyclase via Gα_s_, resulting in an increased secretion of cyclic adenosine monophosphate (cAMP) secretion and activating cAMP-dependent protein kinase (PKA). GLP-1R can also couple with adenyl cyclase using other Gα_s_ subtypes such as Gα_i_ and Gα_q_ [44,45]. An influx in calcium is reported upon activation of GLP-1R, and in combination with the activation of PKA, this results in insulin secretion [46].

GLP-1R reduces the inflammatory-induced response in the lungs, regulates oxidative stress and pulmonary function, and decreases excessive mucus production [47,48]. Furthermore, GLP-1R affects the mucosal membrane in the gastric tract by decreasing gastric secretions, which restrict gastric acid secretions and motility [49–51]. GLP-1R causes a reduction in hepatic steatosis and inflammation and increases fat metabolism mediated by increasing hepatic insulin sensitivity [52–54]. The GLP-1R directly influences renal functions by enhancing diuresis and natriuresis [55]. In diabetic kidney disease patients, a reduction in insulin levels, albuminuria, and the progression of renal failure was observed as a GLP-1R effect [56,57]. Moreover, GLP-1R is found in the heart and blood vessels and is beneficial in heart rate, vascular endothelium, atherosclerosis, and hypertension [58,59]. Since GLP-1R is a target in diabetes mellitus, it enhances the proliferation of β-cells and insulin secretion in the pancreas and reduces plasma glucose levels [60,61]. The significant role of GLP-1R in the brain is to regulate metabolic processes, energy expenditure, and neuronal excitability [62,63]. It also causes stress responses, satiety, and visceral illness’ in the central nervous system [63]. As an alarming finding, the activation of GLP-1R has been associated with developing thyroid cancer [64,65].

On the other hand, the activation of GLP-1R in the prostate attenuates cell proliferation and the progression of prostate cancer [66]. Although GLP-1R’s role is not fully understood or investigated in breast tissue when activated by different agonists, it may increase or decrease the progression of breast cancer [67,68]. The extensive role of GLP-1Rs and their agonists in other organs and tissues are shown in the overview in Figure 2.


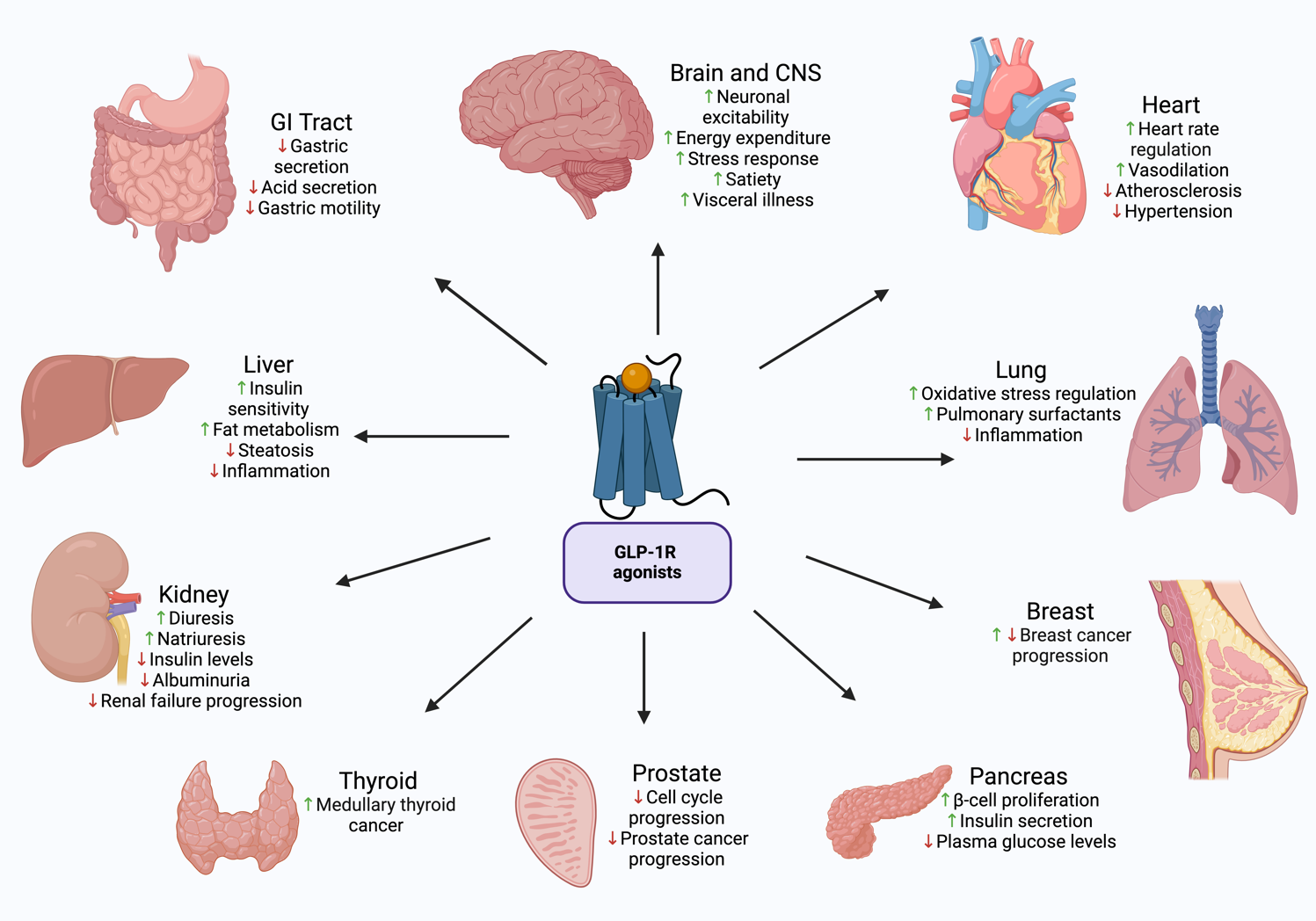


**Fig 2**: Effects of GLP-1RAs on organs. GLP-1R and agonists decrease hypertension, atherosclerosis, inflammation, plasma glucose levels, prostate cancer progression, insulin levels, albuminuria, renal failure progression, steatosis, gastric and acid secretion, and gastric motility levels amongst various organs in the body. GLP-1R and agonists increase heart rate regulation, vasodilation, oxidative stress regulation, pulmonary surfactants, β cell proliferation, insulin secretion and sensitivity, medullary thyroid cancer, diuresis, natriuresis, fat metabolism, neuronal excitability, energy expenditure, stress response, satiety, and visceral illness. For breasts, GLP-1R and agonists lead to an increase or decrease in breast cancer progression. Generated using BioRender.

# Glucagon-like peptide-1 receptor agonists

GLP-1R is targeted by GLP-1R agonists, which regulate diabetes and obesity. Although diabetic patients commonly use metformin, GLP-1R agonists serve as a beneficial therapeutic option for diabetic patients with metformin intolerance. GLP-1R agonists mimic hormones that activate biological responses in GLP-1R. GLP-1R agonists fall under two groups: human GLP-1 backbone agents and exendin-4 backbone agents. Dulaglutide, albiglutide, liraglutide, and semaglutide are human GLP-1 backbone agents. Exenatide and lixisenatide are classified as Exendin-4 backbone agents. In addition, semaglutide, liraglutide, and tirzepatide are GLP-1R agonists that are FDA approved [69]. Albiglutide was discontinued for low prescription rates rather than safety concerns [70,71]. All GLP-1R agonists have the same mode of function but differ in half-life duration. Exenatide has a half-life of 3.3 – 4 hours, and one dose is seen to be insufficient, so it is taken twice daily. Lixisenatide and liraglutide have half-lives of 2.6 hours and 12.6 – 14.3 hours, respectively, and are taken once daily. Dulaglutide, albiglutide, and semaglutide have half-lives that range from 4.7 – 5.5, 5.7 – 6.8, and 5.7 – 6.7 days, respectively [72].

GLP-1R agonists exhibit protective and regulatory effects on blood glucose levels but have been linked to the inhibition of tumor cell proliferation in most cancer cases, as seen *in vitro* and summarized in Table 1 [51,73]. The fact that GLP-1R agonists are sometimes introduced if the patient is intolerant to metformin or metformin is contraindicated, or when patients on metformin are not achieving their HbA1c goals [71] represents essential background information here.

**Table 1**: GLP-1R agonist’s effect on cancer in *in vitro* studies.

| Cancer type | Cell line | GLP-1R agonist | Concentration and duration | Serum concentration of drug | Results | References |
| --- | --- | --- | --- | --- | --- | --- |
| Colorectal cancer | LOVO | Liraglutide | 10^−5^ mol/L, 10^−8^ mol/L, 10^−11^ mol/L for 24, 48, or 72 hrs | N/A | ↓ PI3K  ↓ Akt  ↓ mTOR  ↓ Proliferation  ↓ Migration  ↓ Invasion  ↑ Apoptosis | [74] |
| Thyroid cancer | CA-77 | GLP-1 (7-37) agonists | 10^-8^ M from 3-48 hrs | N/A | ↑ cAMP  ↑ CGRP  ↑ CT | [75] |
| Breast cancer | MCF-7, MDA-MB-231, KPL-1 | Exendin-4 | 0.1-10 nM for 0-3 days | 0.44 ± 0.07 ng/mL | ↓ Proliferation  ↓ NF-κB activation  ↑ GLP-1R activation | [76] |
| Breast cancer | MCF-7, MDA-MB-231, KPL-1 | Exendin-4 combined with metformin | 10 nM for 0-3 days | N/A | ↓ Proliferation  ↑ GLP-1R activation | [77] |
| Breast cancer | MCF-7 | Exendin-4 | 0.25, 0.5, 1, 1.5, 2, 3, 5, 7.5, or 10 μM for 72 hrs | 5 μM | ↓ Migration  ↓ Invasion  ↓ Colony formation  ↓ Proliferation  ↑ Apoptosis | [78] |
| Breast cancer | MCF-7, MDA-MB-231, MDA-MB-468 | Exendin-4 | 1, 10, or 50 nM for 14 days | N/A | ↓ Colony formation  ↓ Cyclin D1  ↓ p-Akt  ↑ p53  ↑ p21  ↑ p38  ↑ cAMP | [79] |
| Breast cancer | 4T1, MCF-7, MDA-MB-231, MDA-MB-468, BT483, ZR751 | Liraglutide | 0, 10, 100, or 1000 nM for 24, 48, or 72 hrs | N/A | ↑ GLP-1R expression  ↑ Proliferation  ↑ Migration  ↑ ROS generation  ↑ NOX4 expression  ↑ VEGF | [67] |
| Prostate cancer | LNCap, PC3, ALVA-41, DU145 | Exendin-4 | 0.1-10 nM for 0, 24, 48, 72, or 96 hrs | N/A | ↓ ERK-MAPK pathway  ↓ Proliferation  ↑ cAMP | [80] |
| Prostate cancer | LNCap | Liraglutide combined with Docetaxel | 10, 20, 40, or 80 μM for 48 hrs | N/A | ↑ G2/M phase arrest  ↑ Apoptosis  ↓ p-ERK1/2  ↓ p-Akt | [81] |
| Prostate cancer | LNCap | Exenatide or liraglutide | 0, 1, 10, or 100 nM for 24 hrs | N/A | ↓ Proliferation  ↓ Cell viability  ↑ Apoptosis  ↑ Bax/Bcl-2 ratio  ↑ p38 MAPK activation | [82] |
| Pancreatic cancer | PANC-1, MiaPaCa-2, PANC | Liraglutide | 0, 10, 100, or 1000 nM for 48 hrs | N/A | ↑ GLP-1R expression  ↑ PKA expression  ↑ cAMP  ↑ Apoptosis  ↑ Bax  ↑ Caspase-3  ↓ Growth  ↓ Colony formation  ↓ NF-κB expression | [68] |
| Pancreatic cancer | MIA PaCa-2, PANC-1 | Liraglutide | 0, 10, 50, 100, 500 or 1000 nM for 72 hrs | N/A | ↓ Colony formation  ↓ Viability  ↓ Migration  ↓ Invasion  ↓ Proliferation  ↓ p-Akt | [83] |
| Colon cancer | CT26 | Exendin-4 | 5 or 50 nM | N/A | ↓ p-ERK1/2  ↓ GSK3  ↑ cAMP  ↓ Proliferation  ↑ Apoptosis  ↓ Colony formation  ↓ Viability | [84] |
| Ovarian cancer | SKOV3, OVCAR3, OVCAR4,  A2780, ES-2 | Exendin-4 | 0, 1, 10, or 100 nM for 96 hrs | N/A | ↓ Proliferation  ↓ Colony formation  ↓ Migration  ↓ Invasion  ↓ p-Akt  ↑ Apoptosis | [85] |

The effect of GLP-1R agonists liraglutide and exendin-4 was examined *in vitro* on LOVO, CA-77, MCF-7, MDA-MB-231, KPL-1, MB-468, 4TI, BT483, ZR751, LNCap, PC3, ALVA-41, DU145, LNCap, PANC-1, MiaPaCa-2, PANC, CT26, SKOV3, OVCAR3, OVCAR4, A2780, and ES-2 cell lines that are expressed in several cancers including colorectal, pancreatic, thyroid, breast, prostate, ovarian, and colon (Table 1).

Liraglutide concentrations of 10 – 1000 nM implemented for 24–72 hours showed an increase in apoptosis, G2/M phase arrest, Bax/Bcl-2 ratio, p38 MAPK activation, PKA expression, cAMP, caspase-3, GLP-1R expression, migration, ROS generation, NOX4 expression, VEGF, and proliferation of breast cancer in only one study. Nonetheless, a decrease in PI3K, Akt, mTOR, proliferation, migration, invasion, p-ERK1/2, growth, colony formation, inflammation represented by nuclear factor κB (NF-κB) expression, cell viability, and an overall decrease in proliferation was noted with applying liraglutide (Table 1).

In addition, exendin-4 at 0.1–100 nM concentrations for 24–96 hours increased GLP-1R activation, p53, p21, p38, cAMP, Bax/Bcl-2 ratio, and p38/MAPK activation. A reduction in proliferation, NF-κB activation, migration, invasion, migration, colony formation, Cyclin D1, p-Akt, ERK-MAPK pathway, cAMP, and GSK3 accompanied this increase (Table 1).

Figure 3 shows an overview of the mentioned effects of the two GLP-1R agonists and their influence on numerous signaling pathways.


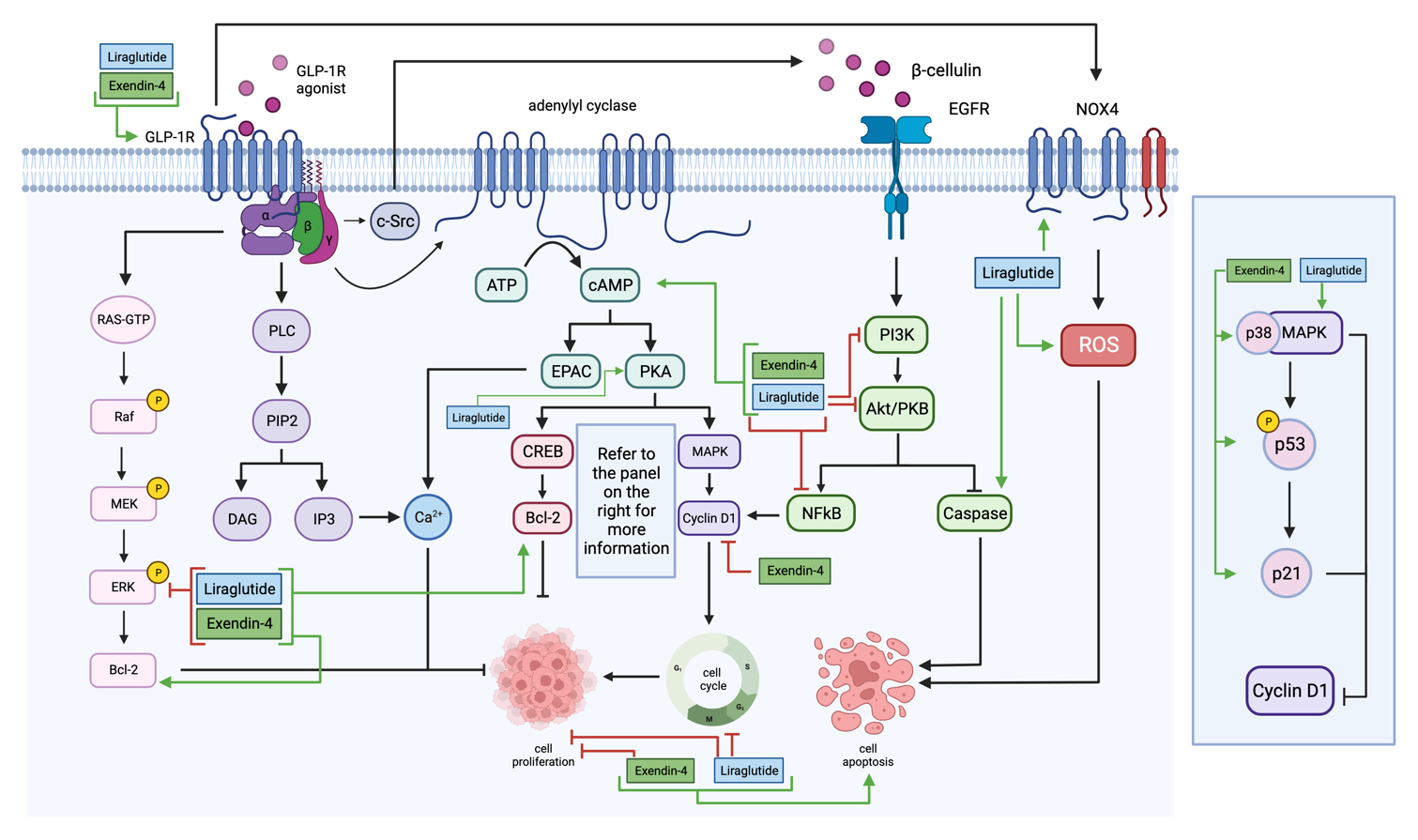


**Fig. 3:** Liraglutide and Exendin-4 decrease NF-κB, cell proliferation, and phosphorylated ERK in cancer. Liraglutide decreases Pl3K, Akt/PKB, and cell division while increasing GLP-1Rs, NOX4, ROS, caspase, MAPK, cAMP, Bcl-2, PKA, and cell apoptosis levels. Exendin-4 decreases Cylin D1 while increasing GLP-1Rs, MAPK, p21, p53, cAMP, Bcl-2, and cell apoptosis. Generated using BioRender.

Some of the significant GLP-1R agonists studied other than semaglutide are liraglutide and exendin-4. Both agonists affect cancer *in vitro* by decreasing the proliferation and metabolic pathways at varying concentrations. Liraglutide and exendin-4 are initially antidiabetic drugs but can affect tumorigenesis, suggesting GLP-1R agonists as a potential treatment for different cancers as found *in vivo* (Table 2). Notably, an increase in calcitonin (Figure 4), particularly observed in thyroid cancer, indicates cancer development [75], given its role as a tumor marker in medullary thyroid neoplasia [88]. More research is needed to understand the effect of other agonists, as cancer research is lacking. GLP-1R agonists are typically combined with metformin or other antidiabetic drugs. The combination of GLP-1R agonists with each other has not been studied previously, suggesting that due to their similar mode of action, there would not be an enhancement in therapy from this combination.


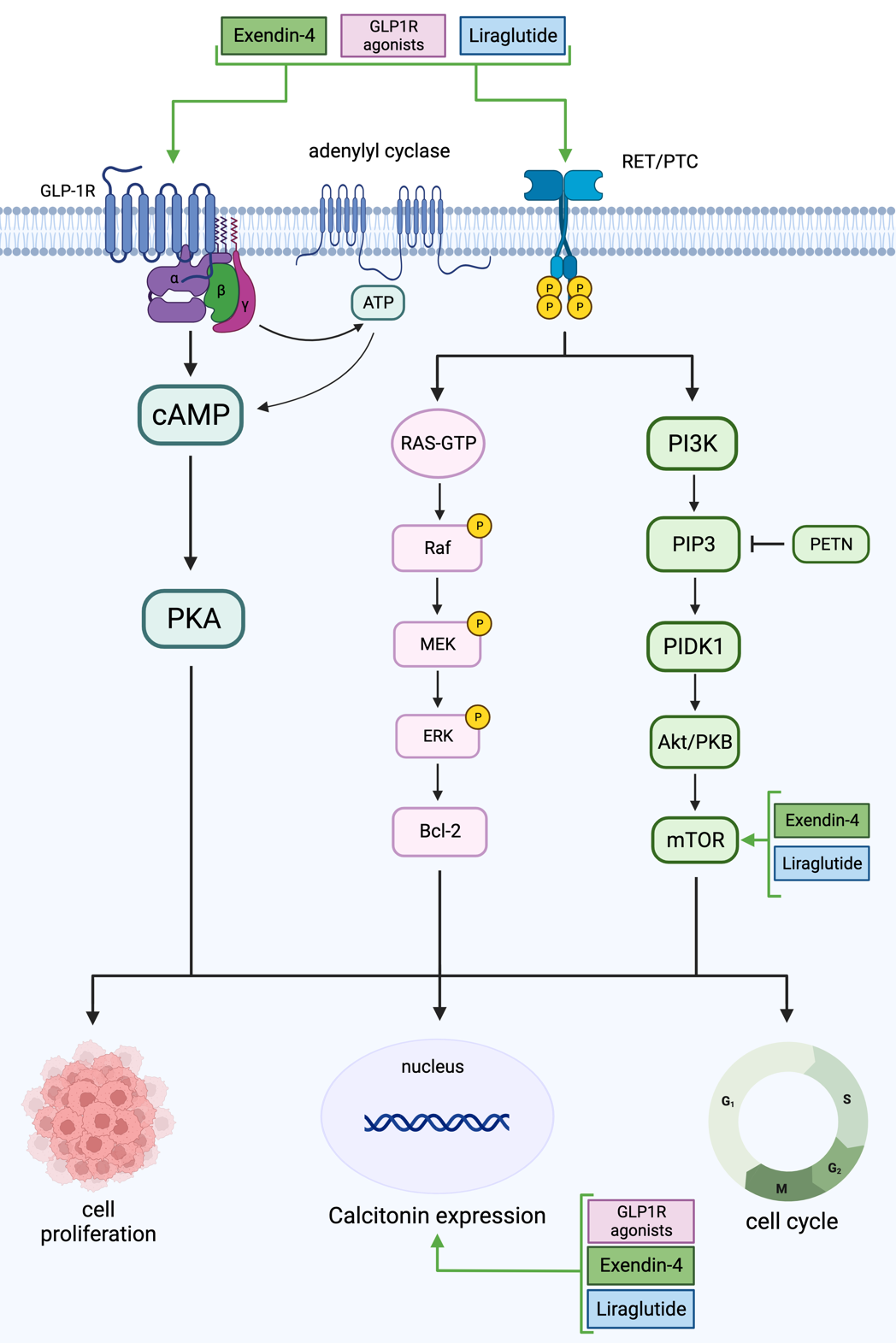


**Fig. 4:** GLP-1R agonists were studied in thyroid cancers. Both Liraglutide and Exendin-4 increase the levels of GLP-1R, RET/PTC, mTOR and calcitonin expression amongst patients with thyroid cancer. Generated using BioRender.

**Table 2**: GLP-1R agonist’s effect on cancer in *in vivo* studies

| Cancer type | Cell line | GLP-1R agonist | Concentration and duration | Results | References |
| --- | --- | --- | --- | --- | --- |
| Thyroid cancer | CD-1 wild-type mice | Liraglutide and exenatide | 0.03, 0.3, or 3.0 mg/kg for 13 weeks | ↑ Calcitonin  ↑ C-cell hyperplasia  ↑ GLP-1R  ↑ mTOR activation  ↑ pS6 | [89] |
| Breast cancer | MCF-7 cells into athymic nude mice | Exendin-4 and exandin | 300 pmol/kg body weight/day Ex-4 or 3 nmol/kg body weight/day exendin for 6-9 weeks | ↓ Tumor size  ↓ Proliferation  ↓ Ki67  ↓ NF-κB activation  ↑ Serum insulin | [76] |
| Breast cancer | MCF-7 cells into athymic mice | Exendin-4 combined with metformin | 300 pmol/kg body weight/day for 8 weeks | ↓ Tumor volume  ↓ Tumor weight  ↓ Ki67  ↑ GLP-1R | [77] |
| Breast cancer | MDA-MB-468 and MDA-MB-231 cells into athymic nude mice | Exendin-4 | 500 ng or 2 μg per day for 6 weeks | ↓ Tumor weight  ↓ Tumor size | [79] |
| Breast cancer | 4T1 cells in BALB/cfC3H mice | Liraglutide | 400 μg/kg for 2 weeks | ↑ Tumor volume  ↑ Metastasis | [67] |
| Prostate cancer | LNCap cells into athymic mice | Exendin-4 | 24 nmol/kg body weight/day or 300 pmol/kg body weight/day | ↓ Tumor size  ↓ P504S, prostate cancer marker  ↓ Ki67  ↓ Proliferation  ↓ p-ERK-MAPK | [80] |
| Prostate cancer | LNCap cells into athymic mice | Exendin-4 combined with metformin | 300 pmol/kg body weight/day for 6 weeks | ↓ Tumor volume  ↓ Tumor weight  ↓ Ki67  ↓ P504S  ↓ Proliferation | [90] |
| Pancreatic cancer | PANC-1 cells into nude mice | Liraglutide | 0.2 mg/kg twice daily for 4 weeks | ↑ Chemosensitivity  ↑ Bax  ↓ Tumor volume  ↓ Tumor weight  ↓ Ki67 | [68] |
| Pancreatic cancer | MIA PaCa-2 in male athymic nude mice | Liraglutide | 0.2 mg/kg for 4 weeks | ↓ Tumor growth | [83] |
| Colon cancer | CT26 cells into BALB/c mice | Exendin-4 | 10 nmol/kg for 2 weeks | ↑ Apoptosis  ↓ Proliferation | [84] |
| Ovarian cancer | SKOV-3 cells into female nude mice | Exendin-4 | 500 ng/day or 2 μg/day for 28 days | ↓ Tumor size  ↓ Tumor weight  ↓ Tumor volume | [85,91] |
| Intestinal cancer | Apc(Min/+) mice | Exendin-4 | For 1 month | ↑ Small bowel weight  ↑ Small bowel length  ↑ Large bowel weight | [91] |
| Liver cancer | N/A | Liraglutide | N/A | ↓ Body weight  ↓ Fasting blood glucose  ↓ Tumor lesions  ↓ Fat deposition  ↑ Insulin-positive β-cells | [92] |

The effect of GLP-1R agonists liraglutide and exendin-4 was examined *in vivo* on CD-1, MCF-1, MDA-MB-468, MDA-MB-231, 4T1, LNCap, PANC-1, MIA PaCa-2, CT26, SKOV-3, and Apc(Min/+) cell lines expressed in cancers such as thyroid, breast, prostate, pancreatic, colon, ovarian, intestinal, and liver (Table 2).

Both liraglutide and exendin-4 decrease the size, weight, and proliferation of pancreatic, breast, prostate, ovarian, intestinal, liver, and colon cancer. Liraglutide slows down and sometimes even inhibits tumor growth of cancers in mice in *in vivo* studies. Moreover, it down-regulates the protein levels of cell proliferation marker PCNA, decreases cell viability and number, and up-regulates the protein levels of pro-apoptotic markers [93]. In addition, this GLP-1R agonist activates AMPK, inhibiting the proliferation of various cancerous cells, making it a promising cancer treatment [94].

Exendin-4 significantly inhibits different cell lines and induces apoptosis through the mechanism modification of apoptosis-related genes, which plays a role in extrinsic pathways and cell survival genes [78]. Specifically in colon cancer and prostate cancer, exendin-4 increased intracellular cAMP levels while inhibiting glycogen synthase kinase 3 and ERK-MAPK activation, leading to an increase in apoptosis [95]. Moreover, exendin-4 inhibits migration, cell invasion, and colony formation of many cancers, making it a possible treatment for malignant cells, specifically in prostate cancer. Exendin-4 suppresses cell proliferation through the inhibition of ERK-MAPK [80]. Lastly, a combination of exendin-4 and metformin has been shown to attenuate different forms of cancer at a more noticeable rate [96,97].

# The popular GLP-1R agonist semaglutide

Semaglutide is a long-acting GLP-1R agonist structurally similar to GLP-1 but resistant to proteolytic cleavage [98] and is given as a subcutaneous injection to patients with T2D. This is because, in comparison to GLP-1, semaglutide has two amino acid substitutions, which makes it less vulnerable to degradation by the proteolytic enzyme dipeptidyl peptidase-4 (DPP-4) [99] and gives it the distinct advantage of increased albumin affinity [98]. Moreover, due to these substitutions, semaglutide has a prolonged half-life of approximately 168 hours, which is impressive given the knowledge that native GLP-1 has a half-life of 2 minutes, in comparison [100,101]. This progress in scientific development enables a one-week administration of the drug, making them an efficient alternative to other antidiabetic drugs, such as metformin, which requires two daily doses, and the updated metformin, which requires daily doses [102,103]. Furthermore, semaglutide is superior to other GLP-1R agonists, such as liraglutide, which uses similar pharmacological mechanisms but only has a half-life of 13 hours and therefore has to be injected subcutaneously daily. This is underscored by the results of a clinical trial comparing the development of obese adults who received either semaglutide or liraglutide in addition to nutritional advice and physical activity, and significantly greater weight loss was achieved in the semaglutide group [104].

Semaglutide, as a GLP-1R agonist, works very similarly to GLP-1 by potentiating glucose-stimulated insulin secretion from the pancreatic β-cells while suppressing glucagon secretion by pancreatic α-cells [105]. Therefore, antidiabetic medication decreases blood sugar levels, reduces body weight through a reduction in appetite [96], and lowers glycated hemoglobin (HbA1c), all while having a low risk of causing hypoglycemia [106]. Although GLP-1R agonists exhibit protective and regulatory effects on blood glucose levels, they have been positively correlated with tumor progression in patients with diabetes [51,73].

# Semaglutide’s role in the medical triad of diabetes, obesity, and cancer

The prevalence of T2DM has increased significantly in the past decades. It is likely to be the fifth most common cause of death, following an 8% attribution to the mortality rate in the USA, Canada, and the Middle East [107]. Patients with T2DM tend to secrete less insulin following a glucose-heavy meal possibly due to decreased levels of glucagon-like peptide-1 (GLP-1) [98]. Characterized by insulin resistance, gradual progressive loss of insulin secretion by β-cells, and being heavily driven by being overweight or obese [100], T2DM leads to hyperglycemia, excessive urine production, increased risk of cardiovascular disease, and changes in energy metabolism [108]. To lessen the effects of these symptoms, patients with T2DM are encouraged to improve their eating lifestyles and increase their physical activity [109].

Nevertheless, despite the positive outcomes of exercise and diet, recommended glycemic levels (e.g., HbA1c <7.0%, 53.0 mmol/mol for nonpregnant adults) [110] may sometimes be challenging to achieve. Therefore, the addition of glucose-lowering agents is recommended by the American and European Diabetes Associations to control and/or minimize the risk of cardiovascular disease and microvascular complications [105]. In this regard, semaglutide is becoming increasingly important, as demonstrated in Table 3, which summarizes the previous results of phase 3 clinical trials.

**Table 3**: Semaglutide’s effect on type 2 diabetes and obesity in phase 3 trials.

| Sustain Number | Number of participants | Concentration and treatment duration | Monotherapy vs. Combined therapy | Results | Reference |
| --- | --- | --- | --- | --- | --- |
| 1 | 388 | 0.5, or 1.0 mg for 30 weeks | Monotherapy | ↓ HbA1c  ↓ Body weight  ↑ Nausea  ↑ Diarrhea | [111] |
| 2 | 1231 | 0.5, or 1.0 mg for 56 weeks | Both, combined with 100mg sitagliptin | Monotherapy:  ↓ HbA1c  ↓ Body weight  Both:  ↑ Hypoglycemia  ↑ Nausea  ↑ Diarrhea | [112] |
| 3 | 813 | 1.0 mg for 56 weeks | Monotherapy | ↓ HbA1c  ↓Fasting insulin  ↓Insulin resistance  ↓Plasma glucagon  ↓ Body weight  ↑ Gastrointestinal adverse events  ↑ Neoplasms | [113] |
| 4 | 1089 | 0.5, or 1.0 mg for 30 weeks | Combined with metformin alone or with sulfonylurea | ↓ HbA1c  ↓ Body weight | [114] |
| 5* | 397 | 0.5, 1.0 mg for 30 weeks | Combined with basal insulin | ↓ HbA1c  ↓ Body weight  ↑ Neoplasms  ↑ Pancreatic cancer | [99] |
| 6 | 3297 | 0.5, 1.0 mg for 104 weeks | Monotherapy | ↓ HbA1c  ↓ Body weight  ↓Cardiovascular death  ↓ Nonfatal stroke and myocardial infarction  ↓ Mean systolic blood pressure  ↑ Diabetic retinopathy complications | [115] |
| 7 | 1201 | 0.5, 1.0 mg for 24 weeks | Monotherapy | ↓ HbA1c  ↓ Body weight | [116] |
| 8 | 788 | 1.0 mg for 52 weeks | Combined with metformin | ↓ HbA1c  ↓ Body weight  ↑ Gastrointestinal adverse events | [117] |
| 9 | 302 | 1.0 mg for 37 weeks | Combined with sodium-glucose cotransporter-2 (SGLT-2) inhibitors and metformin or sulfonylurea | ↓ HbA1c  ↓ Body weight  ↑ Gastrointestinal adverse events | [118] |
| 10 | 577 | 1.0 mg for 30 weeks | Combined with oral antidiabetic drugs | ↓ HbA1c  ↓ Body weight  ↓ FPG  ↓ SMBG  ↑ Gastrointestinal adverse events  ↑ Improvement in total cholesterol and triglycerides | [119] |
| 11 | 1748 | 1.0 mg for 52 weeks | Combined with metformin and insulin glargine | ↓ HbA1c  ↓ Body weight  ↓ Systolic blood pressure  ↑ Gastrointestinal adverse events | [120] |
| FORTE | 961 | 1.0 or 2.0 for 40 weeks | Combined with metformin and with or without sulfonylurea | ↓ HbA1c  ↓ Body weight  ↓ Blood pressure  ↑ Gastrointestinal adverse events | [121] |
| Japan | 308 | 0.5mg, or 1.0 mg for 30 weeks | Monotherapy | ↓ HbA1c  ↓ Body weight  ↓ Blood pressure  ↓ VLDL cholesterol and triglycerides  ↑ Treatment of emergent adverse events | [122] |
| Japan | 601 | 0.5, or 1.0 mg for 63 weeks | Both monotherapy and combined with an oral antidiabetic drug | ↓ HbA1c  ↓ Body weight  ↓ Insulin ratios  ↓ Blood pressure  ↓ All lipids (except free fatty acids and HDL cholesterol)  ↑ Treatment of emergent adverse events  ↑ Pancreatic enzymes | [123] |
| China | 868 | 0.5, or 1.0 mg for 30 weeks | Combined with metformin | ↓ HbA1c  ↓ Body weight  ↓ Systolic blood pressure  ↓ Total cholesterol  ↑ Gastrointestinal adverse events  ↑ Amylase and lipase | [124] |

* Represents a single case

Semaglutide was tested in clinical trials on individuals with obesity and diabetes. Phase three of the clinical trials was conducted using semaglutide concentrations ranging from 0.5–2.0 mg for 30–104 weeks. A decrease in body weight, blood pressure, HbA1c, fasting insulin_,_ insulin resistance_,_ plasma glucagon, total cholesterol, lipids, FPG, SMBG, cardiovascular death, nonfatal stroke, and myocardial infarction (Table 3). However, an increase in gastrointestinal adverse events, diarrhea, hypoglycemia, nausea, neoplasm, treatment-emergent adverse events, pancreatic enzymes, diabetic retinopathy complications, and pancreatic cancer was observed to accompany the treatment using semaglutide. Several clinical trials implemented a combination of semaglutide treatments with sulfonylurea, sitagliptin, basal insulin, metformin, or other antidiabetic drugs (Table 3).

Altogether, clinical trials in phase 3 that applied semaglutide in monotherapy and combined with other antidiabetic drugs yielded similar results. However, suppose semaglutide is reduced to $1,711.03 per year. In that case it will be considered cost-effective, and the preferable treatment compared to other GLP-1R agonists and antidiabetic drugs [125]. The decrease in HbA1c caused by semaglutide is accompanied by a reduction in body weight, which is unique to semaglutide as an antidiabetic medication. Therefore, injections of semaglutide can assist individuals in maintaining a healthier lifestyle with a decrease in the rate of potential cardiovascular diseases [126]. The effect of semaglutide alone is sufficient to result in positive outcomes. Still, these positive results can be enhanced when in combination with other drugs, such as metformin, which allows for an effective treatment plan with no increase in adverse symptoms usually seen with the treatment of semaglutide alone [123,124,127,128]. Furthermore, the possible promotion of cancer cell growth is increasingly being discussed with contradictory results (Table 4) that require clarification.

**Table 4**: Semaglutide’s involvement with cancer as an adverse effect.

| Cancer type | Concentration and duration | Number of participants | Number of events in placebo | Number of events caused by semaglutide | Reference |
| --- | --- | --- | --- | --- | --- |
| Neoplasms | 0.5 mg, 1.0mg for 56 weeks | n=818 | n=0 | n=14 | [112] |
| EAC-confirmed Neoplasms | 1.0 mg for 56 weeks | n=813 | N/A | n=15 | [113] |
| EAC-confirmed Neoplasms | 0.5, or 1.0 mg for 30 weeks | n=397 | n=1 | n=5 | [99] |
| Neoplasms | 0.5, or 1.0 mg for 104 weeks | n=136 | n=70 | n=66 | [115] |
| Neoplasms | 0.5, or 1.0 mg for 24 weeks | n=601 | N/A | n=6 | [116] |
| Neoplasms | 1.0 mg for 52 weeks | n=367 | N/A | n=3 | [117] |
| Neoplasms | 1.0 mg for 37 weeks | n=302 | n=5 | n=4 | [118] |
| Neoplasms | 1.0 mg for 30 weeks | n=577 | N/A | n=9 | [119] |
| Neoplasms | 1.0 mg for 52 weeks | n=874 | N/A | n=11 | [120] |
| Neoplasms | 0.5, or 1.0 mg for 63 weeks | n=595 | N/A | n=44 | [123] |
| Neoplasms | 0.5, or 1.0 mg for 30 weeks | n=578 | N/A | n=23 | [124] |
| Thyroid | 1.0 mg for 104 weeks | n=1648 | n=4 | n=1 | [130] |
| Thyroid | 1.0 mg for 56 weeks | n=818 | n=0 | n=1 | [112] |
| Bladder | 1.0 mg for 30 weeks | n=205 | N/A | n=1 | [122] |
| Bladder | 1.0 mg for 56 weeks | n=818 | n=0 | n=1 | [112] |
| Pancreatic | 0.5 mg for 30 weeks | n=722 | N/A | n=1 | [114] |
| Pancreatic | 1.0 mg for 30 weeks | n=397 | N/A | n=1 | [99] |
| Colorectal | 0.5 mg for 30 weeks | n=397 | n=1 | n=1 | [99] |
| Colorectal | 0.5, or 1.0 mg for 63 weeks | n=595 | N/A | n=18 | [123] |

Semaglutide is associated with increased neoplasm and tumorigenesis, specifically in the thyroid, bladder, colorectal, and pancreas. Doses of 0.5–1.0 mg yielded 1–155 cases of cancer development in the treatment period of 30–104 weeks. Lower cases were reported in the thyroid, bladder, colorectal, and pancreas compared to a treatment period of 104 weeks with 0.5 mg of semaglutide, which yielded 155 cases of neoplasm. On the contrary, for the same treatment period but at a drug concentration of 1.0 mg, there was only 1 case of thyroid cancer (Table 4).

Semaglutide, among other antidiabetic drugs, has shown an association with cancer as it alters the rates of tumorigenesis and proliferation. This GLP-R1 agonist seems to increase oncogenesis in multiple tissues, including the thyroid, bladder, pancreatic, and colorectal [99,112,123].

Pharmaceutical companies have issued a warning about the use of formulations of semaglutide with those who have thyroid cancer or are at risk of developing it [131]. Despite some studies showing cases of cancer development, the numbers reported are as minimal as one case. There is no conclusive evidence that semaglutide induced cancer development in tissue, which may imply that the development of cancer may pertain to other causes rather than semaglutide application [132]. On the contrary, several authors report mitigation of cancer proliferation using the same dose of 0.5–1.0 mg semaglutide ingested by diabetic patients and for similar periods. The increase in semaglutide is gradual and can be altered to scale up every 30 days when ingested orally. This accumulation may aid semaglutide’s action against cancer cells [133]. There is a pool of research on the effect of liraglutide and exendin on cancer. However, there is a lack of research data on the impact of semaglutide *in vitro* and *in vivo*, which limits its efficiency in tumor therapy. Further research must be conducted to understand the effects of semaglutide on cancer as it belongs to the GLP-1R family along with liraglutide and exendin and may provide similar results.

# Conclusions

Semaglutide, like many other GLP-1R agonists, is used in diabetes and obesity to decrease glucose levels and manage body weight, which plays a role in tumorigenesis. The insufficient *in vivo* studies on semaglutide and limited *in vitro* research raises concerns about the imperative for more comprehensive investigations into its effects. Specifically, there is a need to elucidate a descriptive mechanism through which semaglutide reduces diabetes and obesity and potentially influences cancer. Although there is a lack of direct studies on semaglutide, the observed actions align with those of other GLP-1R agonists, indicating a potential impact on cancer. There is a notable increase in thyroid cancer with the use of GLP-1R agonists, including semaglutide. The use of GLP-1R agonists increases calcitonin gene-related peptide (CGRP) in thyroid cancer. Overexpression of pS6, mTOR activation, calcitonin, and C-cell hyperplasia reported in *in vivo* suggest the increased proliferation and tumorigenesis [75,89]. Despite the cancer cases recorded with the use of semaglutide, other factors may have contributed to cancer development with no association with semaglutide [132]. However, whether Semaglutide’s effect is mitigating or exacerbating remains unclear, emphasizing the necessity for further research on the outcome of GLP-1R agonists on cancer, specifically thyroid cancer.

**Abbreviations**

Akt – protein kinase B; BMI – body mass index; cAMP - cyclic adenosine monophosphate; CGRP - calcitonin gene-related peptide; DM - diabetes mellitus; DPP-4 - dipeptidyl peptidase-4; GLP-1 – glucagon-like peptide-1; GLP-1R - glucagon-like peptide-1 receptor; HbA1c - glycated hemoglobin; IGF - insulin-like growth factor; MAPK - mitogen-activated protein kinase; mTOR - mammalian target of rapamycin; NF-κB - nuclear factor κB; PI3K - phosphoinositide 3-kinase; PKA - cAMP-dependent protein kinase; SGLT-2 - sodium-glucosecotransporter-2; T2DM - type 2 diabetes mellitus

**Acknowledgments**

**Author contributions**

Conceptualization, S.S.I., and R.S.I.; resources, D.B.; writing—original draft preparation, S.S.I., R.S.I., and B.A.; writing—review and editing, M.S., A.B. D.B., B.A., S.S.I. and R.S.I.; visualization, S.S.I and. R.S.I.; supervision, D.B. All authors have read and agreed to the published version of the manuscript.

**Funding**

Open Access funding provided by the Qatar National Library. This work was supported by a National Priorities Research Program grant (NPRP 14S-0311–210033; awarded to Professor Dr. Dietrich Büsselberg, January 2023-Current) from the Qatar National Research Fund (QNRF, a member of Qatar Foundation). The statements made herein are solely the responsibility of the authors.

**Declarations**

**Competing interests** The authors declare no competing interest.

**Data availability** N/A

**Conflict of interest** The authors declare no conflict of interest.

**Ethical approval** N/A

**Informed consent** N/A

# References

1. Iijima, T.; Shibuya, M.; Ito, Y.; Terauchi, Y. Effects of Switching from Liraglutide to Semaglutide or Dulaglutide in Patients with Type 2 Diabetes: A Randomized Controlled Trial. *J Diabetes Investig* **2023**, *14*, doi:10.1111/jdi.14000.

2. Kadowaki, T.; Isendahl, J.; Khalid, U.; Lee, S.Y.; Nishida, T.; Ogawa, W.; Tobe, K.; Yamauchi, T.; Lim, S. Semaglutide Once a Week in Adults with Overweight or Obesity, with or without Type 2 Diabetes in an East Asian Population (STEP 6): A Randomised, Double-Blind, Double-Dummy, Placebo-Controlled, Phase 3a Trial. *Lancet Diabetes Endocrinol* **2022**, *10*, 193–206, doi:10.1016/S2213-8587(22)00008-0.

3. Pérez Rodrigo, C. Current Mapping of Obesity. *Nutr Hosp* **2013**, *28 Suppl 5*, 21–31, doi:10.3305/NH.2013.28.SUP5.6915.

4. Chooi, Y.C.; Ding, C.; Magkos, F. The Epidemiology of Obesity. *Metabolism* **2019**, *92*, doi:10.1016/j.metabol.2018.09.005.

5. Männistö, S.; Kontto, J.; Kataja-Tuomola, M.; Albanes, D.; Virtamo, J. High Processed Meat Consumption Is a Risk Factor of Type 2 Diabetes in the Alpha-Tocopherol, Beta-Carotene Cancer Prevention Study. *British Journal of Nutrition* **2010**, *103*, doi:10.1017/S0007114510000073.

6. Ong, K.L.; Stafford, L.K.; McLaughlin, S.A.; Boyko, E.J.; Vollset, S.E.; Smith, A.E.; Dalton, B.E.; Duprey, J.; Cruz, J.A.; Hagins, H.; et al. Global, Regional, and National Burden of Diabetes from 1990 to 2021, with Projections of Prevalence to 2050: A Systematic Analysis for the Global Burden of Disease Study 2021. *The Lancet* **2023**, *402*, doi:10.1016/S0140-6736(23)01301-6.

7. Buzzetti, R.; Maddaloni, E.; Gaglia, J.; Leslie, R.D.; Wong, F.S.; Boehm, B.O. Adult-Onset Autoimmune Diabetes. *Nat Rev Dis Primers* **2022**, *8*, doi:10.1038/s41572-022-00390-6.

8. Scully, T.; Ettela, A.; LeRoith, D.; Gallagher, E.J. Obesity, Type 2 Diabetes, and Cancer Risk. *Front Oncol* 2021, *10*.

9. Wirth, A.; Wabitsch, M.; Hauner, H. The Prevention and Treatment of Obesity. *Dtsch Arztebl Int* **2014**, *111*, doi:10.3238/arztebl.2014.0705.

10. Avgerinos, K.I.; Spyrou, N.; Mantzoros, C.S.; Dalamaga, M. Obesity and Cancer Risk: Emerging Biological Mechanisms and Perspectives. *Metabolism* **2019**, *92*, 121–135, doi:10.1016/J.METABOL.2018.11.001.

11. Preuss, H.G.; Bagchi, M.; Bagchi, D.; Kaats, G.R. Obesity and Cancer. *Oncologist* **2010**, *15*, 197–204, doi:10.1634/THEONCOLOGIST.2009-0285.

12. Kim, D.S.; Scherer, P.E. Obesity, Diabetes, and Increased Cancer Progression. *Diabetes Metab J* 2021, *45*.

13. López-Suárez, A. Burden of Cancer Attributable to Obesity, Type 2 Diabetes and Associated Risk Factors. *Metabolism* 2019, *92*.

14. Buhrmann, C.; Shayan, P.; Brockmueller, A.; Shakibaei, M. Resveratrol Suppresses Cross-Talk between Colorectal Cancer Cells and Stromal Cells in Multicellular Tumor Microenvironment: A Bridge between in Vitro and in Vivo Tumor Microenvironment Study. *Molecules* **2020**, *25*, doi:10.3390/molecules25184292.

15. Coussens, L.M.; Werb, Z. Inflammation and Cancer. *Nature* **2002**, *420*, 860–867, doi:10.1038/NATURE01322.

16. Islami, F.; Goding Sauer, A.; Gapstur, S.M.; Jemal, A. Proportion of Cancer Cases Attributable to Excess Body Weight by US State, 2011-2015. *JAMA Oncol* **2019**, *5*, doi:10.1001/jamaoncol.2018.5639.

17. Pati, S.; Irfan, W.; Jameel, A.; Ahmed, S.; Shahid, R.K. Obesity and Cancer: A Current Overview of Epidemiology, Pathogenesis, Outcomes, and Management. *Cancers (Basel)* 2023, *15*.

18. De Pergola, G.; Silvestris, F. Obesity as a Major Risk Factor for Cancer. *J Obes* 2013, *2013*.

19. Bhardwaj, P.; Iyengar, N.M.; Zahid, H.; Carter, K.M.; Byun, D.J.; Choi, M.H.; Sun, Q.; Savenkov, O.; Louka, C.; Liu, C.; et al. Obesity Promotes Breast Epithelium DNA Damage in Women Carrying a Germline Mutation in BRCA1 or BRCA2. *Sci Transl Med* **2023**, *15*, doi:10.1126/scitranslmed.ade1857.

20. Harreiter, J.; Roden, M. Diabetes Mellitus—Definition, Classification, Diagnosis, Screening and Prevention (Update 2019). *Wien Klin Wochenschr* **2019**, *131*, doi:10.1007/s00508-019-1450-4.

21. Nolen, L. The Effect of Glucose on Rapid Cancer Cell Proliferation & Waste. *Oncology Times* **2022**, *44*, doi:10.1097/01.cot.0000892616.48647.47.

22. Khajah, M.A.; Khushaish, S.; Luqmani, Y.A. Glucose Deprivation Reduces Proliferation and Motility, and Enhances the Anti-Proliferative Effects of Paclitaxel and Doxorubicin in Breast Cell Lines in Vitro. *PLoS One* **2022**, *17*, doi:10.1371/journal.pone.0272449.

23. Lin, C.Y.; Lee, C.H.; Huang, C.C.; Lee, S.T.; Guo, H.R.; Su, S. Bin Impact of High Glucose on Metastasis of Colon Cancer Cells. *World J Gastroenterol* **2015**, *21*, doi:10.3748/wjg.v21.i7.2047.

24. Derr, R.L.; Ye, X.; Islas, M.U.; Desideri, S.; Saudek, C.D.; Grossman, S.A. Association between Hyperglycemia and Survival in Patients with Newly Diagnosed Glioblastoma. *Journal of Clinical Oncology* **2009**, *27*, doi:10.1200/JCO.2008.19.1098.

25. Rahn, S.; Zimmermann, V.; Viol, F.; Knaack, H.; Stemmer, K.; Peters, L.; Lenk, L.; Ungefroren, H.; Saur, D.; Schäfer, H.; et al. Diabetes as Risk Factor for Pancreatic Cancer: Hyperglycemia Promotes Epithelial-Mesenchymal-Transition and Stem Cell Properties in Pancreatic Ductal Epithelial Cells. *Cancer Lett* **2018**, *415*, doi:10.1016/j.canlet.2017.12.004.

26. Qiang, J.K.; Lipscombe, L.L.; Lega, I.C. Association between Diabetes, Obesity, Aging, and Cancer: Review of Recent Literature. *Transl Cancer Res* 2020, *9*.

27. Granja, S.; Pinheiro, C.; Reis, R.; Martinho, O.; Baltazar, F. Glucose Addiction in Cancer Therapy: Advances and Drawbacks. *Curr Drug Metab* **2015**, *16*, doi:10.2174/1389200216666150602145145.

28. Brockmueller, A.; Sameri, S.; Liskova, A.; Zhai, K.; Varghese, E.; Samuel, S.M.; Büsselberg, D.; Kubatka, P.; Shakibaei, M. Resveratrol’s Anti-Cancer Effects through the Modulation of Tumor Glucose Metabolism. *Cancers (Basel)* **2021**, *13*, 1–35, doi:10.3390/CANCERS13020188.

29. Samec, M.; Liskova, A.; Koklesova, L.; Samuel, S.M.; Zhai, K.; Buhrmann, C.; Varghese, E.; Abotaleb, M.; Qaradakhi, T.; Zulli, A.; et al. Flavonoids against the Warburg Phenotype—Concepts of Predictive, Preventive and Personalised Medicine to Cut the Gordian Knot of Cancer Cell Metabolism. *EPMA Journal* 2020, *11*.

30. Klil-Drori, A.J.; Azoulay, L.; Pollak, M.N. Cancer, Obesity, Diabetes, and Antidiabetic Drugs: Is the Fog Clearing? *Nat Rev Clin Oncol* **2017**, *14*, 85–99, doi:10.1038/NRCLINONC.2016.120.

31. Slawinski, C.G.V.; Barriuso, J.; Guo, H.; Renehan, A.G. Obesity and Cancer Treatment Outcomes: Interpreting the Complex Evidence. *Clin Oncol (R Coll Radiol)* **2020**, *32*, 591–608, doi:10.1016/J.CLON.2020.05.004.

32. Vucenik, I.; Stains, J.P. Obesity and Cancer Risk: Evidence, Mechanisms, and Recommendations. *Ann N Y Acad Sci* **2012**, *1271*, 37–43, doi:10.1111/J.1749-6632.2012.06750.X.

33. Rowbottom, L.; Stinson, J.; McDonald, R.; Emmenegger, U.; Cheng, S.; Lowe, J.; Giotis, A.; Cheon, P.; Chow, R.; Pasetka, M.; et al. Retrospective Review of the Incidence of Monitoring Blood Glucose Levels in Patients Receiving Corticosteroids with Systemic Anticancer Therapy. *Ann Palliat Med* **2015**, *4*, 70–77, doi:10.3978/J.ISSN.2224-5820.2015.04.07.

34. Shahid, R.K.; Ahmed, S.; Le, D.; Yadav, S. Diabetes and Cancer: Risk, Challenges, Management and Outcomes. *Cancers (Basel)* 2021, *13*.

35. Zhu, L.; Zhou, J.; Pan, Y.; Lv, J.; Liu, Y.; Yu, S.; Zhang, Y. Glucagon-like Peptide-1 Receptor Expression and Its Functions Are Regulated by Androgen. *Biomedicine and Pharmacotherapy* **2019**, *120*, doi:10.1016/j.biopha.2019.109555.

36. Drucker, D.J. GLP-1 Physiology Informs the Pharmacotherapy of Obesity. *Mol Metab* 2022, *57*.

37. Nomiyama, T.; Yanase, T. GLP-1 Receptor Agonist as Treatment for Cancer as Well as Diabetes: Beyond Blood Glucose Control. *Expert Rev Endocrinol Metab* **2016**, 1–8, doi:10.1080/17446651.2016.1191349.

38. Koehler, J.A.; Kain, T.; Drucker, D.J. Glucagon-Like Peptide-1 Receptor Activation Inhibits Growth and Augments Apoptosis in Murine CT26 Colon Cancer Cells. *Endocrinology* **2011**, *152*, 3362–3372, doi:10.1210/en.2011-1201.

39. Wang, L.; Wang, W.; Kaelber, D.C.; Xu, R.; Berger, N.A. GLP-1 Receptor Agonists and Colorectal Cancer Risk in Drug-Naive Patients With Type 2 Diabetes, With and Without Overweight/Obesity. *JAMA Oncol* **2024**, *10*, 256, doi:10.1001/jamaoncol.2023.5573.

40. Skriver, C.; Friis, S.; Knudsen, L.B.; Catarig, A.-M.; Clark, A.J.; Dehlendorff, C.; Mørch, L.S. Potential Preventive Properties of GLP-1 Receptor Agonists against Prostate Cancer: A Nationwide Cohort Study. *Diabetologia* **2023**, *66*, 2007–2016, doi:10.1007/s00125-023-05972-x.

41. *Handbook of Hormones*; 2016;

42. Johnson, L.R.; Barret, K.E.; Gishan, F.K.; Merchant, J.L.; Said, H.M.; Wood, J.D. *Physiology of the Gastrointestinal Tract*; 2006; Vol. 1–2;.

43. Rowlands, J.; Heng, J.; Newsholme, P.; Carlessi, R. Pleiotropic Effects of GLP-1 and Analogs on Cell Signaling, Metabolism, and Function. *Front Endocrinol (Lausanne)* 2018, *9*.

44. Carlessi, R.; Chen, Y.; Rowlands, J.; Cruzat, V.F.; Keane, K.N.; Egan, L.; Mamotte, C.; Stokes, R.; Gunton, J.E.; Bittencourt, P.I.H. De; et al. GLP-1 Receptor Signalling Promotes β-Cell Glucose Metabolism via MTOR-Dependent HIF-1α Activation. *Sci Rep* **2017**, *7*, doi:10.1038/S41598-017-02838-2.

45. Detka, J.; Głombik, K. Insights into a Possible Role of Glucagon-like Peptide-1 Receptor Agonists in the Treatment of Depression. *Pharmacological Reports* **2021**, *73*, 1020, doi:10.1007/S43440-021-00274-8.

46. Koole, C.; Savage, E.E.; Christopoulos, A.; Miller, L.J.; Sexton, P.M.; Wootten, D. Minireview: Signal Bias, Allosterism, and Polymorphic Variation at the GLP-1R: Implications for Drug Discovery. *Mol Endocrinol* **2013**, *27*, 1234–1244, doi:10.1210/ME.2013-1116.

47. Sato, T.; Shimizu, T.; Fujita, H.; Imai, Y.; Drucker, D.J.; Seino, Y.; Yamada, Y. GLP-1 Receptor Signaling Differentially Modifies the Outcomes of Sterile vs Viral Pulmonary Inflammation in Male Mice. *Endocrinology* **2020**, *161*, doi:10.1210/ENDOCR/BQAA201.

48. Wang, W.; Mei, A.; Qian, H.; Li, D.; Xu, H.; Chen, J.; Yang, H.; Min, X.; Li, C.; Cheng, L.; et al. The Role of Glucagon-Like Peptide-1 Receptor Agonists in Chronic Obstructive Pulmonary Disease. *Int J Chron Obstruct Pulmon Dis* **2023**, *18*, 129, doi:10.2147/COPD.S393323.

49. Broide, E.; Bloch, O.; Ben-Yehudah, G.; Cantrell, D.; Shirin, H.; Rapoport, M.J. GLP-1 Receptor Is Expressed in Human Stomach Mucosa: Analysis of Its Cellular Association and Distribution within Gastric Glands. *J Histochem Cytochem* **2013**, *61*, 649–658, doi:10.1369/0022155413497586.

50. Holst, J.J.; Andersen, D.B.; Grunddal, K.V. Actions of Glucagon-like Peptide-1 Receptor Ligands in the Gut. *Br J Pharmacol* **2022**, *179*, 727–742, doi:10.1111/BPH.15611.

51. Zhao, X.; Wang, M.; Wen, Z.; Lu, Z.; Cui, L.; Fu, C.; Xue, H.; Liu, Y.; Zhang, Y. GLP-1 Receptor Agonists: Beyond Their Pancreatic Effects. *Front Endocrinol (Lausanne)* 2021, *12*.

52. Wang, X.C.; Gusdon, A.M.; Liu, H.; Qu, S. Effects of Glucagon-like Peptide-1 Receptor Agonists on Non-Alcoholic Fatty Liver Disease and Inflammation. *World J Gastroenterol* 2014, *20*.

53. Yabut, J.M.; Drucker, D.J. Glucagon-like Peptide-1 Receptor-Based Therapeutics for Metabolic Liver Disease. *Endocr Rev* **2023**, *44*, 14–32, doi:10.1210/ENDREV/BNAC018.

54. Wong, C.; Lee, M.H.; Yaow, C.Y.L.; Chin, Y.H.; Goh, X.L.; Ng, C.H.; Lim, A.Y.L.; Muthiah, M.D.; Khoo, C.M. Glucagon-Like Peptide-1 Receptor Agonists for Non-Alcoholic Fatty Liver Disease in Type 2 Diabetes: A Meta-Analysis. *Front Endocrinol (Lausanne)* **2021**, *12*, doi:10.3389/FENDO.2021.609110.

55. Liu, X.; Patel, K.P.; Zheng, H. Role of Renal Sympathetic Nerves in GLP-1 (Glucagon-Like Peptide-1) Receptor Agonist Exendin-4-Mediated Diuresis and Natriuresis in Diet-Induced Obese Rats. *J Am Heart Assoc* **2021**, *10*, doi:10.1161/JAHA.121.022542.

56. Górriz, J.L.; Soler, M.J.; Navarro-González, J.F.; García-Carro, C.; Puchades, M.J.; D’marco, L.; Castelao, A.M.; Fernández-Fernández, B.; Ortiz, A.; Górriz-Zambrano, C.; et al. GLP-1 Receptor Agonists and Diabetic Kidney Disease: A Call of Attention to Nephrologists. *J Clin Med* 2020, *9*.

57. Granata, A.; Maccarrone, R.; Anzaldi, M.; Leonardi, G.; Pesce, F.; Amico, F.; Gesualdo, L.; Corrao, S. GLP-1 Receptor Agonists and Renal Outcomes in Patients with Diabetes Mellitus Type 2 and Diabetic Kidney Disease: State of the Art. *Clin Kidney J* 2022, *15*.

58. Baggio, L.L.; Yusta, B.; Mulvihill, E.E.; Cao, X.; Streutker, C.J.; Butany, J.; Cappola, T.P.; Margulies, K.B.; Drucker, D.J. GLP-1 Receptor Expression Within the Human Heart. *Endocrinology* **2018**, *159*, doi:10.1210/en.2018-00004.

59. Del Olmo-Garcia, M.I.; Merino-Torres, J.F. GLP-1 Receptor Agonists and Cardiovascular Disease in Patients with Type 2 Diabetes. *J Diabetes Res* **2018**, *2018*, doi:10.1155/2018/4020492.

60. Hou, Y.; Ernst, S.A.; Heidenreich, K.; Williams, J.A. Glucagon-like Peptide-1 Receptor Is Present in Pancreatic Acinar Cells and Regulates Amylase Secretion through CAMP. *Am J Physiol Gastrointest Liver Physiol* **2016**, *310*, doi:10.1152/ajpgi.00293.2015.

61. Doyle, M.E.; Egan, J.M. Mechanisms of Action of Glucagon-like Peptide 1 in the Pancreas. *Pharmacol Ther* 2007, *113*.

62. Liu, J.; Pang, Z.P. Glucagon-like Peptide-1 Drives Energy Metabolism on the Synaptic Highway. *FEBS J* **2016**, *283*, 4413–4423, doi:10.1111/FEBS.13785.

63. Jessen, L.; Smith, E.P.; Ulrich-Lai, Y.; Herman, J.P.; Seeley, R.J.; Sandoval, D.; D’Alessio, D. Central Nervous System GLP-1 Receptors Regulate Islet Hormone Secretion and Glucose Homeostasis in Male Rats. *Endocrinology* **2017**, *158*, doi:10.1210/en.2016-1826.

64. Gier, B.; Butler, P.C.; Lai, C.K.; Kirakossian, D.; DeNicola, M.M.; Yeh, M.W. Glucagon like Peptide-1 Receptor Expression in the Human Thyroid Gland. *Journal of Clinical Endocrinology and Metabolism* **2012**, *97*, doi:10.1210/jc.2011-2407.

65. Bezin, J.; Gouverneur, A.; Penichon, M.; Mathieu, C.; Garrel, R.; Hillaire-Buys, D.; Pariente, A.; Faillie, J.L. GLP-1 Receptor Agonists and the Risk of Thyroid Cancer. *Diabetes Care* **2023**, *46*, 384–390, doi:10.2337/DC22-1148.

66. Shigeoka, T.; Nomiyama, T.; Kawanami, T.; Hamaguchi, Y.; Horikawa, T.; Tanaka, T.; Irie, S.; Motonaga, R.; Hamanoue, N.; Tanabe, M.; et al. Activation of Overexpressed Glucagon-like Peptide-1 Receptor Attenuates Prostate Cancer Growth by Inhibiting Cell Cycle Progression. *J Diabetes Investig* **2020**, *11*, doi:10.1111/jdi.13247.

67. Liu, Z. zhao; Duan, X. xian; Yuan, M. ci; Yu, J.; Hu, X.; Han, X.; Lan, L.; Liu, B. wei; Wang, Y.; Qin, J. fang Glucagon-like Peptide-1 Receptor Activation by Liraglutide Promotes Breast Cancer through NOX4/ROS/VEGF Pathway. *Life Sci* **2022**, *294*, doi:10.1016/j.lfs.2022.120370.

68. Zhao, H.J.; Jiang, X.; Hu, L.J.; Yang, L.; Deng, L.D.; Wang, Y.P.; Ren, Z.P. Activation of GLP-1 Receptor Enhances the Chemosensitivity of Pancreatic Cancer Cells. *J Mol Endocrinol* **2020**, *64*, doi:10.1530/JME-19-0186.

69. Collins, L.; Costello, R.A. Glucagon-Like Peptide-1 Receptor Agonists. *StatPearls* **2023**.

70. Rosenstock, J.; Nino, A.; Soffer, J.; Erskine, L.; Acusta, A.; Dole, J.; Carr, M.C.; Mallory, J.; Home, P. Impact of a Weekly Glucagon-like Peptide 1 Receptor Agonist, Albiglutide, on Glycemic Control and on Reducing Prandial Insulin Use in Type 2 Diabetes Inadequately Controlled on Multiple Insulin Therapy: A Randomized Trial. *Diabetes Care* **2020**, *43*, doi:10.2337/dc19-2316.

71. Latif, W.; Lambrinos, K.J.; Rodriguez, R. Compare and Contrast the Glucagon-Like Peptide-1 Receptor Agonists (GLP1RAs). *StatPearls* **2023**.

72. Nauck, M.A.; Quast, D.R.; Wefers, J.; Meier, J.J. GLP-1 Receptor Agonists in the Treatment of Type 2 Diabetes – State-of-the-Art. *Mol Metab* **2021**, *46*, 101102, doi:10.1016/j.molmet.2020.101102.

73. Meier, J.J. GLP-1 Receptor Agonists for Individualized Treatment of Type 2 Diabetes Mellitus. *Nat Rev Endocrinol* 2012, *8*.

74. Tong, G.; Peng, T.; Chen, Y.; Sha, L.; Dai, H.; Xiang, Y.; Zou, Z.; He, H.; Wang, S. Effects of GLP-1 Receptor Agonists on Biological Behavior of Colorectal Cancer Cells by Regulating PI3K/AKT/MTOR Signaling Pathway. *Front Pharmacol* **2022**, *13*, doi:10.3389/fphar.2022.901559.

75. Lamari, Y.; Boissard, C.; Moukhtar, M.S.; Jullienne, A.; Rosselin, G.; Garel, J.M. Expression of Glucagon-like Peptide 1 Receptor in a Murine C Cell Line: Regulation of Calcitonin Gene by Glucagon-like Peptide 1. *FEBS Lett* **1996**, *393*, doi:10.1016/0014-5793(96)00895-2.

76. Iwaya, C.; Nomiyama, T.; Komatsu, S.; Kawanami, T.; Tsutsumi, Y.; Hamaguchi, Y.; Horikawa, T.; Yoshinaga, Y.; Yamashita, S.; Tanaka, T.; et al. Exendin-4, a Glucagonlike Peptide-1 Receptor Agonist, Attenuates Breast Cancer Growth by Inhibiting NF-KB Activation. *Endocrinology* **2017**, *158*, doi:10.1210/en.2017-00461.

77. Tanaka, Y.; Iwaya, C.; Kawanami, T.; Hamaguchi, Y.; Horikawa, T.; Shigeoka, T.; Yanase, T.; Kawanami, D.; Nomiyama, T. Combined Treatment with Glucagon-like Peptide-1 Receptor Agonist Exendin-4 and Metformin Attenuates Breast Cancer Growth. *Diabetol Int* **2022**, *13*, doi:10.1007/s13340-021-00560-z.

78. Fidan-Yaylalı, G.; Dodurga, Y.; Seçme, M.; Elmas, L. Antidiabetic Exendin-4 Activates Apoptotic Pathway and Inhibits Growth of Breast Cancer Cells. *Tumor Biology* **2016**, *37*, doi:10.1007/s13277-015-4104-9.

79. Ligumsky, H.; Wolf, I.; Israeli, S.; Haimsohn, M.; Ferber, S.; Karasik, A.; Kaufman, B.; Rubinek, T. The Peptide-Hormone Glucagon-like Peptide-1 Activates CAMP and Inhibits Growth of Breast Cancer Cells. *Breast Cancer Res Treat* **2012**, *132*, doi:10.1007/s10549-011-1585-0.

80. Nomiyama, T.; Kawanami, T.; Irie, S.; Hamaguchi, Y.; Terawaki, Y.; Murase, K.; Tsutsumi, Y.; Nagaishi, R.; Tanabe, M.; Morinaga, H.; et al. Exendin-4, a GLP-1 Receptor Agonist, Attenuates Prostate Cancer Growth. *Diabetes* **2014**, *63*, doi:10.2337/db13-1169.

81. Eftekhari, S.; Montazeri, H.; Tarighi, P. Synergistic Anti-Tumor Effects of Liraglutide, a Glucagon-like Peptide-1 Receptor Agonist, along with Docetaxel on LNCaP Prostate Cancer Cell Line. *Eur J Pharmacol* **2020**, *878*, doi:10.1016/j.ejphar.2020.173102.

82. Li, X.N.; Bu, H.M.; Ma, X.H.; Lu, S.; Zhao, S.; Cui, Y.L.; Sun, J. Glucagon-like Peptide-1 Analogues Inhibit Proliferation and Increase Apoptosis of Human Prostate Cancer Cells in Vitro. *Experimental and Clinical Endocrinology and Diabetes* **2017**, *125*, doi:10.1055/s-0042-112368.

83. Zhao, H.; Wang, L.; Wei, R.; Xiu, D.; Tao, M.; Ke, J.; Liu, Y.; Yang, J.; Hong, T. Activation of Glucagon-like Peptide-1 Receptor Inhibits Tumourigenicity and Metastasis of Human Pancreatic Cancer Cells via PI3K/Akt Pathway. *Diabetes Obes Metab* **2014**, *16*, doi:10.1111/dom.12291.

84. Koehler, J.A.; Kain, T.; Drucker, D.J. Glucagon-like Peptide-1 Receptor Activation Inhibits Growth and Augments Apoptosis in Murine CT26 Colon Cancer Cells. *Endocrinology* **2011**, *152*, doi:10.1210/en.2011-1201.

85. He, W.; Yu, S.; Wang, L.; He, M.; Cao, X.; Li, Y.; Xiao, H. Exendin-4 Inhibits Growth and Augments Apoptosis of Ovarian Cancer Cells. *Mol Cell Endocrinol* **2016**, *436*, doi:10.1016/j.mce.2016.07.032.

86. Hinnen, D. Glucagon-Like Peptide 1 Receptor Agonists for Type 2 Diabetes. *Diabetes Spectr* **2017**, *30*, 202–210, doi:10.2337/DS16-0026.

87. Mahapatra, M.K.; Karuppasamy, M.; Sahoo, B.M. Semaglutide, a Glucagon like Peptide-1 Receptor Agonist with Cardiovascular Benefits for Management of Type 2 Diabetes. *Rev Endocr Metab Disord* 2022, *23*.

88. Verbeek, H.H.G.; de Groot, J.W.B.; Sluiter, W.J.; Muller Kobold, A.C.; van den Heuvel, E.R.; Plukker, J.T.M.; Links, T.P. Calcitonin Testing for Detection of Medullary Thyroid Cancer in People with Thyroid Nodules. *Cochrane Database of Systematic Reviews* 2020, *2020*.

89. Madsen, L.W.; Knauf, J.A.; Gotfredsen, C.; Pilling, A.; Sjögren, I.; Andersen, S.; Andersen, L.; De Boer, A.S.; Manova, K.; Barlas, A.; et al. GLP-1 Receptor Agonists and the Thyroid: C-Cell Effects in Mice Are Mediated via the GLP-1 Receptor and Not Associated with RET Activation. *Endocrinology* **2012**, *153*, doi:10.1210/en.2011-1864.

90. Tsutsumi, Y.; Nomiyama, T.; Kawanami, T.; Hamaguchi, Y.; Terawaki, Y.; Tanaka, T.; Murase, K.; Motonaga, R.; Tanabe, M.; Yanase, T.; et al. Combined Treatment with Exendin-4 and Metformin Attenuates Prostate Cancer Growth. *PLoS One* **2015**, *10*, doi:10.1371/journal.pone.0139709.

91. Koehler, J.A.; Baggio, L.L.; Yusta, B.; Longuet, C.; Rowland, K.J.; Cao, X.; Holland, D.; Brubaker, P.L.; Drucker, D.J. GLP-1R Agonists Promote Normal and Neoplastic Intestinal Growth through Mechanisms Requiring Fgf7. *Cell Metab* **2015**, *21*, doi:10.1016/j.cmet.2015.02.005.

92. Kojima, M.; Takahashi, H.; Kuwashiro, T.; Tanaka, K.; Mori, H.; Ozaki, I.; Kitajima, Y.; Matsuda, Y.; Ashida, K.; Eguchi, Y.; et al. Glucagon-like Peptide-1 Receptor Agonist Prevented the Progression of Hepatocellular Carcinoma in a Mouse Model of Nonalcoholic Steatohepatitis. *Int J Mol Sci* **2020**, *21*, doi:10.3390/ijms21165722.

93. Lu, R.; Yang, J.; Wei, R.; Ke, J.; Tian, Q.; Yu, F.; Liu, J.; Zhang, J.; Hong, T. Synergistic Anti-Tumor Effects of Liraglutide with Metformin on Pancreatic Cancer Cells. *PLoS One* **2018**, *13*, doi:10.1371/journal.pone.0198938.

94. Zhao, W.; Zhang, X.; Zhou, Z.; Sun, B.; Gu, W.; Liu, J.; Zhang, H. Liraglutide Inhibits the Proliferation and Promotes the Apoptosis of MCF-7 Human Breast Cancer Cells through Downregulation of MicroRNA-27a Expression. *Mol Med Rep* **2018**, *17*, doi:10.3892/mmr.2018.8475.

95. Iwaya, C.; Nomiyama, T.; Komatsu, S.; Kawanami, T.; Tsutsumi, Y.; Hamaguchi, Y.; Horikawa, T.; Yoshinaga, Y.; Yamashita, S.; Tanaka, T.; et al. Exendin-4, a Glucagonlike Peptide-1 Receptor Agonist, Attenuates Breast Cancer Growth by Inhibiting NF-ΚB Activation. *Endocrinology* **2017**, *158*, 4218–4232, doi:10.1210/EN.2017-00461.

96. Tanaka, Y.; Iwaya, C.; Kawanami, T.; Hamaguchi, Y.; Horikawa, T.; Shigeoka, T.; Yanase, T.; Kawanami, D.; Nomiyama, T. Combined Treatment with Glucagon-like Peptide-1 Receptor Agonist Exendin-4 and Metformin Attenuates Breast Cancer Growth. *Diabetol Int* **2022**, *13*, 480–492, doi:10.1007/s13340-021-00560-z.

97. Tsutsumi, Y.; Nomiyama, T.; Kawanami, T.; Hamaguchi, Y.; Terawaki, Y.; Tanaka, T.; Murase, K.; Motonaga, R.; Tanabe, M.; Yanase, T. Combined Treatment with Exendin-4 and Metformin Attenuates Prostate Cancer Growth. *PLoS One* **2015**, *10*, e0139709, doi:10.1371/journal.pone.0139709.

98. Dhillon, S. Semaglutide: First Global Approval. *Drugs* **2018**, *78*, doi:10.1007/s40265-018-0871-0.

99. Rodbard, H.W.; Lingvay, I.; Reed, J.; De La Rosa, R.; Rose, L.; Sugimoto, D.; Araki, E.; Chu, P.L.; Wijayasinghe, N.; Norwood, P. Semaglutide Added to Basal Insulin in Type 2 Diabetes (SUSTAIN 5): A Randomized, Controlled Trial. *Journal of Clinical Endocrinology and Metabolism* **2018**, *103*, doi:10.1210/jc.2018-00070.

100. Chudleigh, R.A.; Bain, S.C. Semaglutide Injection for the Treatment of Adults with Type 2 Diabetes. *Expert Rev Clin Pharmacol* **2020**, doi:10.1080/17512433.2020.1776108.

101. Lee, S.; Lee, D.Y. Glucagon-like Peptide-1 and Glucagon-like Peptide-1 Receptor Agonists in the Treatment of Type 2 Diabetes. *Ann Pediatr Endocrinol Metab* **2017**, *22*, doi:10.6065/apem.2017.22.1.15.

102. Mahabaleshwarkar, R.; DeSantis, A. Metformin Dosage Patterns in Type 2 Diabetes Patients in a Real-World Setting in the United States. *Diabetes Res Clin Pract* **2021**, *172*, doi:10.1016/j.diabres.2020.108531.

103. Wilding, J.P.H.; Batterham, R.L.; Calanna, S.; Davies, M.; Van Gaal, L.F.; Lingvay, I.; McGowan, B.M.; Rosenstock, J.; Tran, M.T.D.; Wadden, T.A.; et al. Once-Weekly Semaglutide in Adults with Overweight or Obesity. *New England Journal of Medicine* **2021**, *384*, doi:10.1056/nejmoa2032183.

104. Rubino, D.M.; Greenway, F.L.; Khalid, U.; O’Neil, P.M.; Rosenstock, J.; Sørrig, R.; Wadden, T.A.; Wizert, A.; Garvey, W.T.; Arauz-Pacheco, C.; et al. Effect of Weekly Subcutaneous Semaglutide vs Daily Liraglutide on Body Weight in Adults With Overweight or Obesity Without Diabetes. *JAMA* **2022**, *327*, 138, doi:10.1001/jama.2021.23619.

105. Røder, M.E. Clinical Potential of Treatment with Semaglutide in Type 2 Diabetes Patients. *Drugs Context* 2019, *8*.

106. Davies, M.; Færch, L.; Jeppesen, O.K.; Pakseresht, A.; Pedersen, S.D.; Perreault, L.; Rosenstock, J.; Shimomura, I.; Viljoen, A.; Wadden, T.A.; et al. Semaglutide 2·4 Mg Once a Week in Adults with Overweight or Obesity, and Type 2 Diabetes (STEP 2): A Randomised, Double-Blind, Double-Dummy, Placebo-Controlled, Phase 3 Trial. *The Lancet* **2021**, *397*, doi:10.1016/S0140-6736(21)00213-0.

107. Roglic, G.; Unwin, N.; Bennett, P.H.; Mathers, C.; Tuomilehto, J.; Nag, S.; Connolly, V.; King, H. The Burden of Mortality Attributable to Diabetes: Realistic Estimates for the Year 2000. *Diabetes Care* **2005**, *28*, 2130–2135, doi:10.2337/DIACARE.28.9.2130.

108. Lin, Y.; Sun, Z. Current Views on Type 2 Diabetes. *Journal of Endocrinology* 2010, *204*.

109. Colberg, S.R.; Sigal, R.J.; Fernhall, B.; Regensteiner, J.G.; Blissmer, B.J.; Rubin, R.R.; Chasan-Taber, L.; Albright, A.L.; Braun, B. Exercise and Type 2 Diabetes: The American College of Sports Medicine and the American Diabetes Association: Joint Position Statement. *Diabetes Care* 2010, *33*.

110. 5. Glycemic Targets. *Diabetes Care* **2016**, *39*, doi:10.2337/dc16-S008.

111. Sorli, C.; Harashima, S. ichi; Tsoukas, G.M.; Unger, J.; Karsbøl, J.D.; Hansen, T.; Bain, S.C. Efficacy and Safety of Once-Weekly Semaglutide Monotherapy versus Placebo in Patients with Type 2 Diabetes (SUSTAIN 1): A Double-Blind, Randomised, Placebo-Controlled, Parallel-Group, Multinational, Multicentre Phase 3a Trial. *Lancet Diabetes Endocrinol* **2017**, *5*, doi:10.1016/S2213-8587(17)30013-X.

112. Ahrén, B.; Masmiquel, L.; Kumar, H.; Sargin, M.; Karsbøl, J.D.; Jacobsen, S.H.; Chow, F. Efficacy and Safety of Once-Weekly Semaglutide versus Once-Daily Sitagliptin as an Add-on to Metformin, Thiazolidinediones, or Both, in Patients with Type 2 Diabetes (SUSTAIN 2): A 56-Week, Double-Blind, Phase 3a, Randomised Trial. *Lancet Diabetes Endocrinol* **2017**, *5*, doi:10.1016/S2213-8587(17)30092-X.

113. Ahmann, A.J.; Capehorn, M.; Charpentier, G.; Dotta, F.; Henkel, E.; Lingvay, I.; Holst, A.G.; Annett, M.P.; Aroda, V.R. Efficacy and Safety of Once-Weekly Semaglutide versus Exenatide ER in Subjects with Type 2 Diabetes (SUSTAIN 3): A 56-Week, Open-Label, Randomized Clinical Trial. In Proceedings of the Diabetes Care; 2018; Vol. 41.

114. Aroda, V.R.; Bain, S.C.; Cariou, B.; Piletič, M.; Rose, L.; Axelsen, M.; Rowe, E.; DeVries, J.H. Efficacy and Safety of Once-Weekly Semaglutide versus Once-Daily Insulin Glargine as Add-on to Metformin (with or without Sulfonylureas) in Insulin-Naive Patients with Type 2 Diabetes (SUSTAIN 4): A Randomised, Open-Label, Parallel-Group, Multicentre, Multinational, Phase 3a Trial. *Lancet Diabetes Endocrinol* **2017**, *5*, doi:10.1016/S2213-8587(17)30085-2.

115. Marso, S.P.; Bain, S.C.; Consoli, A.; Eliaschewitz, F.G.; Jódar, E.; Leiter, L.A.; Lingvay, I.; Rosenstock, J.; Seufert, J.; Warren, M.L.; et al. Semaglutide and Cardiovascular Outcomes in Patients with Type 2 Diabetes. *New England Journal of Medicine* **2016**, *375*, doi:10.1056/nejmoa1607141.

116. Pratley, R.E.; Aroda, V.R.; Lingvay, I.; Lüdemann, J.; Andreassen, C.; Navarria, A.; Viljoen, A. Semaglutide versus Dulaglutide Once Weekly in Patients with Type 2 Diabetes (SUSTAIN 7): A Randomised, Open-Label, Phase 3b Trial. *Lancet Diabetes Endocrinol* **2018**, *6*, doi:10.1016/S2213-8587(18)30024-X.

117. Lingvay, I.; Catarig, A.M.; Frias, J.P.; Kumar, H.; Lausvig, N.L.; le Roux, C.W.; Thielke, D.; Viljoen, A.; McCrimmon, R.J. Efficacy and Safety of Once-Weekly Semaglutide versus Daily Canagliflozin as Add-on to Metformin in Patients with Type 2 Diabetes (SUSTAIN 8): A Double-Blind, Phase 3b, Randomised Controlled Trial. *Lancet Diabetes Endocrinol* **2019**, *7*, doi:10.1016/S2213-8587(19)30311-0.

118. Zinman, B.; Bhosekar, V.; Busch, R.; Holst, I.; Ludvik, B.; Thielke, D.; Thrasher, J.; Woo, V.; Philis-Tsimikas, A. Semaglutide Once Weekly as Add-on to SGLT-2 Inhibitor Therapy in Type 2 Diabetes (SUSTAIN 9): A Randomised, Placebo-Controlled Trial. *Lancet Diabetes Endocrinol* **2019**, *7*, 356–367, doi:10.1016/S2213-8587(19)30066-X.

119. Capehorn, M.S.; Catarig, A.M.; Furberg, J.K.; Janez, A.; Price, H.C.; Tadayon, S.; Vergès, B.; Marre, M. Efficacy and Safety of Once-Weekly Semaglutide 1.0 Mg vs Once-Daily Liraglutide 1.2 Mg as Add-on to 1–3 Oral Antidiabetic Drugs in Subjects with Type 2 Diabetes (SUSTAIN 10). *Diabetes Metab* **2020**, *46*, doi:10.1016/j.diabet.2019.101117.

120. Kellerer, M.; Kaltoft, M.S.; Lawson, J.; Nielsen, L.L.; Strojek, K.; Tabak, Ö.; Jacob, S. Effect of Once-Weekly Semaglutide versus Thrice-Daily Insulin Aspart, Both as Add-on to Metformin and Optimized Insulin Glargine Treatment in Participants with Type 2 Diabetes (SUSTAIN 11): A Randomized, Open-Label, Multinational, Phase 3b Trial. *Diabetes Obes Metab* **2022**, *24*, doi:10.1111/dom.14765.

121. Frías, J.P.; Auerbach, P.; Bajaj, H.S.; Fukushima, Y.; Lingvay, I.; Macura, S.; Søndergaard, A.L.; Tankova, T.I.; Tentolouris, N.; Buse, J.B. Efficacy and Safety of Once-Weekly Semaglutide 2·0 Mg versus 1·0 Mg in Patients with Type 2 Diabetes (SUSTAIN FORTE): A Double-Blind, Randomised, Phase 3B Trial. *Lancet Diabetes Endocrinol* **2021**, *9*, doi:10.1016/S2213-8587(21)00174-1.

122. Seino, Y.; Terauchi, Y.; Osonoi, T.; Yabe, D.; Abe, N.; Nishida, T.; Zacho, J.; Kaneko, S. Safety and Efficacy of Semaglutide Once Weekly vs Sitagliptin Once Daily, Both as Monotherapy in Japanese People with Type 2 Diabetes. *Diabetes Obes Metab* **2018**, *20*, doi:10.1111/dom.13082.

123. Kaku, K.; Yamada, Y.; Watada, H.; Abiko, A.; Nishida, T.; Zacho, J.; Kiyosue, A. Safety and Efficacy of Once-Weekly Semaglutide vs Additional Oral Antidiabetic Drugs in Japanese People with Inadequately Controlled Type 2 Diabetes: A Randomized Trial. *Diabetes Obes Metab* **2018**, *20*, doi:10.1111/dom.13218.

124. Ji, L.; Dong, X.; Li, Y.; Li, Y.; Lim, S.; Liu, M.; Ning, Z.; Rasmussen, S.; Skjøth, T.V.; Yuan, G.; et al. Efficacy and Safety of Once-Weekly Semaglutide versus Once-Daily Sitagliptin as Add-on to Metformin in Patients with Type 2 Diabetes in SUSTAIN China: A 30-Week, Double-Blind, Phase 3a, Randomized Trial. *Diabetes Obes Metab* **2021**, *23*, doi:10.1111/dom.14232.

125. Feng, Z.; Tong, W.K.; Zhang, X.; Tang, Z. Cost-Effectiveness Analysis of Once-Daily Oral Semaglutide versus Placebo and Subcutaneous Glucagon-like Peptide-1 Receptor Agonists Added to Insulin in Patients with Type 2 Diabetes in China. *Front Pharmacol* **2023**, *14*, doi:10.3389/fphar.2023.1226778.

126. Chen, J.; Yin, D.; Dou, K. Intensified Glycemic Control by HbA1c for Patients with Coronary Heart Disease and Type 2 Diabetes: A Review of Findings and Conclusions. *Cardiovasc Diabetol* 2023, *22*.

127. Hausner, H.; Derving Karsbøl, J.; Holst, A.G.; Jacobsen, J.B.; Wagner, F.D.; Golor, G.; Anderson, T.W. Effect of Semaglutide on the Pharmacokinetics of Metformin, Warfarin, Atorvastatin and Digoxin in Healthy Subjects. *Clin Pharmacokinet* **2017**, *56*, doi:10.1007/s40262-017-0532-6.

128. Bennett, W.L.; Maruthur, N.M.; Singh, S.; Segal, J.B.; Wilson, L.M.; Chatterjee, R.; Marinopoulos, S.S.; Puhan, M.A.; Ranasinghe, P.; Block, L.; et al. Comparative Effectiveness and Safety of Medications for Type 2 Diabetes: An Update Including New Drugs and 2-Drug Combinations. *Ann Intern Med* 2011, *154*.

129. Lambson, J.E.; Flegal, S.C.; Johnson, A.R. Administration Errors of Compounded Semaglutide Reported to a Poison Control Center—Case Series. *Journal of the American Pharmacists Association* **2023**, *63*, doi:10.1016/j.japh.2023.06.017.

130. Bethel, M.A.; Patel, R.A.; Merrill, P.; Lokhnygina, Y.; Buse, J.B.; Mentz, R.J.; Pagidipati, N.J.; Chan, J.C.; Gustavson, S.M.; Iqbal, N.; et al. Cardiovascular Outcomes with Glucagon-like Peptide-1 Receptor Agonists in Patients with Type 2 Diabetes: A Meta-Analysis. *Lancet Diabetes Endocrinol* **2018**, *6*, doi:10.1016/S2213-8587(17)30412-6.

131. Smits, M.M.; Van Raalte, D.H. Corrigendum: Safety of Semaglutide. *Front Endocrinol (Lausanne)* **2021**, *12*, doi:10.3389/fendo.2021.786732.

132. Nagendra, L.; BG, H.; Sharma, M.; Dutta, D. Semaglutide and Cancer: A Systematic Review and Meta-Analysis: Semaglutide and Cancer. *Diabetes and Metabolic Syndrome: Clinical Research and Reviews* **2023**, *17*, doi:10.1016/j.dsx.2023.102834.

133. Hansen, B.B.; Nuhoho, S.; Ali, S.N.; Dang-Tan, T.; Valentine, W.J.; Malkin, S.J.P.; Hunt, B. Oral Semaglutide versus Injectable Glucagon-like Peptide-1 Receptor Agonists: A Cost of Control Analysis. *J Med Econ* **2020**, *23*, doi:10.1080/13696998.2020.1722678.
